# A Cell‐Penetrant Peptide Disrupting the Transcription Factor CP2c Complexes Induces Cancer‐Specific Synthetic Lethality

**DOI:** 10.1002/advs.202305096

**Published:** 2023-10-16

**Authors:** Seung Han Son, Min Young Kim, Sungwoo Choi, Ji Sook Kim, Yong Sang Lee, Sangwon Lee, Yeon Ju Lee, Jin Youn Lee, Seol Eui Lee, Young Su Lim, Dae Hyun Ha, Eonju Oh, Young‐Bin Won, Chang‐Jun Ji, Mi Ae Park, Boram Kim, Kyu Tae Byun, Min Sung Chung, Jaemin Jeong, Dongho Choi, Eun Jung Baek, Eung‐Ho Cho, Sang‐Bum Kim, A. Reum Je, Hee‐Seok Kweon, Hyun Sook Park, Dongsun Park, June Sung Bae, Se Jin Jang, Chae‐Ok Yun, Ji Hyung Chae, Jin‐Won Lee, Su‐Jae Lee, Chan Gil Kim, Ho Chul Kang, Vladimir N. Uversky, Chul Geun Kim

**Affiliations:** ^1^ Department of Life Science and Research Institute for Natural Sciences College of Natural Sciences Hanyang University Seoul 04763 South Korea; ^2^ Department of Pathology Hanyang University College of Medicine Seoul 04763 South Korea; ^3^ Department of Bioengineering College of Engineering Hanyang University Seoul 04763 South Korea; ^4^ Department of Biotechnology and Research Institute for Biomedical and Health Science College of Biomedical and Health Science Konkuk University Chungju Chungbuk 27478 South Korea; ^5^ Department of Surgery Hanyang University College of Medicine Seoul 04763 South Korea; ^6^ Department of Laboratory Medicine Hanyang University College of Medicine Seoul 04763 South Korea; ^7^ Department of Surgery Korea Institute of Radiological and Medical Sciences Seoul 01812 South Korea; ^8^ Center for Research Equipment Korea Basic Science Institute Cheongju 28119 South Korea; ^9^ CEFO Co. Ltd. Seoul 03150 South Korea; ^10^ Department of Biology Education Korea National University of Education Cheongju Chungbuk 29173 South Korea; ^11^ Department of Research and Development OncoClew Co. Ltd Seoul 04778 South Korea; ^12^ Department of Pathology Asan Medical Center University of Ulsan College of Medicine Seoul 05505 South Korea; ^13^ Asan Center for Cancer Genome Discovery Asan Institute for Life Sciences Seoul 05505 South Korea; ^14^ Department of Molecular Medicine and USF Health Byrd Alzheimer`s Research Institute Morsani College of Medicine University of South Florida Tampa FL 33612 USA; ^15^ CGK Biopharma Co. Ltd. Seoul 04763 South Korea; ^16^ Present address: FNCT Biotech Co. Ltd. Seoul 04626 South Korea; ^17^ Present address: Department of Biomedical Sciences Graduate School of Ajou University School of Medicine Department of Physiology Ajou University School of Medicine Suwon Gyeonggi‐do 16499 South Korea

**Keywords:** a cell‐penetrant peptide, CP2c complex disruption, pan‐anticancer drug, synthetic lethality, transcription factor CP2c

## Abstract

Despite advances in precision oncology, cancer remains a global public health issue. In this report, proof‐of‐principle evidence is presented that a cell‐penetrable peptide (ACP52C) dissociates transcription factor CP2c complexes and induces apoptosis in most CP2c oncogene‐addicted cancer cells through transcription activity‐independent mechanisms. CP2cs dissociated from complexes directly interact with and degrade YY1, leading to apoptosis via the MDM2‐p53 pathway. The liberated CP2cs also inhibit TDP2, causing intrinsic genome‐wide DNA strand breaks and subsequent catastrophic DNA damage responses. These two mechanisms are independent of cancer driver mutations but are hindered by high MDM2 p60 expression. However, resistance to ACP52C mediated by MDM2 p60 can be sensitized by CASP2 inhibition. Additionally, derivatives of ACP52C conjugated with fatty acid alone or with a CASP2 inhibiting peptide show improved pharmacokinetics and reduced cancer burden, even in ACP52C‐resistant cancers. This study enhances the understanding of ACP52C‐induced cancer‐specific apoptosis induction and supports the use of ACP52C in anticancer drug development.

## Graphical abstract

Cancer‐specific synthetic lethality is demonstrated by targeting transcription factor CP2c with ACP52C, a cell‐penetrant peptide. ACP52C induces apoptosis, genomic instability, and G2/M arrest in most cancers, regardless of their driver mutations. Resistance in MDM2 p60‐expressing cells is overcome with CASP2 inhibition. Improved derivatives of ACP52C reduce cancer burden, supporting pan‐anticancer drug development.

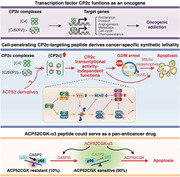

## 1 Introduction

Despite significant advancements in molecular and cancer biology, which have led to revolutionary changes in cancer treatment paradigms and the development of sophisticated anticancer therapeutics, cancer continues to be a major global public health problem. This is primarily due to tumor heterogeneity and the likelihood of later recurrence. Cancer cells differ from normal cells in their uncontrolled proliferation and survival, as well as their ability to invade or spread to various parts of the body, driven by mutations that result in constitutive activation of intracellular signaling pathways.^[^ ^1^ ^]^ While many recently approved anticancer drugs target specific oncogenic driver mutations, critical challenges in the battle against cancer persist. These challenges include metastatic spread, the growing resistance of cancer cells to available therapies, and the side effects that these therapies can have on normal cells.^[^ ^2^ ^]^ Additionally, therapeutics that target pan‐essential genes may be associated with limiting on‐target toxicities and difficulties in patient stratification. The abundance of information related to cancer also presents new challenges for cancer target discovery. Therefore, it is essential to establish an ideal anticancer therapeutic approach that selectively eliminates cancer cells without harming normal cells and effectively inhibits the growth of metastatic or drug‐resistant cancer stem cells.^[^ ^3^ ^]^

Importantly, cancer cells sometimes exhibit oncogene addiction, where specific oncogenic pathways are essential for their proliferation and survival. This makes them more susceptible to pharmacologic inhibition of a transcription factor (TF) or cofactor that acts genome‐wide. By targeting a TF like CP2c, therapeutic vulnerabilities can be introduced and exploited through synthetic lethal strategies. These interventions can also lead to the rewiring of regulatory, signaling, and metabolic networks, thereby opening up new therapeutic opportunities.^[^ ^4^ ^]^

CP2c, also known as TFCP2, CP2, α‐CP2, UBP‐1, SEF‐1, LSF, or LBP1C, is an evolutionarily conserved TF^[^ ^5^ ^]^ and a founding member of the CP2 family of TFs.^[^ ^6^ ^]^ It exerts its transcriptional function within two alternative complexes: the CP2c homotetramer ([C4], tCP2c), and the dimeric heterohexamer of CP2c, CP2b, and PIAS1 ([C2B2P2]2, CBP2),^[^ ^6^, ^7^ ^]^ which are tightly regulated by proteostasis through SUMOylation‐mediated PSME3‐20S proteasomal degradation.^[^ ^8^ ^]^ Normally, CP2c is expressed ubiquitously at low levels and plays a crucial role in cell proliferation, cell cycle, and differentiation, including processes like hematopoiesis, immune response, and neural development.^[^ ^5^, ^9^ ^]^ However, CP2c is significantly overexpressed in multiple specific cancers^[^ ^6^, ^9^, ^10^ ^]^ and has been linked to oncogenic addiction in cancer cells as a passenger oncogene,^[^ ^10a^ ^]^ regulating various aspects of cancer biology such as cell proliferation, cell cycle control, stemness, epithelial‐mesenchymal transition (EMT), metastasis, invasion, angiogenesis, and chemoresistance.^[^ ^10^, ^11^ ^]^ Elevated CP2c levels in patients have been associated with worse prognoses in hepatocellular carcinomas (HCCs),^[^ ^12^ ^]^ suggesting that CP2c could be a promising target for anticancer therapy.^[^ ^10^, ^11^ ^]^

We hypothesized that targeting the oncogenically addicted CP2c protein and regulating its activity could lead to the development of effective agents. In this context, it is worth noting that our prior investigations, which involved various CP2c binding peptide motifs, revealed that one peptide, Pep #5, effectively obstructs the DNA binding of CP2c. Interestingly, we found that the cell‐penetrating form of Pep #5 variant, Pep #5‐2C, exhibited strong anticancer activity. We have named this peptide “Anti‐Cancer Peptide #5‐2 penetrating Cell membrane” (ACP52C). Building on these insights, we provide a more comprehensive understanding of how ACP52C orchestrates cancer‐specific apoptosis. This effect encompasses G2/M arrest, genomic instability, and apoptosis across a broad spectrum of cancer types, all the while operating through transcription‐independent pathways and remaining unaffected by distinct driver mutations within the cancer cells.

## 2 Results

### 2.1 A Peptide (Pep #5‐2) Efficiently Inhibits the DNA Binding and Transcription Activity of CP2c

Previously, we discovered that among five different CP2c binding peptide motifs (Pep #5, #8, #13, #21, and #31), only Pep #5, which binds to the CP2c tetramerization domain (TD),^[^ ^13^ ^]^ effectively inhibits CP2c's DNA binding in a dose‐dependent manner in vitro (Figure S1A, Supporting Information).^[^ ^14^ ^]^ Furthermore, we observed that Pep #5 efficiently inhibits CP2c function in vivo in two different systems. First, the supplementation of Pep #5C, a cell‐penetrant form of Pep #5 that includes both terminal blocking and iRGD conjugation,^[^ ^15^ ^]^ to the culture, efficiently inhibited erythroid differentiation of murine erythroleukemia (MEL) cells (Figure S1B,C, Supporting Information). These cells serve as a well‐established in vitro model for chemical‐induced erythroid differentiation, demonstrating functional hemoglobin synthesis with cell cycle arrest at the G1 stage.^[^ ^16^ ^]^ The addition of hexamethylene bisacetamide (HMBA) to the culture can upregulate CP2c expression by 2‐3‐fold, which is crucial for functional globin expression and terminal erythroid differentiation.^[^ ^9^, ^17^ ^]^ Second, Pep #5 exhibited potent growth inhibition activity in several CP2c‐overexpressing human cancer cell lines (Figure S1D,E, Supporting Information). These findings suggest that Pep #5 can efficiently inhibit the DNA binding of CP2c to a degree sufficient to control CP2c‐related biological functions in vivo.

To determine if all 12 residues of Pep #5 are necessary for its activity, we conducted a DNA‐IP assay using two peptides, one with the N‐terminal half truncated (named Pep #5‐1) and the other with the C‐terminal half truncated (named Pep #5‐2), each consisting of six amino acid residues. We found that Pep #5‐2 inhibited the DNA binding activity of CP2c, similar to Pep #5, in a concentration‐dependent manner, whereas Pep #5‐1 showed no activity (Figure S2A,B, Supporting Information).

Crucially, in DNA‐IP assays, Pep #5‐2 inhibited the DNA binding activity of CP2c, CP2b, and PIAS1 in a concentration‐dependent manner, similar to Pep #5 (Figure S2B,C, Supporting Information). This suggests that Pep #5‐2 inhibits the DNA binding of both CP2c complexes, tCP2c and CBP complexes.^[^ ^6^, ^7^ ^]^ Furthermore, the cell‐penetrating variant, Pep #5‐2C, replicated the in vivo function of Pep #5, including the inhibition of erythroid differentiation and cancer cell growth (Figure S2D,E, Supporting Information). It is worth noting that Pep #5‐2C exhibited potent growth inhibition activity in cancer cells, and henceforth, we refer to Pep #5‐2 as ACP52 and Pep #5‐2C as ACP52C.

### 2.2 ACP52C Drives the Pan‐Anticancer Effect

Considering that ACP52 was chosen without a prior systematic investigation to determine whether it represents the minimal size of a peptide with anticancer activity, we conducted experiments using truncated and substitution mutants of ACP52 (up to a concentration of 10 µm) to assess their impact on cell proliferation in representative cancer cells (Figure S3A, Supporting Information). Our findings indicate that ACP52C is the agent with the lowest GI_50_ (50% growth inhibition) values, although peptides containing the central YPQR sequences exhibit variable effectiveness depending on cell lines (Figure S3B–E, Supporting Information). Furthermore, we confirmed that the growth inhibition activity of ACP52C is solely attributed to the Pep #5‐2 sequences. This was evident as ACP52D, which contains another CPP called dNP2^[^ ^18^ ^]^ or ACP52C conjugated with additional Cy5 fluorochrome, exhibited GI_50_ values similar to that of ACP52C (Figure S4, Supporting Information).

**Figure 1:**
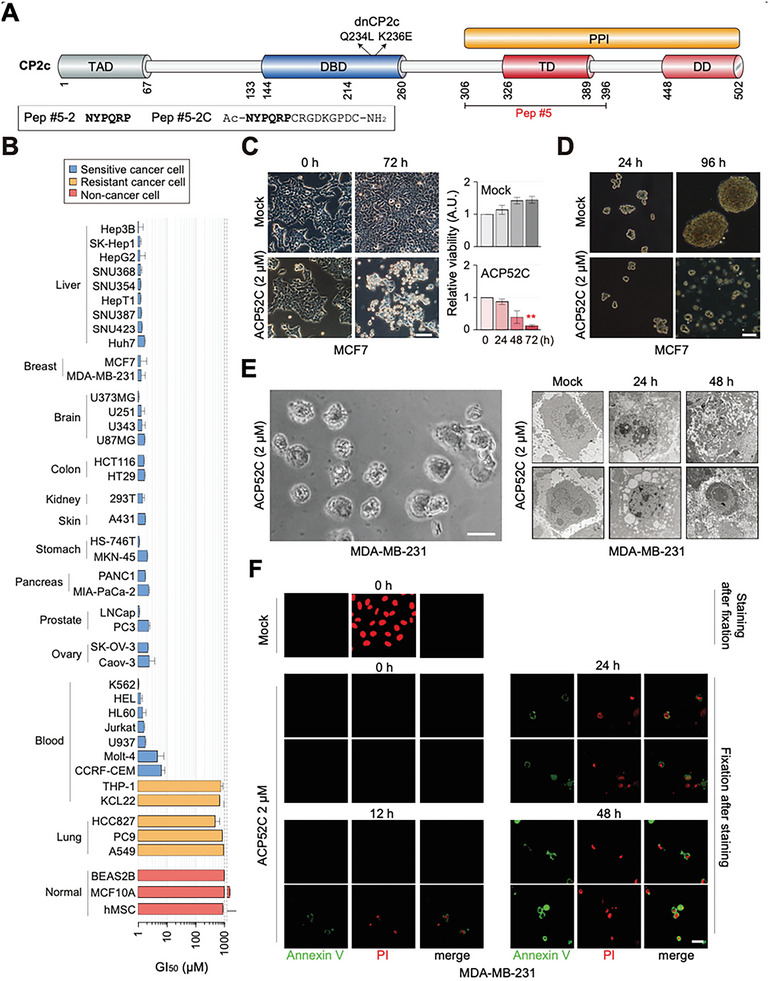
Efficient induction of cell death by cell‐penetrant Pep #5‐2 (ACP52C) in most of cancer cells. A) Schematic diagram illustrating the functional domains of the CP2c protein and the binding region of Pep #5. The lower left box displays amino acid sequences and the N‐ and C‐terminal modifications of Pep #5‐2, a truncated form of Pep #5, and its derivatives. TAD, transactivation domain; DBD, DNA binding domain; TD, tetramerization domain; DD, dimerization domain; PPI, protein‐protein interaction; dnCP2c, dominant negative form of human CP2c (Q234L and K236E). B) GI_50_ values of ACP52C in various human cancer and non‐cancer cells. Duplicated data are expressed as means ± SD. C) Cell photographs (left) and quantification of cell survival (right) demonstrate the cancer cell‐killing effect of ACP52C. Duplicated data are expressed as means ± SD. Two‐tailed unpaired Student's *t‐*test; **p < 0.01. Scale bar represents 50 µm. D) ACP52C exhibits anticancer effects on mammosphere‐cultured MCF7 cells. Scale bar represents 50 µm. Additional data can be found in Figure S6 (Supporting Information). E) Representative photographs showing gross cell morphology (left) and subcellular structures observed through electron microscopy (right) in MDA‐MB‐231 cells treated with 2 µm ACP52C for 48 h. Scale bar represents 20 µm. F) Immunofluorescent microscopic images reveal apoptotic cell death in ACP52C‐treated MDA‐MB‐231 cells. Scale bar represents 10 µm.

When we extended our tests to cells of diverse origins, ACP52C induced growth inhibition in most of the tested cancer cell lines in a concentration‐dependent manner, with GI_50_ values below 10 µm. However, lung cancer cells and some blood cancer cells exhibited resistance to ACP52C even at concentrations up to 1 mm (**Figure** **1**A,B). Importantly, in the initial study, normal cells derived from humans or mouse did not show any growth inhibition when exposed to ACP52C at concentrations up to 10 µm (Figures S3F and S4B,C, Supporting Information). Furthermore, ACP52C demonstrated minimal to zero growth inhibition, even at a concentration of 1 mm, when tested against human stem cells (hMSCs and hESCs), mouse embryonic fibroblasts (MEFs), and human non‐transformed cell lines (BEAS‐2B and MCF10A) (Figure 1B). This strongly suggests that ACP52C is unlikely to induce adverse effects in normal living cells.

It is important to highlight the distinctive characteristics of ACP52C. First, ACP52C rapidly penetrates the cell membrane and sequentially localizes in the cytosol, nucleus, and cytosol again before undergoing degradation within 16 h (Figure S5A, Supporting Information). This indicates that ACP52C is effectively delivered into the nucleus with minimal degradation in the cytoplasm, targeting nuclear CP2c within 8 h of treatment. Furthermore, the induction of growth inhibition in cancer cells occurs 48 h after a single supplementation of ACP52C to the culture medium and within a narrow range of ACP52C concentrations (Figure S5B,C, Supporting Information). This suggests a unique kinetic pattern of ACP52C action in cancer cells. Importantly, regardless of culture condition, whether conventional 2D or the mammosphere forming 3D, supplementation of ACP52C to the culture medium ultimately leads to cell death in a cancer cell‐specific manner (Figure 1C,D; Figure S6A,B, Supporting Information). ACP52C also demonstrated effectiveness in targeting cisplatin‐resistant cancer cells (Figure S6C, Supporting Information). Therefore, ACP52C holds promise as a potential treatment for various cancer types, including refractory metastatic cancers. Lastly, gross and electron microscopy of ACP52C‐treated cancer cells suggests cell death via apoptosis (Figure 1E). Annexin V and propidium iodide staining of dead cells (Figure 1F) further support this conclusion. Overall, the data indicate that ACP52C efficiently induces cell death in most of cancer cells without significant side effects.

### 2.3 ACP52C Induces G2/M Arrest, Genomic Instability, and Apoptosis

**Figure 2:**
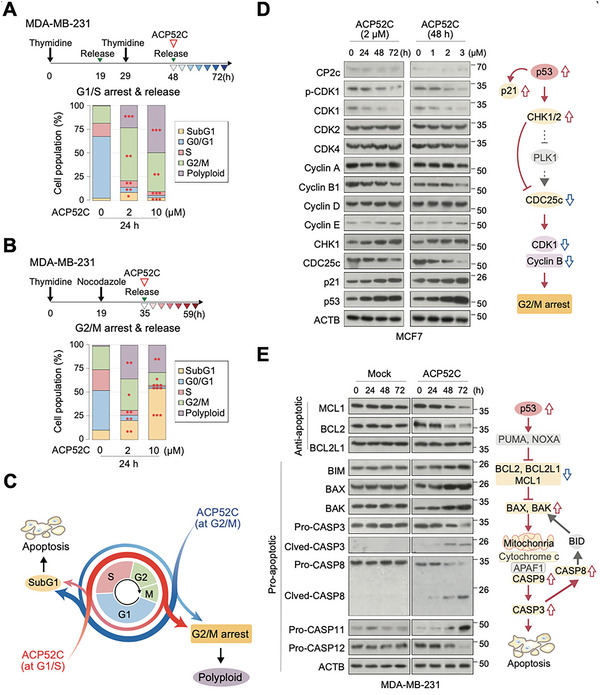
ACP52C induces cancer cell synthetic lethality through G2/M arrest, genomic instability, and apoptosis. A,B) Cell cycle distribution in synchronized cell populations treated with ACP52C over time. Experimental schematics (upper panels) and the cell cycle distribution in cells (lower panels) demonstrate the effect of ACP52C on cell cycle progression in synchronized cells at the G1/S boundary through thymidine double block (A) or at the G2/M boundary via thymidine/nocodazole treatment (B). Duplicated data are expressed as means ± SD. ANOVA test; *p < 0.05; **p < 0.01; ***p < 0.001. Refer to Figure S7B,C (Supporting Information) for the original data. C) Schematic depicting the emergence of polyploidy and the subG1‐fraction of cells in synchronized cell populations following ACP52C treatment over time. D) WBs display the expression of cell cycle‐related markers over time or at varying concentrations of ACP52C treatment in MCF7 cells (left), along with a proposed pathway leading to G2/M arrest (right). E) WBs illustrate the expression of apoptosis‐related markers over time in mock‐treated and 2 µm ACP52C‐treated cells (left), accompanied by a proposed pathway leading to apoptosis (right). Additional data can be found in Figure S7D (Supporting Information).

To comprehend the underlying mechanisms of ACP52C‐mediated growth inhibition and induction of cell death in cancer cells, we conducted an analysis of cell cycle progression following ACP52C treatment. In the asynchronous state, ACP52C led an increase in the number of cells in the subG1 phase, accompanied by a decrease in the number of cells in the G0/G1 and S phases as observed in fluorescence‐activated cell sorting (FACS) analysis (Figure S7A, Supporting Information). When ACP52C was administered to the cells released from the synchronization at the G1/S border, it induced G2/M arrest, resulting in a significant increase in the polyploidy fraction over time in a concentration‐dependent manner (**Figure** **2**A; Figure S7B, Supporting Information). Similarly, ACP52C caused a gradual transition of cell population from the polyploidy fraction to the subG1 fraction over time in cells synchronized at the G2/M border (Figure 2B; Figure S7C, Supporting Information). These results suggest that ACP52C sequentially triggers G2/M arrest, mitotic catastrophe, and ultimately, cell death (Figure 2C).

To identify the key cell cycle marker(s) affected by ACP52C, we assessed the expression of cell cycle markers over time or varying concentrations of ACP52C through Western blotting (WB). Our findings revealed that ACP52C induced G2/M phase arrest by downregulating G2/M checkpoint regulators, namely CDK1 and Cyclin B1, without affecting other phase checkpoint regulators (Figure 2D). Additionally, p53, p21, and CHK1 were upregulated, while CDC25c was downregulated, suggesting that ACP52C activates a signaling pathway for the G2/M phase checkpoint, in conjunction with p53 upregulation (Figure 2D). Although numerous signaling pathways converge on p53 to induce various cellular stress responses aimed at promoting cell cycle arrest and repair,^[^ ^19^ ^]^ our data indicate that ACP52C induces p53 protein upregulation, resulting in G2/M arrest, mitotic catastrophe, and cell death (as observed in subG1 phase cells).

To delve into the mechanism underlying cell death, we examined the expression of anti‐ and pro‐apoptotic makers through WBs. We observed a concurrent downregulation of anti‐apoptotic markers (MCL1 and BCL2) along with an upregulation of pro‐apoptotic markers (BIM, BAX, and BAK) and the activation of apoptosis‐related caspases (Figure 2E; Figure S7D, Supporting Information). These findings suggest that ACP52C induces a p53‐mediated intrinsic apoptosis program. However, it is worth noting that CASP8, an initiator caspase of extrinsic apoptosis that functions as a molecular switch for apoptosis, necroptosis, and pyroptosis,^[^ ^20^ ^]^ is activated. Additionally, CASP11 and CASP12, which are involved in the non‐canonical inflammatory pathway (i.e., pyroptosis), are up‐ and down‐regulated, respectively. These data indicate that apoptosis is the primary driving force behind cell death, with intriguing interplay between pathways such as apoptosis, necroptosis, and pyroptosis.

### 2.4 ACP52C Instigates Cancer‐Specific Apoptosis Not by Modulating CP2c Transcriptional Activity, but Through Liberating Free CP2c Isoforms

Initially, we anticipated that ACP52C‐mediated induction of cancer cell apoptosis would occur through the deregulation of CP2c target gene expression, given that ACP52C targets CP2c. Our WB results indicated elevated CP2c expression in most of the cancer cell lines (Figure S8A, Supporting Information), consistent with prior reports of prominent CP2c overexpression (OE) in some human cancers.^[^ ^10^, ^12^ ^]^ To identify CP2c target genes critical for apoptosis induction, we conducted RNA sequencing and analyzed differentially expressed genes (DEGs) following ACP52C treatment (Figure S8B, Supporting Information). Interestingly, most of the CP2c transcriptional target genes remained unaltered upon ACP52C treatment, and there were no common DEGs between the two cell lines exhibiting similar expression patterns over time (Figure S8C, Supporting Information). Although 126 DEGs were shared between both cell lines as a whole, with some significant gene ontology terms (Figure S8D, Supporting Information), no significant enrichment of genes was observed (Figure S8E, Supporting Information). Upon manually curating the DEGs associated with growth inhibition and apoptosis induction, we identified a few common pathways and genes shared by the two cell lines (Figure S8F–I, Supporting Information). Importantly, although these common DEGs were upregulated in response to ACP52C treatment in both cell lines, siRNA‐mediated knock‐down (KD) of each gene abrogated the effects of ACP52C, leading to an increase in GI_50_ values (Figure S8J, Supporting Information). This suggests that these genes are not CP2c transcriptional targets but play a role in ACP52C sensitivity.

**Figure 3:**
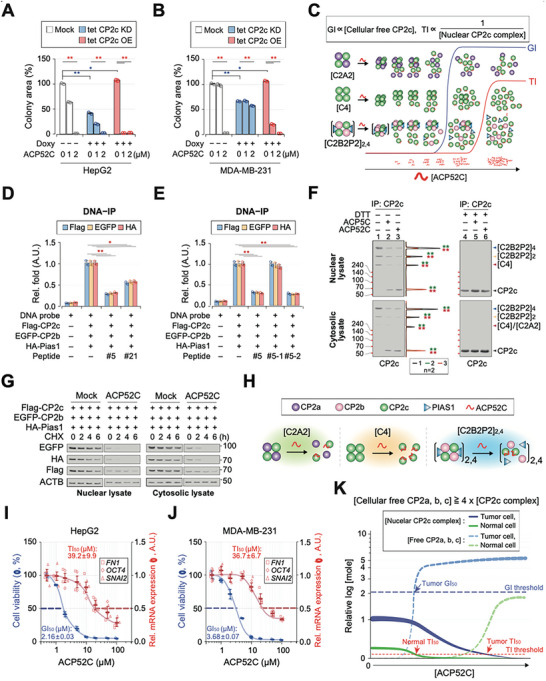
ACP52C dissociates CP2c complexes and induces apoptosis in a CP2c transcriptional activity‐independent manner. A,B) Colony‐forming ability tests of ACP52C in HepG2 (A) and MDA‐MB‐231 (B) cells with varying expression levels of CP2c. Duplicated data are expressed as means ± SD. Two‐tailed unpaired Student's *t‐*test; *p < 0.05; **p < 0.01. C) Schematic illustrating the effect of ACP52C on cell growth and transcription inhibition through the dissociation of CP2c complexes. D–H) Pep #5‐2 disrupts CP2c TF complexes. D,E) DNA‐IP assays demonstrate that Pep #5‐2 inhibits the DNA binding activity of the homotetrameric [C4] and heteromeric CBP ([C2B2P2]_2, 4_) complexes to the wildtype *Hba‐a2* promoter probe, similar to Pep #5. Data are means ± SD of two independent biological replicates. Two‐tailed unpaired Student's *t‐*test; **p < 0.01; *p < 0.05. F) DSP XL‐WB shows efficient disruption of the endogenous nuclear and cytosolic CP2c complexes by Pep #5‐2C. Band intensities were quantified from two independent biological replicates. Two‐tailed unpaired Student's *t‐*test; **p < 0.01. G) Pep #5‐2C induces instability of CP2c complex proteins in the nucleus (top) and cytosol (bottom), inhibiting CP2c complex formation. New protein synthesis was inhibited by cycloheximide (CHX) treatment. H) Schematic depicting how Pep #5‐2 disrupts CP2c TF complexes. Refer to Figure S10 (Supporting Information) for additional data. I,J) Growth inhibition occurs at lower concentration of ACP52C in cancer cells compared to transcriptional inhibition of CP2c targets. Cell viability and relative mRNA levels of CP2c transcriptional targets (*FN1*, *OCT4*, and *SNAI2*) were assessed in HepG2 (I) and MDA‐MB‐231 (J) cells at 36 h after ACP52C treatment through MTT assays and RT‐qPCR, respectively. GI_50_ and TI_50_ (50% transcription inhibition) values were estimated from the cell growth and transcription inhibition profiles, respectively. Duplicated data are expressed as means ± SD. K) Schematic depicting the induction kinetics of cell growth inhibition and transcription inhibition in a specific cell type following ACP52C treatment, where cell growth inhibition positively correlates with the concentrations of total cellular free CP2c isoforms, while transcription inhibition inversely correlates with the functional nuclear CP2c TF complexes.

To provide further evidence supporting the transcription activity‐independent anticancer effect of ACP52C, we conducted additional experiments. We observed a positive correlation between the colony‐forming ability of cancer cells, which is associated with their anchorage‐independent growth capacity and correlated with the expression level of CP2c,^[^ ^10a^ ^]^ and their sensitivity to ACP52C (**Figure** **3**A,B). This suggests that cancer cells with higher CP2c expression are more susceptible to ACP52C‐induced cell death. Furthermore, we found that CP2c regulated most of EMT genes at the transcriptional level (Figure S9A, Supporting Information), but ACP52C treatment did not affect these genes (Figure S9B,C, Supporting Information). Lastly, ACP52C did not exhibit interactions with over 450 human kinases or disease‐relevant mutant variants in the KINOMEscan screening. This suggests that the in vivo function of ACP52C is not mediated indirectly through protein phosphorylation or by off‐target effects. These data support the notion that ACP52C induces apoptosis in cancer cells through an unforeseen mechanism, independent of CP2c transcriptional activity.

To elucidate the transcription activity‐independent function of ACP52C, we hypothesized that cancer‐specific apoptosis is induced through the release of CP2c monomers from CP2c complexes upon ACP52C treatment, rather than relying on the transcriptional activity of CP2c complexes themselves (Figure 3C). We speculate that Pep #5, which binds to the CP2c TD (Figure 1A), dissociates CP2c complexes, rendering them unable to bind to DNA and activate transcription. Furthermore, ACP52C treatment leads to a more than four‐fold increase in the concentration of CP2c monomers, at the expense of one CP2c complex (Figure 3C), which may contribute to cell growth inhibition within a narrow range of ACP52C concentrations (Figure S5C, Supporting Information).

To investigate whether ACP52 could dissociate CP2c complexes, we conducted in vitro IP and pulldown assays using Pep #5 and Pep #5‐2. We observed that both peptides efficiently dissociated CP2c complexes, regardless of the presence of probe DNA (Figure 3D,E; Figure S10, Supporting Information). Additionally, we conducted in vivo assays using Dithiobis (succinimidyl propionate) (DSP) crosslinking‐Western blot (XL‐WB)^[^ ^7b^ ^]^ and WB in the presence of new protein synthesis inhibition (Figure 3F,G). These experiments demonstrated that ACP52C treatment readily dissociated all endogenous nuclear and cytoplasmic CP2c complexes, generating a significant amount of CP2c monomers. These monomers were subsequently degraded through the SUMO1/PSME3/20S proteasome axis.^[^ ^8^ ^]^ These findings indicate that ACP52C treatment generates a considerable amount of CP2c monomers from CP2c complexes in cells (Figure 3H).

Our study revealed that CP2c dissociation from CP2c complexes is mediated by the binding of Pep #5‐2 to one side of the CP2c TD surface in the CP2c‐CP2c interaction junctions of CP2c complexes. Several pieces of evidence support this mechanism (Figures S11 and S12, Supporting Information). 1) We could not detect a stable CP2c‐Pep #5‐2 complex in surface plasmon resonance analyses, suggesting that the interaction between Pep #5‐2 and CP2c is transient or unstable. 2) XL‐mass spectrometry analyses revealed specific crosslinking between Pep #5‐2 and three C‐terminal amino acids of CP2c peptides (R339, R376, and R402), situated on one side of the CP2c TD surface (Figure S11, Supporting Information). 3) Although Pep #5‐2 showed limited cross‐reactivity with other CP2c isoforms (CP2a and CP2b), it displayed higher coverage (97.7%) with CP2c due to the high sequence conservation and corresponding positions in their predicted tertiary structures. 4) We identified specific amino acid residues (E332, R339, R341, and R347) crucial for Pep #5‐2 binding within the CP2c TD. These residues were also important for DNA binding of CP2c (Figure S12A,C,D, Supporting Information). 5) Docking with HADDOCK further supported this model, indicating that residues important for Pep #5‐2 binding and for CP2c complex formation are located in the CP2c interaction junctions (Figure S12E, Supporting Information). 6) Our model was validated by testing additional mutations (W336V and A380F), which were expected to reside in interfaces important for complex formation but not for Pep #5‐2 binding (Figure S12C,D,F,I, Supporting Information). In summary, Pep #5‐2 binds to the four CP2c‐CP2c interaction junctions, interfering with tCP2c formation. We anticipate that Pep #5‐2 also disrupts the CBP complexes by binding to both CP2c‐CP2b and CP2c‐CP2c interaction junctions, although further study is required.

Given this background information, we conducted a comparison of the concentration‐dependent mode of ACP52C action in terms of growth inhibition (GI) and transcriptional inhibition (TI) of the representative CP2c transcription targets (*FN1*, *OCT4*, and *SNAI2*) in two cancer cell lines, 36 h after ACP52C treatment (Figure 3I,J). In both cases, the average TI_50_ (50% transcriptional inhibition) values (≈35–40 µm) of the three CP2c targets were considerably higher than the GI_50_ values (≈2–4 µm), suggesting that ACP52C‐mediated cell death induction is attributed to the action of CP2c monomers rather than CP2c complexes. It is therefore expected that the threshold for GI would occur at lower ACP52C concentration than that for TI in CP2c‐addicted cancer cells since ACP52C (2 µm; equivalent to the GI_50_ value) could generate enough CP2c monomers to induce cell death, whereas disruption of CP2c complexes alone may be insufficient to elicit the transcriptional activity of CP2c (Figure 3K).

In this context, normal cells expressing low levels of CP2c are expected to liberate only small amounts of monomeric CP2c, which may not be sufficient to trigger cell death even when exposed to the same amount of ACP52C. This explanation helps clarify the cancer‐specific anti‐cancer effect of ACP52C. The relationship between GI_50_ and TI_50_ values for α‐globin mRNA expression in MEL cells also supports this hypothesis (Figures S1B,C, S2D, and S13A,C, Supporting Information). HMBA‐uninduced MEL cells (uMEL), which have low levels of CP2c and do not express the α‐globin gene, showed a GI_50_ value of ≈40 µm, while HMBA‐induced MEL cells (iMEL), which express the α‐globin gene due to increased levels of CP2c isoforms,^[^ ^9^, ^17^ ^]^ exhibited TI_50_ and GI_50_ values of ≈15 and ≈17 µm, respectively (Figure S13A–C, Supporting Information).

Furthermore, it is worth noting that modulation of cellular CP2b concentration, but not that of PIAS1, also affected the GI_50_ values of ACP52C in cancer cells (Figure S13D–G, Supporting Information). This finding supports the idea that both CP2c isoforms, CP2a and CP2b, function as members of CP2c complexes and possess conserved TD sequences to which ACP52C binds,^[^ ^7b^ ^]^ and thus, their monomers liberated by ACP52C treatment could also be involved in this process. Overall, our data suggest that ACP52C‐mediated cancer‐specific apoptosis occurs through the free CP2c isoforms released by ACP52C treatment, independently of CP2c transcriptional activity.

### 2.5 The CP2c/YY1/MDM2 p90/p53 Pathway is Involved in ACP52C‐Medited Cancer‐Specific Apoptosis Induction

**Figure 4:**
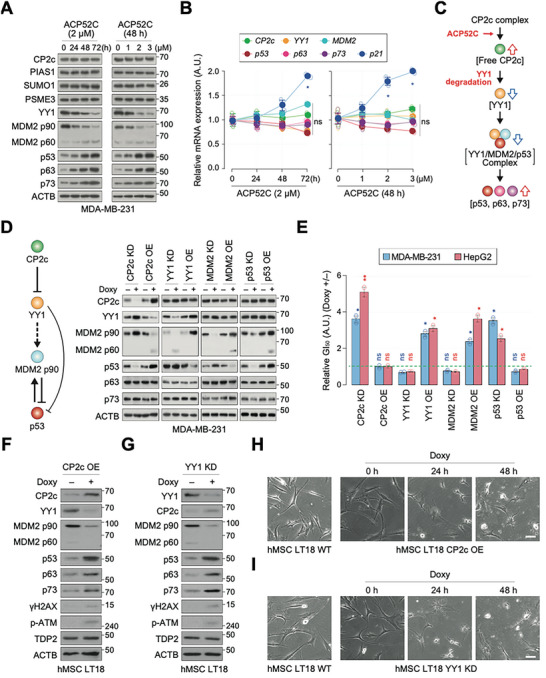
ACP52C induces cancer‐specific apoptosis via the CP2c/YY1/MDM2 p90/p53 pathway. A–E) ACP52C induces cancer‐specific apoptosis through the CP2c/YY1/MDM2 p90/p53 pathway. A) WBs and B) RT‐qPCR depict the expression profiles of the p53‐ and CP2c‐centered markers upon ACP52C treatment over time or concentration. Duplicated data are expressed as means ± SD. Two‐tailed unpaired Student's *t‐*test; *p < 0.05; ns, non‐significant. C) Hypothesis of apoptosis induction by ACP52C‐mediated dissociation of CP2c complexes through the CP2c/YY1/MDM2 p90/p53 pathway. D) WBs display the modulation of proteins in MDA‐MB‐231 cells with Tet‐inducible KD or OE of each CP2c, YY1, MDM2 p90, or p53. E) ACP52C‐mediated GI_50_ values in cells with KD or OE of each factor in MDA‐MB‐231 and HepG2 cells. Duplicated data are expressed as means ± SD. Two‐tailed unpaired Student's *t‐*test; *p < 0.05; **p < 0.01; ns, non‐significant. Refer to Figure S14 (Supporting Information) for additional data, including the original WBs in HepG2 cells. F–I) YY1/MDM2 p90/p53 axis‐mediated cell death induction is also effective in hMSCs. F,G) WB show marker protein expression in hMSCs 48 h after conditional CP2c OE (F) or YY1 KD (G). H,I) Representative photographs of cells during conditional CP2c OE (H) or YY1 KD (I). Scale bar represents 50 µm.

To investigate the molecular mechanism underlying ACP52C‐mediated induction of apoptosis through CP2c monomers, we conducted an analysis of changes in the expression of markers involved in p53‐ and CP2c‐centered signaling pathways in cancer cells treated with ACP52C. While most of these markers remained unaffected by ACP52C treatment, we observed a downregulation of the p53 regulators YY1 and MDM2 p90 at the protein level (**Figure** **4**A–C; Figure S14A, Supporting Information).^[^ ^1b^ ^]^ Notably, this downregulation occurred at the protein level but not at the mRNA level. YY1 plays a crucial role in regulating the transcriptional activity, acetylation, ubiquitination, and stability of p53. It does so by inhibiting p53's interaction with the coactivator p300 and by enhancing its interaction with the negative regulator MDM2 p90.^[^ ^21^ ^]^ MDM2 p90, an E3 ubiquitin ligase, exerts its influence by binding to p53's trans‐activation domain and promoting its degradation,^[^ ^22^ ^]^ thereby forming an autoregulatory feedback loop with p53.^[^ ^23^ ^]^

We demonstrated the existence of a pathway starting from CP2c, involving sequential downregulation of YY1 and MDM2 p90, followed by upregulation of p53 in cells with KD or OE of each factor, as confirmed by WB analyses (Figure 4D; Figure S14B, Supporting Information). We also confirmed the involvement of this pathway in ACP52C‐mediated growth inhibition and apoptosis (Figure 4E). Importantly, this pathway was also found to be functional in human mesenchymal stem cells (hMSCs), despite lower CP2c expression levels compared to cancer cells. CP2c OE or YY1 KD induced spontaneous cell death in hMSCs (Figure 4F–I). These findings suggest that ACP52C exploits the intrinsic CP2c/YY1/MDM2 p90/p53 pathway for cancer cell‐specific apoptosis induction, and that high levels of CP2c monomers are responsible for this phenomenon.

We discovered that CP2c interacts directly with YY1,^[^ ^13^ ^]^ but not with MDM2 p90 or p53 (Figure S15A,B, Supporting Information). CP2c is not involved in transcriptional activation of *YY1* (Figure S15C); instead, it induces YY1 degradation via 20S proteasome.^[^ ^12^ ^]^ On the other hand, although CP2c can activate the transcription of *MDM2* (Figure S15D–H, Supporting Information), this CP2c‐mediated transcriptional activity of *MDM2* is not related to ACP52C‐mediated MDM2 p90 downregulation (Figure S15I, Supporting Information). YY1, as a TF, binds to *MDM2* regulatory regions (Figure S15E–H, Supporting Information) but is not involved in transcriptional activity of the *MDM2* gene (Figure S15D, Supporting Information). This suggests that YY1‐mediated modulation of MDM2 p90 protein occurs at the posttranscriptional level through an as‐yet‐unknown mechanism. We also found that ACP52C‐mediated upregulation of p53 occurs via posttranscriptional downregulation of YY1 and MDM2 p90 (Figure 4A–D; Figure S14, Supporting Information), although p53 could transcriptionally modulate *YY1* and *MDM2* expression (Figure S15C,D, Supporting Information). Therefore, ACP52C induces cancer cell apoptosis by liberating CP2c monomers from CP2c complexes, which leads to sequential downregulation of YY1 and MDM2 p90 and upregulation of p53 at posttranscriptional level.

### 2.6 The Catastrophic DNA Damage Responses Through TDP2 Squelching is also Involved in ACP52C‐Mediated Cancer‐Specific Apoptosis Induction

**Figure 5:**
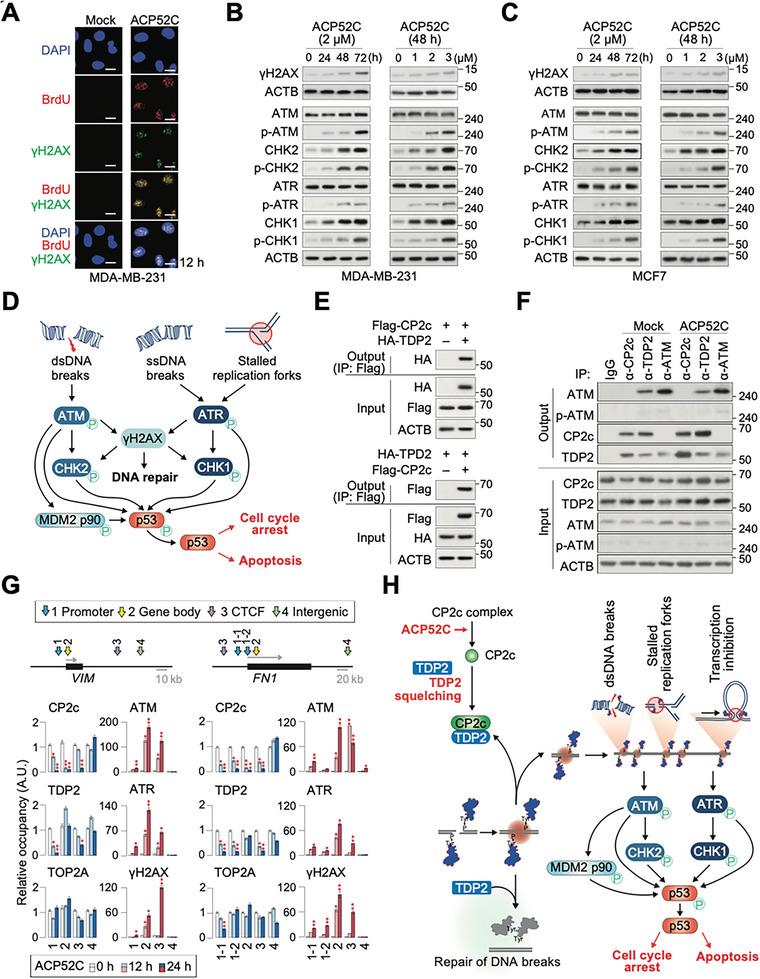
ACP52C induces cancer‐specific apoptosis through TDP2 squelching‐associated catastrophic DNA damage responses. A–D) DNA strand break‐associated DNA damage response (DDR) mechanisms are linked to ACP52C responses in cancer cells. A) Confocal immunofluorescence microscopy shows the activation of γH2AX, a sensitive molecular marker of DNA damage and repair, and DNA synthesis upon ACP52C treatment, suggesting the involvement of DDR pathways. B,C) WBs depict occurrences of DDR in MDA‐MB‐231 (B) and MCF7 (C) cells following ACP52C treatment over time or concentration. Scale bar in (A) represents 5 µm. D) Schematic representation of DDR pathways. E–G) Monomeric CP2cs bind TDP2 to squelch its TOP2 depoisoning function, leading to catastrophic DDR. E) IP assays demonstrate the direct interaction between ectopically overexpressed CP2c and TDP2 in 293T cells. F) Co‐IPs reveal the enhanced interaction between endogenous CP2c and TDP2 in MDA‐MB‐231 cells upon ACP52C treatment. G) ChIP‐qPCRs show the ACP52C‐mediated modulation of DDR factors binding around the representative CP2c target genes, *VIM* (left) and *FN1* (right). Duplicated data are expressed as means ± SD. Two‐tailed unpaired Student's *t‐*test; *p < 0.05; **p < 0.01. H) Schematic illustration of a catastrophic DDR cascade triggered by ACP52C‐induced TDP2 squelching.

In ACP52C‐treated cancer cells, we also observed an increase in DNA strand breaks (SBs) and an upregulation of DNA damage responses (DDRs) markers, including γH2AX, as well as phosphorylated ATM, ATR, CHK1, and CHK2 (**Figure** **5**A–C). Since ATM is typically activated in response to DNA double strand breaks (DSBs), and ATR responds to a broad spectrum of DNA damage that generates single‐stranded DNA, the simultaneous upregulation of phosphorylated ATM and ATR in ACP52C‐treated cancer cells suggests that ACP52C may induce extensive DNA SBs (Figure 5D). Therefore, it is suggested that, in addition to activating the CP2c/YY1/MDM2 p90/p53 pathway, catastrophic DDRs may contribute to ACP52C‐mediated cancer‐specific apoptosis induction.

As a driving force behind massive DNA DSBs and the induction of DDR in ACP52C‐mediated cancer‐specific apoptosis, we identified TDP2 (also known as TTRAP or EAPII).^[^ ^24^ ^]^ This identification was made through yeast two‐hybrid assays^[^ ^7a^ ^]^ as well as confirmed by in vivo co‐IP analyses (Figure 5E; Figure S16A, Supporting Information). TDP2 is a 5‐tyrosyl DNA phosphodiesterase that plays a crucial role in repairing DNA adducts generated by non‐productive (abortive) activity of topoisomerase II (TOP2).^[^ ^24^, ^25^ ^]^

We hypothesized that ACP52C treatment liberates CP2c monomers that bind to and inhibit TDP2, leading to the accumulation of DNA SBs due to intrinsic genome‐wide TOP2 poisoning and the induction of catastrophic DDR. Our findings support this hypothesis, as TDP2 KD cancer cells exhibited reduced cell proliferation, disturbed cell cycle profiles, and increased sensitivity to ACP52C compared to mock cells (Figure S16B–E, Supporting Information). Notably, TDP2 expression was not influenced by CP2c, YY1, MDM2 p90, or p53 (Figure S16F–I, Supporting Information), but the TDP2‐CP2c interaction was significantly enhanced by ACP52C treatment, whereas TDP2 could interact with both CP2c and ATM in the normal state (Figure 5F). Importantly, TDP2 interacts with the liberated CP2c monomers but not with CP2c in the CP2c complexes (Figure S16J left, Supporting Information), confirming the squelching of TDP2 by CP2c monomers. Interestingly, YY1 was also observed to interact exclusively with the liberated CP2c monomers induced by ACP52C treatment (Figure S16J right, Supporting Information), suggesting that the accelerated degradation of YY1 might also be attributed to this interaction.

Furthermore, we observed that ACP52C treatment induces the dissociation of CP2c and TDP2 from the promoters, gene bodies, and CTCF binding sites of selected CP2c target genes, resulting in the accumulation of ATM, ATR, and γH2AX in these regions (Figure 5G). Interestingly, these effects were not observed in intergenic inert regions. Considering that TDP2 squelching may also occur during DNA replication fork movement, we speculate that ACP52C‐induced TDP2 squelching can cause transcriptional pausing and replication stress, leading to catastrophic DDRs and subsequent activation of checkpoint induction mechanisms involving various DNA‐damage effectors and p53.^[^ ^26^ ^]^ Eventually, this cascade leads to apoptosis (Figure 5H).

The N‐terminal phosphorylation of p53 at Ser15 and Ser20 by ATM, ATR, DNA‐PK, CHK1, and CHK2 is known to stabilize p53 by inhibiting its interaction with MDM2 p90.^[^ ^27^ ^]^ Therefore, ACP52C plays a crucial role in stabilizing p53 via both the TDP2‐initiated DDR and the YY1/MDM2 p90‐mediated mechanisms. Overall, our findings suggest that ACP52C induces cancer‐specific apoptosis through the intricate interplay of the CP2c/YY1/MDM2 p90/p53 pathway and catastrophic DNA damage responses mediated by TDP2 squelching.

### 2.7 ACP52C Induces Cancer‐Specific Apoptosis via MDM2 p90 Downregulation, Irrespective of p53 Mutation Status

**Figure 6:**
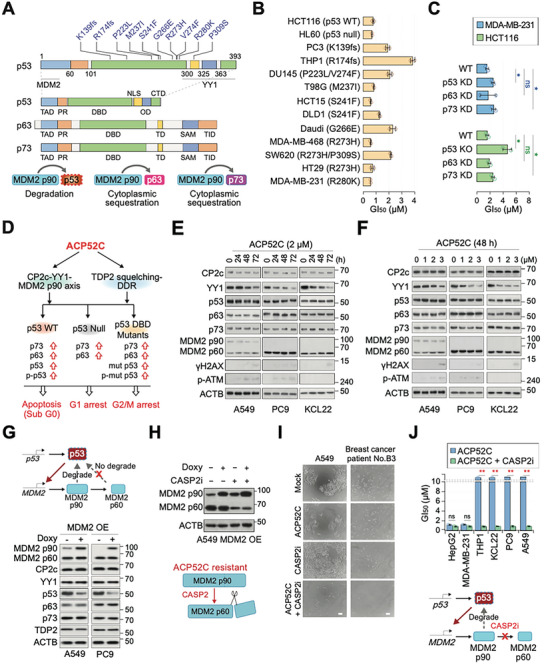
CASP2‐mediated MDM2 p90 cleavage is a factor in ACP52C resistance in some cancer cells, and sensitizing ACP52C resistance with CASP2 inhibition. A–D) ACP52C functions regardless of the p53 mutation status in cancer cells. A) Schematic of the p53 protein showing MDM2 p90‐ and YY1‐binding domains and mutation sites in some cancers (top panel). Comparative domain structures of p53 paralogs, p63 and p73 (middle panel), and differential MDM2 p90 effects on p53, p63, and p73 (bottom panel). CTD, C‐terminal domain; DBD, DNA binding domain; NLS, nuclear localization sequence; OD, oligomerization domain; PR, proline‐rich domain; SAM, sterile alpha motif; TAD, transcriptional activation domain; TID, transcriptional inhibition domain. B) ACP52C‐mediated GI_50_ values in various cancer cell lines with different p53 mutation statuses. Duplicated data are expressed as means ± SD. C) ACP52C‐mediated GI_50_ values in HCT116 and MDA‐MB‐231 cells with downregulation of either p53, p63, or p73. Duplicated data are expressed as means ± SD. Two‐tailed unpaired Student's *t‐*test; *p < 0.05; ns, non‐significant. D) Schematic representation of ACP52C‐mediated anticancer activity induction through MDM2 p90 downregulation, independent of p53 mutation status. E,F) Analysis of marker protein expression levels in resistant cells after ACP52C treatment over time (E) or concentration (F) by WB. G) Schematics of mutual regulation between p53 and MDM2 p90, where the MDM2 p60 form does not induce p53 degradation (top) and immunoblots showing marker protein expression in resistant cells with MDM2 p90 OE (bottom). H) Analysis of MDM2 p90 and p60 expression levels in resistant cells after CASP2 inhibiting peptide (CASP2i; Ac‐VDVAD‐CHO, 10 µm) treatment. I,J) ACP52C resistance can be sensitized by CASP2 inhibition. I) Photographs of A549 and human patient‐derived cultured cancer cells treated with ACP52C (2 µm) and CASP2i (10 µm), alone or in combination for 48 h. Scale bar represents 50 µm. J) GI_50_ values in representative cancer cell lines with ACP52C (2 µm) alone or in combination with CASP2i (10 µm) (top) and schematic showing the restoration of ACP52 sensitivity by preventing MDM2 p90 degradation via CASP2 inhibitor treatment (bottom). Duplicated data are expressed as means ± SD. Two‐tailed unpaired Student's *t‐*test; **p < 0.01; ns, non‐significant.

We demonstrated that ACP52C can induce apoptosis in a wide range of cancer cells, regardless of their oncogenic background or mutational load, including those with mutated *p53* genes (Figure 1B). The *p53* gene is frequently mutated in human cancer, with around 80% of mutations being protein‐altering missense mutations in the DNA‐binding domain (**Figure** **6**A, top). However, these mutations do not affect the N‐terminal transactivation domains, which can still be post‐transcriptionally regulated by MDM2 p90 and DNA damage‐mediated phosphorylation. Therefore, both wildtype and mutant p53 proteins can be stabilized by ACP52C treatment through both MDM2 p90/YY1 downregulation and ATM/ATR‐mediated phosphorylation at Ser15.^[^ ^27^ ^]^

The p53 family comprises three proteins (Figure 6A, middle): p53, p63, and p73, which have both shared and distinct functions. Specifically, p63 and p73 can perform p53‐like activities by promoting the transcriptional activation of genes involved in cell growth and apoptosis. Importantly, unlike p53, mutations in the genes encoding p63 and p73 are infrequent in human cancer. Additionally, MDM2 p90, an E3 ligase that negatively regulates p53, can bind all three proteins, but the outcomes of their interactions differ. While MDM2 p90 promotes p53 degradation via ubiquitination, p63 and p73 are not degraded but are instead sequestered in the cytoplasm by MDM2 p90 (Figure 6A, bottom). This action prevents their interaction with p300/CBP and inhibits their transcriptional activities. Therefore, depletion of MDM2 p90 in p53‐mutant or ‐null cancers may induce cell death through upregulation of p63 and/or p73 proteins.

Our study showed that p53 is crucial for ACP52C‐mediated apoptosis induction in cancer cells with wildtype or R280K‐mutated p53, as p53 KD, specifically targeting residues 231–236 of p53, leading to a three to fourfold increase in GI_50_ (Figure 6B). However, we also observed that cancer cells undergoing apoptosis due to ACP52C treatment expressed higher levels of p63 and p73 proteins, regardless of their p53 mutation status (Figure 4A,D; Figure S14, Supporting Information). This upregulation of p63 and p73 proteins was also observed in hMSCs undergoing apoptosis through CP2c OE or YY1 KD (Figure 4F,G) but not in cancer cells (MDA‐MB‐231 cells with a p53 R280K mutation) with normal cell proliferation by CP2c OE or YY1 KD (Figure 4D; Figure S14B, Supporting Information). These observations suggest that the upregulation of p63 and p73 proteins in p53 mutant cancer cells by ACP52C treatment may play a crucial role in inducing apoptosis. Therefore, our findings suggest that ACP52C can induce apoptosis via MDM2 p90 downregulation in i) wildtype p53‐bearing cancers through p53 upregulation and phosphorylation, ii) p53‐null cancers through p63 and p73 upregulation, and iii) mutant p53‐bearing cancers through p63 and p73 upregulation. Remarkably, our results are consistent with recent findings that MDM2 p90 depletion leads to cancer cell death regardless of p53 mutation status.^[^ ^28^ ^]^

The efficiency of ACP52C‐mediated growth inhibition in mutant p53‐bearing cancers could be further modulated by the upregulation and phosphorylation of mutant p53, as well as other cellular contexts resulting from specific p53 mutations. We found that ACP52C‐induced growth inhibition occurred regardless of p53 mutation status in general, and GI_50_ values in mutant p53‐bearing cancers showed only slight deviations among them, which were higher than in p53 wildtype or null cancers (Figure 6B). Moreover, both p53 and p73 downregulation reduced ACP52C sensitivity in HCT116 and MDA‐MB‐231 cells (Figure 6C). Our results indicate that ACP52C exerts its anticancer effect independently on the p53 mutation status, although the extent of ACP52C‐mediated anticancer activity induction could be modulated by p53 substitution mutations (Figure 6D).

### 2.8 MDM2 p90‐CASP2 Signaling Governs ACP52C Resistance

ACP52C exhibits anticancer activity in most cancer cell lines, but not in all of them (Figure 1B). To investigate the mechanism of resistance to ACP52C, we initially examined the expression profiles of potential candidate proteins in ACP52C‐resistant cell lines using WB analysis (Figure 6E,F). We specifically observed a significant increase in the expression of MDM2 p60, accompanied by a decrease in MDM2 p90, in the resistant cells. In contrast, ACP52C‐sensitive cells predominantly expressed MDM2 p90 (Figure 4A; Figure S14A, Supporting Information). Importantly, the expression of p53 family proteins was not upregulated in the ACP52C‐treated resistant cells, and the downregulation of MDM2 p60 did not occur either (Figure 6E,F).

Considering the autoregulatory feedback loop between p53 and MDM2 p90, which helps maintain p53 at normalized basal levels and promotes cell survival, as well as the role of MDM2 p60 in creating a p53‐induced positive feedback loop with transforming activity (Figure 6G, top), we speculated that the resistance to ACP52C originated from the deregulation of the MDM2 p90‐p53 axis due to MDM2 p60 OE. To validate this hypothesis, we investigated whether ectopically introducing the full‐length *MDM2* cDNA could lead to the degradation of p53 protein in the ACP52C‐resistant cells. As anticipated, MDM2 p90 OE led to the destabilization of p53 protein, while the levels of p63 and p73 proteins remained unaffected (Figure 6G, bottom). Additionally, we confirmed that Caspase 2 (CASP2), an apoptosis‐independent protein, was involved in the production of MDM2 p60, as previously reported.^[^ ^29^ ^]^ This process did not involve alternative splicing or single nucleotide polymorphisms (Figure S17, Supporting Information). Furthermore, we observed a positive correlation between the content of MDM2 p60 and CASP2 activity in ACP52C resistance in cancer cells (Figure S18, Supporting Information). Although the role of CASP2 in regulating p53 stability during the cell cycle and DDR is still debated in different cancer contexts,^[^ ^30^ ^]^ treatment of the resistant cells with a CASP2 inhibiting peptide (Ac‐VDVAD‐CHO) resulted in higher levels of MDM2 p90 and simultaneous downregulation of MDM2 p60 (Figure 6H). Moreover, the combined treatment of ACP52C and the CASP2 inhibiting peptide sensitized ACP52C‐resistant cancer cell lines (Figure 6I,J) and human primary tumor cells to ACP52C (Figure 6I; Figure S19, Supporting Information). These results strongly suggest that CASP2 serves as a resistance factor for ACP52C therapy by cleaving MDM2 p90 into MDM2 p60, and targeting CASP2 holds promise in enhancing the therapeutic response to ACP52C.

### 2.9 Therapeutic Potential of ACP52C Derivatives in Mouse Tumor Models

The anticancer efficacy of ACP52C was assessed in vivo using cell line‐derived xenograft (CDX) mouse models implanted with MDA‐MB‐231 LM1 breast cancer, A431 epidermoid carcinoma, or U343 glioma cells. ACP52C was administered systemically via intravenous injection every 2 or 3 days, starting from day 14 after the subcutaneous injection of cancer cells. Importantly, treatment with ACP52C significantly reduced tumor weight in all cases (Figure S20A–C, Supporting Information). Furthermore, in repeated toxicity tests conducted on normal male and female mice, ACP52C did not exhibit any adverse side effects (Figure S20D, Supporting Information). These findings indicate that ACP52C holds promise as an effective anticancer and antimetastatic agent, with minimal risk of adverse effects on normal tissues. However, it is worth noting that ACP52C has a relatively short systemic metabolic stability in vivo, lasting ≈7.8 h, as observed in live imaging of Hep3B CDX mice following intravenous injection of Cy5‐ACP52C (Figure S20E, Supporting Information). This reduced stability is mainly attributed to renal secretion. Nonetheless, the tumor‐targeting ability of ACP52C remains prominent, as evidenced by fluorescence observed in dissected tumor tissue five days after Cy5‐ACP52C injection (Figure S20F, Supporting Information).

To address the issue of the short systemic half‐life of ACP52C, a fatty acid conjugation strategy was implemented. Fatty acids, whether free or conjugated to larger molecules, tend to bind to human serum albumin (HSA), which helps protect them from rapid renal filtration.^[^ ^31^ ^]^ Building upon this strategy, five additional fatty acid‐conjugated derivatives of ACP52C were devised (Figure S21A, Supporting Information). First, ACP52CG was engineered by linking hydrophobic C16 fatty acids (palmitic acid) to the εK NH3+ group of the lysine (K) residue at the N‐terminus of ACP52C. This derivative ensured cell permeability by distancing the fatty acid from iRGD. However, it also introduced the potential for self‐assembly, similar to micelles, due to the clustering of palmitic acids on one side. Second, ACP52CK was designed by incorporating an enzyme‐cleavable linker, GFLG, between ACP52C and palmitic acid. The GFLG tetrapeptide, widely used as a linker in the development of polymer‐drug conjugates,^[^ ^32^ ^]^ is sensitive to the lysosomal enzyme cathepsin B (CatB), typically present at a concentration of around 1 mm in lysosomes. Notably, high levels of cathepsin B are also found in various human cancers, often leading to secretion and association with the tumor cell membrane.^[^ ^33^ ^]^ ACP52CK was expected to be processed by CatB in the cancer microenvironment, yielding free ACP52C. Even if ACP52CK itself entered cells, it was assumed that it would be processed within lysosomes, ultimately producing active, free ACP52C. Third, we explored the attachment of palmitic acid in the middle of ACP52C, rather than at the terminus. Specifically, when coupling a fatty acid in the middle of the peptide, we referred to the strategy used for the GLP‐1 derivative NN2211 (Liraglutide).^[^ ^34^ ^]^ In this regard, Glu‐palmitic acid (E‐pal) was coupled to the lysine (K) residue located between ACP52 and iRGD. Considering that the impact on ACP52's activity might vary depending on the attachment direction, ACP52GK and ACP52CGK were derived by attaching E‐pal to glutamine's αE or γE COO‐ groups in ACP52, respectively. Lastly, to mitigate the self‐assembly of fatty acids, DHA, an unsaturated fatty acid, was employed to conjugate with the εK NH3+ group at the N‐terminus of ACP52C, resulting in DHA‐ACP52C. These diverse strategies were explored to address the short systemic half‐life of ACP52C and its potential implications.

In terms of peptide properties, aside from an increase in molecular weight, there were no significant changes observed (Figure S21A, Supporting Information). All derivatives appeared as white amorphous powder and exhibited excellent solubility in water, saline, and PBS with solubility extending up to 10 mm. Notably, ACP52CGK was found to completely dissolve even at concentrations as high as 100 mg mL^−1^. Regarding their growth inhibitory properties, all derivatives demonstrated effects similar to that of the original ACP52C in the selected cell lines (Figure S21B, Supporting information). As they exhibited comparable activity in aqueous solvents (data not shown), all peptides were dissolved in physiological saline for ease of use in in vivo experiments. For in vivo trials, the peptide doses were ascertained using the GI_50_ values of ACP52C (≈2 µm) confirmed in cell lines, and the derivatives were dosed accordingly. However, it is important to note that ACP52C and its derivatives have varying molecular weights, necessitating the analysis and interpretation of results while considering the number of molecules involved in each case.

**Figure 7:**
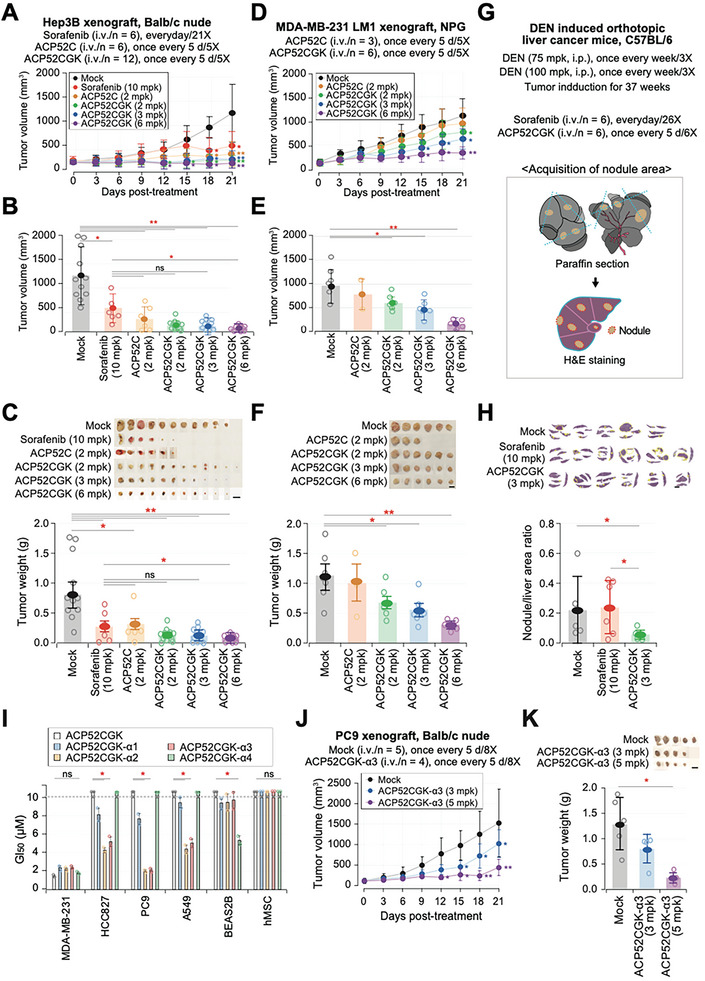
In vivo efficacies of ACP52C and its derivatives in the mouse tumor models. A–C) Therapeutic effects of ACP52C and ACP52CGK in Hep3B xenograft Balb/c nude mice. Tumor volumes during the treatment phase (A), tumor volumes measured immediately before sacrifice (B), and photographs of tumors and corresponding tumor weights in the sacrificed mice (C). Scale bar represents 10 mm. Data (means ± SE) were analyzed using ANOVA test. *p < 0.05; **p < 0.01. D‐F) Therapeutic effects of ACP52C and ACP52CGK in the MDA‐MB‐231 LM1 xenograft NPG mice. Tumor volumes during the treatment phase (D), tumor volumes measured immediately before sacrifice (E), and photographs of tumors and corresponding tumor weights in the sacrificed mice (F). Scale bar represents 10 mm. Data (means ± SE) were analyzed using ANOVA test. *p < 0.05; **p < 0.01. G,H) Therapeutic effects of ACP52CGK in DEN‐induced orthotopic liver cancer mice. Experimental schemes for tumor induction and the tumor nodule size estimation (G), and photographs of the H&E‐stained liver sections and the estimation of the tumor nodule size in the sacrificed mice (H). Data (means ± SE) were analyzed using ANOVA test. *p < 0.05. I–K) Cellular and in vivo efficacies of ACP52CGK‐α3, a CASP2 inhibiting peptide‐conjugated ACP52CGK. (I) GI_50_ values of ACP52CGK derivatives (α1 to α4; see Table S3 for sequences, Supporting Information) in the representative cell lines. Duplicated data are expressed as means ± SD. Two‐tailed unpaired Student's *t‐*test *p < 0.05. J,K) ACP52CGK‐α3 confers a therapeutic effect in the ACP52C‐resistant PC9 CDX Balb/c nude mouse. Anticancer efficacy was assessed by tumor volumes during the treatment phase (J) and tumor weights in the sacrificed mice (K). Data (means ± SE) were analyzed using ANOVA test. *p < 0.05; **p < 0.01. It is worth noting that there were no animal deaths related to drug administration in any of the animal studies, although there was one animal death in some treatment groups.

When evaluating their anti‐tumor effectiveness in Hep3B CDX mice, the results varied considerably among the derivatives (Figure S21C–F, Supporting Information). Hep3B CDX mice were systemically treated with ACP52C, ACP52CG, and ACP52CK via intravenous injection at three‐day intervals, and for comparison, Sorafenib,^[^ ^35^ ^]^ was administered orally once daily as a positive control. ACP52C demonstrated superior anti‐tumor activity compared to Sorafenib, while ACP52CG exhibited a decrease in anti‐tumor activity, resembling Sorafenib, possibly due to the potential formation of micelle‐like self‐assembled structures (Figure S21C, Supporting Information). Furthermore, ACP52CK, despite containing the CatB recognition sequence, displayed activity similar to that of Sorafenib (Figure S21C, Supporting Information). Interestingly, in Hep3B CDX mice administered at three‐day intervals, ACP52GK, with the fatty acid linked to αE COO‐ of glutamine, demonstrated lower anti‐tumor activity compared to ACP52C (Figure S21D, Supporting Information), whereas ACP52CGK, with the fatty acid linked to γE COO‐, exhibited activity similar to that of ACP52C (Figure S21E, Supporting Information). Notably, when administered at five‐day intervals, ACP52C displayed reduced anti‐tumor activity compared to the three‐day interval administration, resembling the activity of Sorafenib. However, ACP52CGK maintained its activity even when administered at five‐day intervals (**Figure** **7**A–C; Figure S21C–F, Supporting Information). DHA‐ACP52C, administered at three‐day intervals, effectively demonstrated anti‐tumor activity in Hep3B CDX mice (data not shown). Although not statistically significant when administered at five‐day intervals, it exhibited noticeably higher activity than ACP52C (Figure S21F, Supporting Information). Therefore, both DHA‐ACP52C and ACP52CGK effectively improved the short half‐life of ACP52C and demonstrated substantial anti‐tumor activity in vivo. However, due to the observed inferior yield or purity in DHA‐ACP52C, we decided to focus on ACP52CGK for further studies.

ACP52CGK demonstrated the same action mechanism as ACP52C and exhibited time‐dependent subcellular localization similar to ACP52C (Figure S22A,B, Supporting Information). Additionally, Cy5‐labeled ACP52CGK displayed improved systemic metabolic stability in Hep3B CDX mice (Figure S22C,D, Supporting Information). Moreover, rather than relying on indirect measurements through fluorescent markers, we developed a bioanalytical approach to directly measure the pharmacokinetics (PK) of 5 mg kg^−1^ (mpk) ACP52CGK in mice over 48 h and found a half‐life of ≈5.26 h at the peripheral blood (Figure S23, Supporting Information). Importantly, when administered at five‐day intervals, ACP52CGK demonstrated remarkable anti‐tumor efficacy not only in the Hep3B CDX model but also in the breast cancer models, such as the MDA‐MB‐231 LM1 CDX mouse models (Figure 7D–F), as well as in the diethylnitrosamine (DEN)‐induced orthotopic liver cancer mouse model (Figure 7G,H), while showing no apparent side effects (Figure S24, Supporting Information). Furthermore, we conducted repeated toxicity tests with ACP52CGK at a dosage of 100 mpk, which did not induce any adverse effects in normal organs such as brain, heart, and liver (Figure S25, Supporting Information). These findings are highly encouraging for the development of ACP52CGK‐based therapeutic anticancer drugs.

Furthermore, we generated a series of CASP2 inhibiting peptide‐conjugated derivatives of ACP52CGK, which successfully restored ACP52CGK resistance in cell culture systems without adversely affecting normal cells (Figure 7I). One of these derivatives, ACP52CGK‐α3, exhibited significant anticancer efficacy in the ACP52C‐resistant PC9 CDX mouse model (Figure 7J,K). This suggests that this cancer‐specific lethality provides a rationale for developing an ideal pan‐anticancer drug with no side effects on normal cells.

## 3 Discussion

**Figure 8:**
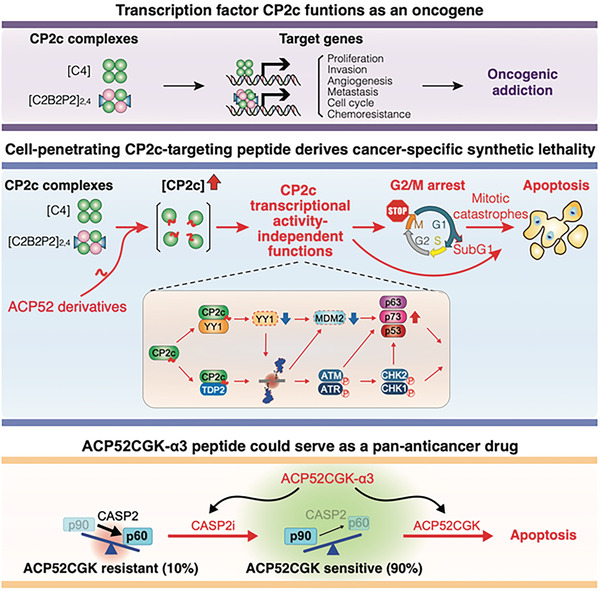
Schematic diagram depicting cancer‐specific apoptosis induced by ACP52C. This schematic diagram illustrates the multifaceted impact of ACP52C in inducing cancer‐specific apoptosis. In many cancers, elevated expression of the transcription factor CP2c is a distinguishing feature, indicating their reliance on CP2c for growth. ACP52C, a cell‐penetrating peptide, disrupts CP2c complexes, leading to cancer‐specific lethality through two unexpected transcription‐independent DNA damage response pathways triggered by the liberated CP2c monomers. Some cancer cells display resistance to ACP52C due to increased levels of MDM2 p60, a product of CASP2‐mediated cleavage of MDM2 p90. However, this resistance can be overcome by co‐treatment with a CASP2 inhibitor. This insight lays the groundwork for the development of potential pan‐anticancer drugs that have minimal effects on normal cells.

A myriad of cancer treatment options is available for cancer therapy, but they often encounter roadblocks such as side effects and the emergence of drug resistance. ACP52C offers a unique approach by targeting cancer cells with high CP2c expression, revealing vulnerabilities that can be exploited through synthetic lethal strategies. Mechanistically, ACP52C disrupts CP2c TF complexes, releasing CP2c monomers. These monomers then kick‐start two independent pathways: the YY1/MDM2/p53 pathway and the DDR pathway, achieved through YY1 degradation or TDP2 inhibition (**Figure** **8**). The direct binding of CP2c monomers leads to YY1 degradation, which subsequently causes downregulation of MDM2 and upregulation of p53 family proteins. In parallel, TDP2 squelching by direct binding to CP2c monomers evokes genome‐wide DNA SB accumulation, and in turn, DNA SBs activate ATM/ATR kinase phosphorylation leading to complicated cascades of cellular events, eventually leading to G2/M arrest, mitotic catastrophe, and cell death (subG1 fraction). Obviously, these two pathways crosstalk during execution, as can be seen in YY1 and p53 family proteins. While YY1 downregulation per se could induce p53 stabilization, it could exert its action indirectly by downregulating MDM2, and potentially by obstructing DSB repair. Consequently, cancer cells overexpressing CP2c become prone to lethality, G2/M arrest, genomic instability, and apoptosis, while normal cells exhibiting low CP2c expression either resist ACP52C‐induced cell death or show no detrimental effects.

One of the significant advantages of ACP52C is its capability to exert its anticancer effects independent of cancer driver mutations and p53 mutation status, due to compensatory role of p63 and p73. This feature enables ACP52C to effectively target cancer cells and overcome drug resistance commonly observed with traditional therapies. Preliminary data indicate that ACP52C shows sensitivity in over 85% of hepatocellular carcinoma and breast cancer patient‐derived cancer cells, demonstrating its potential as a pan‐anticancer drug. Our findings also suggest that ACP52C‐resistant cancer cells express higher levels of MDM2 p60 by the CASP2‐mediated cleavage of MDM2 p90. Importantly we demonstrated that concurrent treatment of ACP52C and CASP2 inhibitor could induce cell death in these resistant cells at both cellular and in vivo levels (Figure 8). Thus, by targeting upstream transcription factors, ACP52C minimizes side effects and shows considerable promise as a pan‐anticancer drug.

On the other hand, Factor Quinolinone Inhibitor 1 (FQI1), a small molecule identified for its specific inhibition of CP2c DNA‐binding, partner protein‐binding, and transactivation activities,^[^ ^36^ ^]^ effectively reduces cell proliferation with minimal detectable side effects in preclinical models.^[^ ^36^, ^37^ ^]^ Notably, in comparison to Pep #5‐2, FQI1 exhibits limited inhibitory effects on MEL cell differentiation in vitro (Figure S26A, Supporting Information) and demonstrates proficient inhibition of DNA binding with only slight perturbation of CP2c complexes (Figure S26B–F, Supporting Information). However, it is important to highlight that FQI1's potential to induce cell death, not just in resistant cancer cells but also in normal cells such as mesenchymal stem cells and embryonic stem cells (Figure S26G,H, Supporting Information), suggests a possibility of undesirable side effects, which ACP52C does not exhibit. Further studies are required to distinguish, complement, or validate the proposed mechanisms of FQI1 and ACP52C.

Current views on the repair versus apoptosis decision primarily stem from studies involving the DNA damage‐inducible p53. Cancers that inactivate the p53 tumor suppressor, either through mutations or deletions in the *p53* gene itself or indirectly via deregulation of upstream pathways, exhibit increased aggressiveness and resistance to various therapies.^[^ ^38^ ^]^ Therefore, to circumvent these kinds of cancers, novel strategies to deal with cellular p53 activity en masse are required. Notably, ACP52C‐sensitive cells show a simultaneous decrease in the MDM2 p90 levels and an increase in the levels of p53 family proteins, irrespective of the cellular p53 mutation status. Drugs that degrade MDM2 p90 selectively kill cancer cells with wildtype p53 and not the p53‐inactivated cancer cells,^[^ ^39^ ^]^ suggesting that stabilization of p53 family proteins is crucial for the ACP52C‐mediated apoptosis induction.

We confirmed that cellular protein levels of both p63 and p73 are related to ACP52C sensitivity in p53‐null or mutant cancers. However, manipulating YY1 or MDM2 p90 levels alone is insufficient to induce cell death accompanied by upregulation of p53 family proteins. The sensitivity of cells to ACP52C inversely correlated with the cellular YY1 and MDM2 p90 protein levels. Notably, altering YY1 or MDM2 p90 expression in hMSCs leads to cell death accompanied by upregulation of p53 proteins. Furthermore, ACP52C treatment increases the levels of all three p53 family proteins without affecting their mRNA levels. Lastly, ACP52C resistance also depends on the expression levels of p53 family proteins and MDM2 p90 protein. ACP52C‐resistant cells expressed higher levels of MDM2 p60 protein at the expense of MDM p90 protein via CASP2‐mediated cleavage, with no stabilization of p53 family proteins. We revealed that ACP52C resistance can be overcome by MDM2 p90 OE through the transfection of full‐length *MDM2* cDNA. These findings strongly suggest that the upregulation of the p53 family proteins directly triggers cell death, regardless of the cellular p53 mutation status, potentially involving additional factors related to the MDM2 p90 protein levels.

Since it is known that the MDM2 p90 phosphorylation by activated ATM or ATR stabilizes p53, whereas non‐phosphorylated MDM2 p90 does not show this effect,^[^ ^40^ ^]^ ACP52C‐treated cells likely employ an unidentified mechanism that efficiently regulates the interplay between the phosphorylated and non‐phosphorylated forms of MDM2 p90. On the contrary, YY1 serves not only as a transcription factor but also plays a vital role in promoting enhancer‐promoter chromatin loops through dimer formation and DNA interactions. Disruption of YY1 can impair enhancer‐promoter loops and gene expression,^[^ ^41^ ^]^ potentially leading to severe cellular consequences. Therefore, CP2c‐mediated YY1 degradation may contribute to genome‐wide transcriptional disruption, influencing cellular functions. Lastly, the significance of the PIDDosome complex as a CASP2 activation platform has been extensively demonstrated.^[^ ^30^ ^]^ However, its involvement in non‐apoptotic functions and the pathway leading to ACP52C resistance concerning MDM2 p90 cleavage necessitate thorough examination. Overall, in terms of ACP52C sensitivity in cancers, further investigation is required to unravel the cellular and molecular mechanisms defining the stabilization of p53 family proteins, phosphorylation and degradation of MDM2 p90, and generation of the MDM2 p60.

It is important to note that our findings are consistent with recent studies showing that, in triple‐negative breast cancer, MDM2 p90 elimination by MDM2‐PROTAC resulted in cell death regardless of the p53 mutation status, accompanied by the posttranscriptional stabilization of p73 but not p63.^[^ ^28^ ^]^ The upregulation of p73 was confirmed to be a consequence of MDM2 p90 depletion and was crucial for cell death induction, although the exact mechanism remains unknown. It was speculated that MDM2 p90 depletion completely eliminates its p53‐dependent and p53‐independent oncogenic effects, including p73 activation. Therefore, it is possible that common or similar pathways are involved in controlling the p73 protein upregulation induced by PROTAC or the upregulation of p53 family proteins induced by ACP52C.

To develop effective anticancer agents, ensuring their efficacy, safety, and stability is crucial, as these factors are key to the successful completion of preclinical trials. The efficacy of both ACP52C and its derivatives has been verified in various cancer models using CDX mice, and toxicity tests have confirmed the safety of ACP52C and ACP52CGK even at concentrations more than several tens of times higher than those demonstrating efficacy. However, we found that both ACP52C and ACP52CGK gradually lose their activity over one year when stored either in powder or solution form in a deep freezer. We identified that this reduced activity originated from the formation of nanoparticles among molecules through inter‐strand disulfide bonds between Cys residues (Figure S27, Supporting Information). We observed that the majority of ACP52C exists in the form of nanoparticles in saline (Figure S27A, Supporting Information), possibly due to the formation of inter‐strand disulfide bonds since two Cys residues exist in the iRGD sequence of each ACP52C molecule. Surprisingly, although still displaying larger‐sized particles compared to the monomer, ACP52CGK exhibited a significant reduction in nanoparticle formation upon palmitic acid conjugation in the middle of ACP52C (Figure S27A, Supporting Information). High‐Performance Liquid Chromatography (HPLC) analyses confirmed the presence of these larger particles in ACP52CGK over time (Figure S27B, Supporting Information). Notably, treatment with dithiothreitol (DTT) resulted in the elimination of these larger particles in Liquid Chromatography‐Mass Spectrometry (LC‐MS) (Figure S27C, Supporting Information), suggesting the presence of complexes stabilized by the inter‐strand disulfide bond formation involving cysteine in the iRGD sequence. Accordingly, addressing this issue will be a focus of future strategies if ACP52CGK and its derivatives are to be considered for clinical trials.

The discovery that ACP52C, which targets TF CP2c, exhibits a pan‐anticancer effect with minimal side effects on normal cells is highly intriguing. CP2c, functioning as a TF, plays a crucial role in regulating the expression of target genes involved in chemoresistance, angiogenesis, EMT, tumor‐initiating ability, and drug resistance, displaying an oncogenic addiction phenomenon. Therefore, it can be inferred that inhibiting the transcriptional activity of CP2c could lead to the demise of cancer cells through oncogenic shock. Furthermore, the fact that ACP52C exhibits anticancer effects through at least two distinct pathways suggests that it may have comparable efficacy to combined treatments with different anticancer agents, which is a part of the current efforts to overcome drug resistance in anticancer drug development. Lastly, we demonstrated that the combined treatment of ACP52C with a CASP2 inhibitor could induce cell death in resistant cells and ACP52CGK‐α3, a CASP2 inhibiting peptide‐conjugated ACP52CGK, can reduce the cancer burden even in ACP52C‐resistant cancers in a CDX mouse model. These findings underscore the potential for future efficacy tests in human as a broad‐spectrum anticancer drug.

## 4 Experimental Section

### Human Specimens

All experiments involving human materials were conducted with approval from the Institutional Review Board of the Hanyang University Hospital (IRB file no. HYI‐17‐182‐2 and HYI‐17‐182‐6), Korea Institute of Radiological and Medical Sciences (IRB file no. KIRAMS 2019‐06‐001‐002), and Asan Bio‐Resource Center (IRB file no. AMC 2018‐0152). The research protocol was approved by the Ethics Committee, and the entire experimental procedure was carried out in accordance with the institutional guidelines. Both tumor and normal tissues were subjected to histopathological assessment, and each diagnosis was confirmed by pathologists.

### Primary and Organoid Culture of Human Cancer Cells

For primary cell culture, tumor tissue samples obtained from patients undergoing surgery were finely minced, washed with phosphate buffered saline (PBS) containing penicillin/streptomycin, and then treated with trypsin solution at 37 °C for up to 20 min. The resulting cell suspension was filtered through a strainer (BD Falcon, 352235). The filtered cells were maintained in EpiX media, which consists of keratinocyte serum free medium (KSFM; Gibco, 17005042) supplemented with 50 µg mL^−1^ bovine pituitary extract (Gibco, 13028014), 5 ng mL^−1^ human epidermal growth factor (EGF; Thermo Fisher, PHG0311L), 1 µm TGF beta inhibitor (A83‐01; Sigma–Aldrich, SML0788), 5 µm ROCK inhibitor (Y27632; Sigma–Aldrich, Y0503), and 3 µm isoproterenol (Sigma–Aldrich, I6504). The culture medium was refreshed daily. For organoid culture, portions of human tissue samples were separated and transported to the laboratory on ice within 1 h of removal from the patients in cold Hank's balanced salt solution (HBSS) with antibiotics. The samples were washed three times with cold HBSS with antibiotics and then incubated with a mixture containing 0.001% DNase (Sigma–Aldrich, 04716278001), 1 mg mL^−1^ collagenase/dispase (Roche, 10269638001), 200 U mL^−1^ penicillin, 200 mg mL^−1^ streptomycin, and 0.5 mg mL^−1^ amphotericin B (Sigma–Aldrich, A2942) in DMEM/F12 medium (Lonza, BE04‐687F/U1) at 37 °C for 2 h with intermittent agitation. After incubation, the cell suspensions were triturated by pipetting and passed through 70‐µm cell strainers (BD Falcon, 352350). The strained cells were centrifuged at 250 g for 3 min, and the pellet was resuspended in 50 µL serum‐free MBM (DMEM/F12) supplemented with 20 ng mL^−1^ of bFGF (Thermo Fisher, 13256‐029), 50 ng mL^−1^ human EGF, N2 (Thermo Fisher, 17502001), B27 (Thermo Fisher, 17504044), 10 µm Y27632, and 1% penicillin/streptomycin (Thermo Fisher, 15140122). Matrigel (Corning, 356231) was added to establish organoids, and the cell suspension was allowed to solidify on pre‐warmed six‐well culture plates at 37 °C for 10 min. After gelation, 3 mL MBM was added to the well, and the medium was changed every 4 days. Organoids were passaged after 1–3 weeks. For passaging, a solidified matrigel drop containing the organoids was harvested using cold DPBS and then centrifuged at 250 g for 3 min at 4 °C. The pellets were washed with cold PBS and centrifuged at 250 g for 10 min at 4 °C. The organoids were resuspended in 2 mL TrypLE Express (Thermo Fisher, 12604013) and incubated for 10 min at 37 °C for dissociation. Subsequently, 10 mL DMEM/F12 containing 10% FBS was added, and the cells were centrifuged at 250 g for 3 min. The pellets were washed with PBS and centrifuged at 250 g for 3 min. Finally, the pellets were resuspended in MBM + Matrigel (1:2) and reseeded at 1 × 10^4^ cells per 50 µL drop to allow the formation of new organoids.

### Cell Lines and Cell Culture

Information of the cell lines is listed in Table S1 (Supporting Information), including their origin and culture conditions. All cell lines were cultured at 37 °C, 5% CO_2_ incubator. MDA‐MB‐231 (LM1), HepG2, Hep3B, and HCT116 cell lines were authenticated with short tandem repeat markers based on ATCC information. All cell lines tested negative for mycoplasma contamination. Transient downregulation of the gene of interest was conducted by siRNA transfection using Lipofectamine 2000 (Thermo Fisher, 11668019) according to the manufacturer's instructions. Information about siRNAs is provided in Table S2 (Supporting Information). Stable cell lines were established by transfecting cells using Effectene reagent (Qiagen, 301425), followed by selection of cell clones in the presence of 1 µg mL^−1^ puromycin (Sigma–Aldrich, P8833). The establishment of cell lines was confirmed by WB or RT‐qPCR. For doxycycline‐inducible gene expression regulation, cell lines were incubated in the presence of 1 µg mL^−1^ doxycycline (Sigma–Aldrich, D9891) for 2–3 days. To promote sphere formation of the cell lines, each well of the tissue culture dish was coated with 10% poly 2‐hydroxyethyl methacrylate (polyHEMA). An appropriate number of cells per well were seeded in serum‐free DMEM/F12 (HyClone, SH30023.01) supplemented with B27, 20 ng mL^−1^ bFGF, and 20 ng mL^−1^ EGF. For erythroid terminal differentiation experiments, differentiation was assessed by hemoglobin staining using a 0.2% (w/v) tetramethylbenzidine solution (Sigma–Aldrich, T0440) in 0.6% hydrogen peroxide, 3% acetic acid for 10 min in dark condition. Benzidine‐positive or negative cell numbers were counted under the inverted microscope BX50 (Olympus) at 200 × magnification. To synchronize cells in the G1 phase, cells were incubated in a medium containing thymidine (2 mm) for 19 h, released in fresh medium for 10 h, and then incubated in a thymidine medium (2 mm) for 19 h. To synchronize cells in the G2/M phase, cells were incubated in a medium containing thymidine (2 mm) for 19 h and incubated in a medium containing nocodazole (100 ng mL^−1^) for 16 h. After each synchronization, cells were fixed at 4‐h intervals for 24 h with ACP52C treatment, and the cell cycle was analyzed by Propidium Iodide (PI) staining or intracellular protein was analyzed by immunofluorescence. Cisplatin‐resistant variants of each cancer cell line (Hep3B, HCT116, and MDA‐MB‐231) were derived from each original parental cell line by continuous exposure to cisplatin (Selleckchem, S1166). Initially, each cisplatin‐resistant cell line was treated with GI_50_ concentration of cisplatin for 72 h. The media was removed, and cells were allowed to recover for an additional 96 h. This development period was repeated four times for one round, after which GI_50_ concentrations were re‐assessed in each resistant cell line. Cells were then continuously maintained in the presence of cisplatin at these new GI_50_ concentrations. Six rounds were performed for each cell line to establish the final cisplatin‐resistant cell line.

### Cell Viability Test

Various cell lines were treated with peptides, either alone or in combination with the CASP2 inhibitor (Ac‐VDVAD‐CHO; Enzo ALX‐260‐058‐M001) at various concentrations for 4 days. Cell viability was assessed on day 2 or day 4 using MTT (3‐(4,5‐dimethylthiazol‐2‐yl)‐2,5‐diphenyltetrazolium bromide; Sigma–Aldrich, M2128) assay. In brief, fresh cells were seeded in flat‐bottomed 96‐well tissue culture plates at appropriate concentrations for each cell line per well using complete culture medium. The cells were incubated in 100 µL of culture medium containing various concentration of chemicals or peptides at 37 °C for 0 to 96 h. A solution of MTT (5 mg mL^−1^) was added to the culture medium, and the cells were further incubated at 37 °C for an additional 3 h. The MTT medium was then removed, and the formazan crystals were dissolved in DMSO (100 µL per well). The formazan concentration was measured spectrophotometrically at 490 nm using a Varioskan Flash Spectral Scanning Multimode Reader (Thermo Scientific Korea). Nonlinear regression curves for growth inhibition and GI_50_ values for each cell line were estimated from independent replicates using the Prism (version 6) software (GraphPad Software Inc.)

### Peptides and Drugs

The peptides listed in Table S3 (Supporting Information) were synthesized by either ANYGEN (Kwangju, Korea) or PepMic (Suzhou, China) and achieved purity of over 95%. These peptides were synthesized using the Solid‐phase method based on the Fmoc strategy, using an Applied Biosystems 431A peptide synthesizer on a 0.25‐mmol scale. To enhance the stability of ACP52C, saturated C16 palmitic acid, DHA, and E‐pal were utilized. These fatty acids were linked to the lysine (K) residue at the N‐terminal of ACP52C or within the ACP52 and iRGD region through a covalent bond formed between the εK NH3+ and αE or γE COO‐ groups. After synthesis, the final product was prepared through lyophilization and appeared as a white amorphous powder for both ACP52C and its derivatives. To confirm their quality, the purity and molecular weight of the final product were evaluated using amino acid analysis, analytical RP‐HPLC, and MALDI‐MS. All synthesized peptides exhibited solubility in water or saline, displaying no detrimental effect on cellular activity. FQI1, Sorafenib, and cisplatin were purchased from Sigma–Aldrich (SML0413, SML2653) or Selleckchem (S1166), respectively.

### Plasmid Construction

For prokaryotic expression and purification of the GST‐tagged CP2c protein (CP2c wildtype and Pep #5‐2 binding region point mutants; H312A, E315A, D322A, E332A, W336A, H338A, R339A, R341A, R347A, D356A, K359A, R362A, D363A, D364A, D373A, R376A, A380F, K382A, R384A, R387A, and R389A), the site directed mutagenesis method was used. The oligonucleotides used for site‐directed mutagenesis are listed in Table S2 (Supporting Information). For eukaryotic expression of CP2c wildtype and point mutants (E332A, H338A, R339A, R341A, and R347A), each construct of CP2c wildtype and point mutant constructs were subcloned into the pEF1α‐3XFB vector using BamHI and XbaI. Doxycycline‐inducible cell lines were generated using the pDUAL‐Tet‐I/O vector (Patent, KR 10‐0952659‐0000) to express shRNA or the full‐length open reading frame of target genes. Full‐length cDNA was amplified by PCR from previously constructed vectors or complementary DNA of the MDA‐MB‐231 (LM1) cell line using Platinum Taq DNA polymerase high fidelity (Thermo Fisher, 10966‐018) with specific primers listed in Table S2 (Supporting Information). The PCR products were digested by MluI and NdeI or PmeI and SpeI and then ligated into a pre‐digested pDUAL‐tet I/O plasmid. Oligonucleotides containing the shRNA sequence with BglII and HindIII restriction enzyme sites that were compatible with cloning into pSuper puro vector were designed (Table S2, Supporting Information). Annealed double‐strand oligonucleotides in annealing buffer (100 mm NaCl and 50 mm HEPES, pH 7.4) were ligated into pre‐digested pSuper puro plasmid and subcloned into pDUAL‐tet I/O plasmid using EcoRI and ClaI.

### Bacterial Protein Purification

GST‐fused proteins (GST‐CP2c wildtype and point mutants) in the pGEX‐5×1 vector were expressed in BL21(DE3) star pLysS strain (Promega, L1195) and purified as described previously.^[^ ^7a^ ^]^ Bacteria were grown in LB broth containing 100 µg mL^−1^ of ampicillin until optical density reached ≈0.4–0.6 at 600 nm. Subsequently, 0.4 mm of IPTG was applied for ≈2 h at 37 °C to induce protein expression, with the expression time and temperature were optimized for individual constructs. Cultures were centrifuged at 5000 rpm for 15 min at 4 °C and lysed in 1 × 10^7^ cell per 25 µL lysis buffer (50 mm Tris‐HCl, pH 7.4, 150 mm NaCl, 1 mm EDTA, 0.1% triton X‐100, and 1 mm PMSF) with freshly added 1 mm DTT and a protease inhibitor cocktail (Sigma–Aldrich, P8340) for 15 min at ice. The lysates were sonicated twice with four pulses and placed on ice for 10 seconds. After adding triton X‐100 (final 1%), the lysates were centrifuged at 12 000 rpm for 20 min at 4 °C. The resulting lysates were applied to 100 µL bed volume of glutathione Sepharose beads and rotated at 4 °C overnight. The beads were washed three times with 1 mL PBS, and GST‐fused proteins were eluted with glutathione elution buffer (10 mm reduced glutathione in 50 mm Tris‐HCl, pH 7.5). Purified GST‐fused proteins were quantified using the Bradford assay, and the same quantity of proteins was used for point mutation analysis.

### Yeast Two‐Hybrid Screening

Yeast two‐hybrid screening was performed using the MATCHMAKER system (Takara, 630489). The interaction was assessed for β‐galactosidase activity through filter‐lift experiments^[^ ^42^ ^]^ and then quantified using o‐nitrophenyl‐D‐galactopyranoside (ONPG) assays.

### Cell Cycle Analysis

Cells were washed with pre‐chilled PBS, fixed in 70% ethanol for 30 min at ice, and stored overnight at −20 °C. Before analysis, the fixed cells were treated with RNase A (20 µg mL^−1^) and stained with PI staining solution at room temperature for 20 min in dark condition. The samples were immediately analyzed using a BD FACS Canto II using BD FACSDiva software (BD Biosciences). Cell fractions in sub‐G1, G0/G1, S, G2/M, and polyploidy states were quantified in histograms using FlowJo software (BD Biosciences).

### Colony Forming Assay

Cells (5000 cells per well) were seeded in 12 well plates and incubated at 37 °C, 5% CO_2_ for colony formation. After 14 days, the colonies were fixed and stained using the Diff Quick Stain Kit (Sysmex Corporation, #38721). The plate was photographed under × 40 magnification, and the colony areas in the images were quantified using Image J (version 1.51) software for statistical analysis.

### Electron Microscopy

Cells were fixed in 2.5% glutaraldehyde in 0.1 m phosphate buffer (pH 7.4) for 1 h at 4 °C. After washing with phosphate buffer, the samples were postfixed with a solution containing 1% osmium tetroxide and 1.5% potassium ferrocyanide in 0.1 m phosphate buffer for 1 h on ice under the dark condition. Subsequently, the samples were washed again with phosphate buffer. The samples were then dehydrated in an ethanol series and embedded in Epon 812. Polymerization was conducted using pure resin at 70 °C for 2 days. Ultrathin sections (70 nm) were obtained using an ultramicrotome (UltraCut‐UCT, Leica) and collected on Formvar‐coated copper grids. After staining with uranyl acetate and lead citrate, the sections were examined using a Bio‐HVEM system (JEM‐1400Plus at 120 kV and JEM‐1000BEF at 1000 kV).

### Fluorescence Microscopy

For the cell death analysis, ACP52C (2 µm)‐treated MDA‐MB‐231 cell line was stained using eBioscience Annexin V apoptosis detection kit FITC (Invitrogen, 88–8005) according to the manufacturer's recommendation. The stained cells were attached to a slide glass using a Cytospin and analyzed via confocal laser‐scanning microscopy (Nikon C2si). To distinguish between live and dead cells, the nuclear size of the PI‐stained fixed sample with acetone was used as a control. For the DNA damage analysis, cells were grown on coverslips in a 24‐well plate, with or without ACP52C (2 µm) treatment for 12 h. Coverslips were washed with cold PBS and fixed with acetone for 10 min at room temperature. Cells were rinsed with PBS and incubated with 0.005 U µL^−1^ DNase I (Thermo Fisher, EN0525) at 37 °C for 5 min. The digested DNA ends were labeled with 200 µm BrdU using terminal deoxynucleotidyl transferase (CHEMICON, 90418) for 2 h. After blocking with PBS containing 3% horse serum (Gibco, 16050–122) at room temperature for 1 h, cells were incubated with the indicated primary antibodies (BrdU and γH2AX) in PBS containing 1% bovine serum albumin (BSA) solution at 4 °C overnight, rinsed with PBS, and treated with the corresponding FITC‐ or Cy3‐ conjugated secondary antibodies in PBS containing 1% BSA solution at room temperature for 1 h. For the time‐dependent subcellular localization analysis, cells were grown on coverslips in a 24‐well plate. After Cy5‐ACP52C (10 µm) or Cy5‐ACP52CGK (10 µm) was treated to the culture medium for 30 min, the culture medium was replaced with normal medium. At different time points (0.5, 1, 4, 8, or 16 h), coverslips were washed in cold PBS and fixed with acetone for 10 min at room temperature. After blocking with PBS containing 3% horse serum (Gibco, 16050–122) at room temperature for 1 h, cells were incubated with CP2c antibody in PBS containing 1% BSA solution at 4 °C overnight, rinsed with PBS, and treated with the FITC‐conjugated secondary antibodies in PBS containing 1% BSA solution at room temperature for 1 h. Antibody‐labeled cells were stained with 4’,6‐diamidino‐2‐phenylindole (DAPI) for 5 min, washed in PBS, and then mounted using mounting solution (Vectashield, H‐1200‐10). All images were obtained via confocal laser‐scanning microscopy and analyzed using Image J (version 1.51) software.

### Single Nucleotide Polymorphism (SNP) Identification

To detect cell‐specific *MDM2* SNPs, genomic DNA was extracted from cell lines (MDA‐MB‐231, PC9, and A549) using a phenol chloroform extraction method. DNA fragments containing SNP candidates were amplified by PCR using specific primers (listed in Table S2, Supporting Information) and Platinum Taq DNA polymerase high fidelity. The PCR products obtained from each cell line were cloned into pGEMT easy vector (Promega, A1360), and at least 10 clones were subjected to DNA sequencing.

### RNA Extraction and Reverse Transcription and Quantitative PCR (RT‐qPCR)

To quantify mRNA expression of the gene of interest, cells were harvested, and total cellular RNA was extracted using Qiazol reagent (Qiagen, 79306). Purified RNA was dissolved in Diethyl pyrocarbonate (DEPC) water. Reverse transcription was performed using a High‐Capacity cDNA reverse transcription kit (Toyobo, FSQ‐201) in the presence of 400 ng of total RNA and 10 pmol random hexamer. PCR was performed using rTaq Plus 5X PCR master mix (Elpis‐Biotech, EBT‐1319) and the SYBR green Master Mix Kit. Amplifications were performed using a Light cycler 1.5 real‐time PCR system.^[^ ^43^ ^]^ The relative mRNA expression levels were determined using the 2(−ΔCt) method, with GAPDH transcript levels serving as an internal control. Errors were calculated from at least two independent experiments. Oligonucleotide primers used in RT–qPCR are listed in Table S2 (Supporting Information).

### RNA Sequencing and Differential Expressed Gene Analysis

Total cellular RNA was extracted using Qiazol reagent. The RNA quality was examined through spectrophotometry, agarose gel electrophoresis (calculating the 18S and 28S rRNA ratio), and an Agilent Technologies 2100 Bioanalyzer (ensuring a RIN value greater than 7). The library was prepared using the RNA seq3’ mRNA‐Seq Library Prep Kit FWD. The constructed libraries were subjected to 150‐bp paired end sequencing using an Illumina NextSeq 500 sequencer at Insilicogen. All procedures were performed following the manufacturer's instructions. Tophat 2 and cufflinks were used for reads mapping and expected read counts, respectively. DEGs were analyzed using an edgeR program and functional annotation of genes were achieved using a DAVID and GSEA programs.

### Chromatin Immunoprecipitation‐Quantitative PCR (ChIP‐qPCR)

Harvested cells (1 × 10^7^) were cross‐linked by rotating with 1% formaldehyde (Sigma–Aldrich, 252549) in PBS for 10 min at room temperature. Crosslinking was quenched by rotating with 125 mm glycine (Sigma–Aldrich, G4392) in PBS for 5 min at room temperature. Cells were rinsed twice with ice‐cold PBS and lysed with 250 µL of the lysis buffer (10 mm Tris–HCl, pH 8.0, 10 mm NaCl, 100 mm CaCl_2_, and 0.1% NP‐40). Genomic DNA was decomposed by enzyme digestion for 30 min at 37 °C using 10 U µL^−1^ Micrococcus Nuclease (Sigma–Aldrich, N3755) and sonication for 4 times of 10 second pulse on ice using a Sonicator (Hielscher, UP200H) to generate DNA fragments ≈ 200–300 bp in length. After centrifugation at 13 000 rpm for 10 min at 4 °C, the supernatant was pre‐cleared with 50 µL Protein A/G agarose bead (Pierce, 20421). The pre‐cleared chromatin extracts were incubated overnight at 4 °C with 100 µL Protein A/G agarose beads that were pre‐incubated with 3 µg of the appropriate ChIP‐grade antibodies or IgG (listed in Table S4, Supporting Information) for at least 3 h. The beads were washed twice with 500 µL ChIP washing buffer 1 (20 mm Tris–HCl, pH 8.0, 150 mm NaCl, 2 mm EDTA, 0.1% SDS, and 1% Triton X‐100), once with 500 µL ChIP washing buffer 2 (10 mm Tris–HCl, pH 8.0, 250 mm LiCl, 1 mm EDTA, 1% SDS, and 1% NP‐40), and finally twice with 500 µL TE (10 mm Tris pH 8.0 and 1 mm EDTA). The complex was eluted by rotating with 200 µL fresh‐prepared elution buffer (100 mm NaHCO_3_ and 1% SDS) for 30 min at room temperature. Reverse cross‐linking was carried out by adding 250 mm NaCl and then incubating overnight at 65 °C. After treating with RNase A (0.2 mg mL^−1^ final) and proteinase K (0.2 mg mL^−1^ final) for 2 h at 37 °C, DNA was purified by phenol/chloroform extraction and ethanol precipitation. The pellets were dissolved in 100 µL TE buffer for qPCR. qPCR assays were performed using SYBR green (TaKaRa, RR420A) with the specific primers listed in Table S2 (Supporting Information). The data were normalized to the input DNA, and enrichment was calculated by fold excess over ChIP performed with specific IgG as a background signal. All assays were done in duplicate.

### Western Blot (WB)

Cell extracts were prepared using lysis buffer (50 mm Tris‐HCl, pH 7.4, 150 mm NaCl, 1 mm EDTA, 0.1% triton X‐100, and 1 mm PMSF). The cell extracts were separated by SDS‐PAGE and electro‐blotted onto polyvinylidene difluoride (PVDF) membranes (GE healthcare, 10600069). For slot blotting, the cell extracts were blotted onto nitrocellulose (NC) membranes (GE healthcare, 10484059) using a Bio‐Dot (Bio‐Rad, 1706542). Typically, membranes were blocked with 5% non‐fat dry milk in a solution of 0.1% Tween 20, and with 5% BSA in case of biotin blotting. Membranes were incubated overnight at 4 °C with appropriate dilutions of the primary antibodies (listed in Table S4, Supporting Information) after blocking. The blots were incubated for 1 h at room temperature with the respective HRP‐conjugated secondary antibodies (listed in Table S4, Supporting Information). Polyclonal ACTB antibody was used as a loading control for WB. Proteins were visualized by chemiluminescence using an ECL system (GE healthcare, RPN2106). Relative amounts of proteins were quantified using Image J (ver. 1.51) program.

### Co‐Immunoprecipitation (co‐IP) Assay

Cells were lysed with lysis buffer (50 mm Tris‐HCl, pH 7.4, 150 mm NaCl, 1 mm EDTA, 0.1% triton X‐100, and 1 mm PMSF) with freshly added 1 mm DTT and a protease inhibitor cocktail. Input samples (10% of whole lysate) were saved from each lysate for WB analysis. For immunoprecipitation, precleared extracts were incubated overnight at 4 °C with 10 µL Protein A/G agarose beads, pre‐incubated with 2 µg of the primary antibodies (Table S4, Supporting Information). The immune complexes were washed 3 times with lysis buffer, and bound proteins were eluted with 2X bed volume of 0.2 m glycine buffer, followed by neutralization with an equal volume of 1 m Tris‐HCl, pH 8.0. For Flag tag immunoprecipitation, precleared extracts were incubated with 2 µL of Flag‐M2 beads (Sigma–Aldrich, A2220) by rotating overnight at 4 °C. The immune complexes were washed 3 times with lysis buffer, bound proteins were eluted with 100 µg mL^−1^ Flag peptide (Sigma–Aldrich, F4799). Precipitated proteins were analyzed using western blot.

### DNA Immunoprecipitation (DNA‐IP) Assay

DNA‐IP assay was performed essentially as described previously.^[^ ^14^ ^]^ Each of the oligonucleotides was mixed and annealed in the TEN buffer. For the radio‐labeling of the DNA probe, the annealed DNA was incubated with a mixture of dATP, dGTP, dTTP, [α^32^P]‐dCTP, and Klenow enzyme in reaction buffer (5 mm NaCl, 1 mm Tris HCl, 1 mm MgCl_2_, and 0.1 mm DTT) for 30 min at 25 °C, and stopped by incubation with EDTA (10 mm final) for 20 min at 75 °C. The radio‐labeled DNA probe was purified by applying the reaction mixture to ProbeQuant G‐50 micro columns (GE healthcare, GE28‐9034‐08). Whole extracts prepared from transfecting 293T cells or purified GST fused CP2c proteins (wildtype and point mutants) were incubated with a [α^32^P]‐labeled DNA probe in binding buffer (4% glycerol, 10 mm Tris‐HCl, pH 7.4, 1 mm DTT, 1 mm EDTA, and 0.1% NP‐40) for 15 min at room temperature. Then 0–3 µm of peptides (Pep #5, Pep #8, Pep #5‐1, Pep #5‐2, and Pep #5‐2C) were added to the DNA‐protein mixture, and the mixtures were incubated for 15 min at room temperature (peptide sequences are listed in Table S3, Supporting Information). For IP, precleared extracts were incubated overnight at 4 °C with 1 µg of the following primary antibodies (CP2c, CP2b, and PIAS1). Subsequently, 50 µL Protein A/G agarose bead were added to the mixture and incubated for an additional 3 h at 4 °C. The precipitated‐complexes were washed three times with wash buffer (50 mm tris‐HCl, pH7.4, 150 mm NaCl, 1 mm EDTA, and 1 mm PMSF). The labeled DNA probes were eluted from the precipitated DNA‐protein complex with elution buffer (50 mm Tris‐HCl, pH7.4, 10 mm EDTA, and 1% SDS) for 1 h at 65 °C. The radioactivity of the eluted probe was measured by scintillation counting.

### Pull Down Assay

Biotinylated‐DNA fragments or ‐peptides and GST‐fused CP2c, whole‐ or nuclear‐ extracts of MDA‐MB‐231 cells were used for the analyze the interaction of peptide or DNA with CP2c. Nuclear extracts were extracted by the method described elsewhere.^[^ ^14^ ^]^ Various samples prepared to analyze these interactions and were incubated overnight at 4 °C with the glutathione‐Sepharose beads (GeneScript, L00206) or Streptavidin‐Sepharose beads (Invitrogen 15942‐050). Pull‐down samples were washed three times with lysis buffer. For WB, proteins were eluted by boiling in 2X SDS‐PAGE sample loading buffer. For mass analysis, proteins were eluted by trypsinization using sequencing grade modified trypsin (Promega, V5111) overnight at 37 °C. For the competition binding assay, GST‐fused CP2c and Pep #5‐2A were incubated with 0, 10, and 50 µm of CP2c consensus peptide (CP2c 1P, 2P, 3P, and 4P) and the [α^32^P]‐radiolabeled DNA probe in the binding buffer (4% glycerol, 10 mm Tris‐HCl, pH 7.4, 1 mm DTT, 1 mm EDTA, 0.1% NP‐40) for 30 min at room temperature. Then, CP2c antibody and protein A/G beads were added to the mixture. The [α^32^P]‐radiolabeled DNA probe from the precipitated DNA‐protein complex was eluted with elution buffer (50 mm Tris‐HCl, pH7.4, 10 mm EDTA, and 1% SDS) for 1 h at 65 °C. The radioactivity of the eluted probe was measured by scintillation counting. The peptides, used in this paper are listed in Table S3 (Supporting Information).

### Caspase‐2 Activity Assay

Caspase‐2 assay kit (Abcam, ab39830) was used for analyzing the activity of CASP2 in different cell lines. 100 µg of total cell lysates were incubated with 200 µm of substrate (Ac‐VDVAD‐pNa) for 2 h at 37 °C. The relative enzyme activity was measured spectrophotometrically at 400 nm using a Varioskan Flash Spectral Scanning Multimode Reader.

### DSP [Dithiobis(Succinimidyl Propionate)] Crosslinking

To determine DNA‐free CP2c containing complexes, live cells were washed twice with the 2.5 mm sodium phosphate buffer, pH 7.4, crosslinked using 2 mm DSP in PBS for 3 h on ice, and crosslinking was terminated with 50 mm Tris‐HCl, pH 7.4, for 15 min at room temperature. After intracellular DSP crosslinking, cells were lysed in non‐Tris based lysis buffer (50 mm HEPES, 150 mm NaCl, 1 mm EDTA, 1% NP40, and 1 mm PMSF) for 20 min at 4 °C. To determine CP2c complexes and Pep #5‐CP2c complex, transfected mammalian cells and GST‐fused CP2c protein (wildtype and mutants) expressing bacterial cells were harvested and lysed in non‐Tris based lysis buffer for 20 min at 4 °C. Lysate was incubated with biotin‐labeled probes^[^ ^7b^ ^]^ or biotin‐labeled peptides (listed in Table S3, Supporting Information) and DSP cross‐linker (final 2 mm) in PBS for 30 min at 25 °C, and crosslinking was terminated with 20 mm Tris‐HCl, pH 8.0 at 25 °C. Crosslinked samples were analyzed by pulldown assay or mass spectrometry.

### Mass Spectrometry Analysis

Each sample was alkylated by incubating with iodoacetamide (10 µg µL^−1^ final) for 20 min in the dark and precipitated using 10% trichloroacetic acid. Sequencing grade modified trypsin (0.3 µg) was added to each sample and allowed to incubate overnight at 37 °C. Each sample was analyzed on a Shimadzu Axima MALDI‐TOF mass spectrometer. One microliter of each digested sample in 30% ACN and 50 mm NH_4_HCO_3_ was spotted on a MALDI plate in a sandwich‐style manner with 10 mg mL^−1^ of alpha‐cyano‐4‐hydroxycinnamic acid matrix at a total ratio of matrix and sample (1:1). All MS spectra were acquired in linear mode with an average of 300 profiles per sample and an average power of 90. All peptide molecular weights were identified using DataExplorer (Applied Biosystems) and analyzed using ExPasy and a manual method. Solvent assisted surface distance (SASD) was calculated using J walk software.^[^ ^44^ ^]^ Crosslink profiles with SASD less than 30 Å were visualized using PyXlinkViwer.^[^ ^45^ ^]^

### Tertiary Structure Prediction (MODELLER and I‐TASSER)

Tertiary structures of CP2c were predicted using homology or comparative modeling using MODELLER^[^ ^46^ ^]^ and I‐TASSER.^[^ ^47^ ^]^ To initiate the modeling process, the amino acid sequence spanning position 306 to 396 of the mouse CP2c was obtained in FASTA format from the National Center for Biotechnology Information. The tertiary structure of CP2c was predicted through the I‐TASSER server, utilizing the SAM_PNT domain of human protein FLK21935 (PDB ID: 1WWU) as a template. The I‐TASSER server is an integrated platform for automated prediction of protein structure based on the sequence‐to‐structure approach. For the prediction of the full‐length CP2c structure, a combination of the DNA binding structure of Grhl1 (PDB ID: 5MPH) and the predicted structure of CP2c TD (306 to 396 amino acids) generated by I‐TASSER were employed as templates for MODELLER based homology modeling. All structure models were visualized and analyzed using PyMOL software.

### Crosslink Derived Molecular Docking

HADDOCK was employed to generate structural models for the CP2c TD tetramer and binding models for CP2c TD and Pep #5‐2, utilizing DSP XL‐MS data.^[^ ^48^ ^]^ Default parameters from the HADDOCK server were used for the creation of structural models. However, when constructing the CP2c TD tetramer, non‐crystallographic and C2 symmetric restraints were incorporated. Semi‐flexible residues were automatically identified by HADDOCK, while fully flexible residues were defined to accommodate the DSP XL‐MS data, considering that DSP has a 12 Å spacer arm. One thousand structures were generated for the first iteration (rigid docking), 200 were generated for the second iteration (semi‐flexible docking), and 200 lowest energy structures were refined in water. The selection of the optimal structural model considered C4 symmetric information, experimental findings from the CP2c point mutant study, and the results from the competitor peptide experiments. To analyze the residues lying on the surface of the tetramer structure, the findSurfaceResidues script was used, and the RMSD cutoff was assigned to default.

### In Vivo Tumorigenicity and Anticancer Effect Assays

Animal experiments were conducted in accordance with ethical guidelines, and all protocols were approved by the Institutional Animal Care and Use Committee under Hanyang University (approval numbers, 2014‐0016A, 2018‐0049A, 2018‐0207A, and 2019‐0169A), Chung‐Ang University (approval number, 2015‐00022), Chungbuk University (approval number, CBNUA‐1072‐17‐02), and Konkuk University (approval number, KU17130). To establish xenograft mice models, Hep3B, A431, U343, and PC9 cells were subcutaneously injected into the flank of Balb/c nude mice. For the MDA‐MB‐231 (LM1) cell line, it was implanted into the mammary fat pad of immunodeficient mice (NPG mice). Once tumors were successfully established, the animals were randomly divided into a control (vehicle) group and an experimental (drug) group. When the tumor volume reached 100 cubic millimeters, either saline or an appropriate concentration of drugs was administrated into mice via tail vein every 3 or 5 days for appropriate duration. Tumor volume was calculated using the formula of length × width^2^)/2. To generate an orthotopic liver cancer mice model, 75 mg kg^−1^ (mpk) of DEN was intraperitoneally injected weekly for 3 weeks into C57BL/6 mice, followed by 100 mpk of DEN weekly for the subsequent 3 weeks. After 37 weeks of liver cancer induction, mice received either saline or an appropriate concentration of drugs via the tail vein every 5 days for the designated duration. The in vivo administration amount was determined based on the concentration of ACP52C confirmed at the cellular level, which was 2 µm. Assuming an average mouse weight of 30 grams and considering that ≈5.8% of body weight constitutes blood volume,^[^ ^49^ ^]^ it was calculated that the blood concentration of ACP52C should be ten times the GI_50_ concentration in cell culture conditions. This calculation resulted in the baseline concentration of 2 mpk, and 2 mpk of ACP52C was effective in Hep3B and MDA‐MB‐231 LM1 xenograft models. For ACP52C derivatives, the amount was adjusted to ensure a similar molar quantity of peptide injection, taking their molecular weights into account. For instance, in the case of ACP52CGK, ≈1.67 times the quantity was used compared to ACP52C to achieve an equivalent molar quantity. At the time of sacrifice, major organs and tumors were removed and weighed, and blood was extracted. For histological analysis, major organs and tumors of mice were immediately fixed in 10% formalin neutral buffer solution, dehydrated through a graded series of ethanol treatments, treated with xylene, paraffin embedded, and cut into 4 µm sections. Hematoxylin and eosin (H&E)‐stained tissue sections were analyzed and photographed, and the representative histological images were recorded. Liver nodules in the DEN orthotopic liver cancer model were estimated by measuring the ratio of tumor areas over entire liver tissues in the tissue section using an Image J (version 1.51) software at 40× magnification. Complete blood count (CBC) analysis was conducted using a Hematology analyzer (scil Vet ABC) from the extracted blood.

### In Vivo Toxicity Test

To assess the toxicity of ACP52C, twelve 6‐weeks‐old ICR mice (six males and six females) were divided into three groups, each containing two mice: one group received a saline, another received 100 mg kg^−1^ of ACP52C, and the third received 1000 mg kg^−1^ of ACP52C. For each group, one mouse was intravenously injected once, and the other one was intravenously injected twice with a four‐day interval between injections. At the time of sacrifice, major organs were analyzed by H&E staining. To assess the repeated toxicity of ACP52CGK, twenty‐four 6‐weeks‐old ICR mice (twelve males and twelve females) were used, dividing them into two groups of six mice each: one group received saline, and the other received 100 mg kg^−1^ of ACP52CGK. For each group, mice were intravenously injected with 100 mg kg^−1^ of ACP52CGK daily for 4 weeks. Body weight, water and feed consumption were measured every 3 days after injection. At the time of sacrifice, major organs were removed, and blood was extracted for analysis. Major organs were weighed and analyzed by H&E staining. H&E‐stained tissue sections were analyzed and photographed, and the representative histological images were recorded. From the collected blood samples, CBC analysis was performed using Hematology analyzer, and blood chemistry analysis was conducted using Automated Chemistry Analyzer (FUJIFILM NX700).

### In Vivo Pharmacokinetics Test

A pharmacokinetics study was conducted at WuXiAppTec., Ltd. In this study, male CD‐1 (ICR) mouse was given a single intravenous injection of 5 mg kg^−1^ of ACP52CGK. Blood samples were collected prior to dosing on day 0 and at various time points, including 0.033, 0.083, 0.25, 0.5, 1, 2, 6, 12, 24, and 48 h after dosing. The data collected from these samples were used to calculate pharmacokinetic parameters using the IV‐noncompartmental analysis model 201 in Phoenix WinNonlin version 6.3.

### In Vivo Metabolic Stability Test of Peptides

All animal procedures were performed according to the guidelines of the Animal Care and Use Committee and the Institutional Review Board of Hanyang University. Hep3B cells were subcutaneously injected into the flank of Balb/c nude mice. Once the tumor volume reached 100 cubic millimeters, Cy5‐ACP52C (17 mg kg^−1^) and Cy5‐ACP52CGK (25 mg kg^−1^) were administered into the mice via tail vein injection. This dosage was chosen based on preliminary experiments to facilitate easier observation. Fluorescence intensity was monitored for 24 h using live imaging on the Kodak Image Station 4000MM, with excitation at 465 nm. On the 5th day after peptide injection, the mice were euthanized, and major organs and tumors were histologically dissected and photographed under bright field and red fluorescence conditions.

### Physicochemical Analyses of Peptides

For determining the dimensions and nanoparticle formation of the peptides in solution, DLS analysis was performed using the Scatteroscope I nanoparticle size analyzer (K‐One, SOS1). Saline served as the negative control, and all experiments were conducted at a constant temperature of 25 °C. Each sample was analyzed five times, and the resulting data were graphically presented, incorporating all five profiles obtained from the experiments. The purity and dimer formation of the peptides were analyzed using HPLC and LC‐MS. HPLC analysis was carried out using a Shimadzu HPLC LabSolution equipped with a pump, an autosampler, a column oven, and a UV–vis detector. Chromatographic separation was performed on a Shimadzu C18 analytical column at 35 °C, with an injection volume of 20 µL (0.5 mg mL^−1^). The mobile phases consisted of 0.05% trifluoroacetic acid (TFA) in water (A) and 0.05% TFA in acetonitrile (B). The flow rate was maintained at 1 mL min^−1^, and the gradient program was as follows: 20–80% B over 30 min. Ionization was achieved using electrospray ionization (ESI) in positive mode with selected reaction monitoring (SRM). Mass spectrometry analysis was performed with an ESI source in the positive‐ionization mode.

### Quantification and Statistical Analysis

Prism software (version 6) and SPSS statistics software (version 21.0) were used to analyze differences between groups and determine statistical significance. Data were considered statistically significant when *p* < 0.05. Normality assumptions were assessed for outcomes before statistical tests. Group differences were evaluated through various statistical analyses. For comparisons between two groups, a two‐tailed unpaired Student's *t*‐tests was employed. To compare more than one independent variable, statistical analysis was carried out by ANOVA test. In cases where normality was confirmed, a one‐way analysis of variance test with post hoc Tukey‐Kramer's multiple comparison test was used. When normality was not confirmed, a nonparametric Kruskal–Wallis test with post hoc Dunn's multiple comparison test was applied. Data were presented as means ± standard deviation (SD) for in vitro studies or as means ± standard error (SE) for in vivo studies. In figures, asterisks denote statistical significance (*p < 0.05, **p < 0.01, ***p < 0.001, and ns; non‐significant).

## Conflict of Interest

The authors declare no conflict of interest.

## Author Contributions

S.H.S., M.Y.K., and S.C. contributed equally to this work. Chul G.K. conceived and designed research, with contributions from H.C.K., C.‐O.Y., J.H.C., J.‐W.L., S.‐J.L., and Chan G.K. S.H.S., M.Y.K., S.C., J.S.K., Y.S.L., S. L., Y.J.L., J.Y.L., S.E.L., Y.S.L., D.H.H., E.O., Y.‐B.W., C.‐J.J., M.A.P., J.H.C., H.C.K., B.K., K.T.B., A.R.J., H.‐S.K., D.P., and S.‐J.L. conducted experiments. M.S.C., J. J., D.C., E.J.B., E.‐H.C., S.‐B.K. H.S.P., J.S.B., and S.J.J. provided human patient tissues or human‐originated cells. S.H.S., M.Y.K., H.C.K., D.P., C.‐O.Y., J.‐W.L., S.‐J.L., Chan G.K., and Chul G.K. analyzed and interpreted results. Chul G.K. H.C.K., and V.N.U. supervised the study and wrote the manuscript with input from all authors.

## Supporting information

Supporting Information

## Data Availability

The data that support the findings of this study are available in the supplementary material of this article.
