# Supplementary material for: A Cell‐Penetrant Peptide Disrupting the Transcription Factor CP2c Complexes Induces Cancer‐Specific Synthetic Lethality
Source: Adv Sci (Weinh). 2023 Oct 16;10(33):2305096. doi: 10.1002/advs.202305096 (PMC10667816; doi:10.1002/advs.202305096)
Supplement: Supplementary file 1 — Supporting Information [file ADVS-10-2305096-s001.pdf]

ADVANCED SCIENCE
Open Access

Supporting Information

for *Adv. Sci.*, DOI 10.1002/advs.202305096

A Cell-Penetrant Peptide Disrupting the Transcription Factor CP2c Complexes Induces Cancer-Specific Synthetic Lethality

Seung Han Son, Min Young Kim, Sungwoo Choi, Ji Sook Kim, Yong Sang Lee, Sangwon Lee, Yeon Ju Lee, Jin Youn Lee, Seol Eui Lee, Young Su Lim, Dae Hyun Ha, Eonju Oh, Young-Bin Won, Chang-Jun Ji, Mi Ae Park, Boram Kim, Kyu Tae Byun, Min Sung Chung, Jaemin Jeong, Dongho Choi, Eun Jung Baek, Eung-Ho Cho, Sang-Bum Kim, A. Reum Je, Hee-Seok Kweon, Hyun Sook Park, Dongsun Park, June Sung Bae, Se Jin Jang, Chae-Ok Yun, Ji Hyung Chae, Jin-Won Lee, Su-Jae Lee, Chan Gil Kim, Ho Chul Kang, Vladimir N. Uversky* and Chul Geun Kim**

Supporting Information

A cell-penetrant peptide disrupting the transcription factor CP2c complexes induces cancer-specific synthetic lethality

Seung Han Son[1]†, Min Young Kim[1]†, Sungwoo Choi[1]†, Ji Sook Kim[1,2], Yong Sang Lee[1], Sangwon Lee[1], Yeon Ju Lee[1], Jin Youn Lee[1], Seol Eui Lee[1], Young Su Lim[1], Dae Hyun Ha[1], Eonju Oh[3], Young-Bin Won[1], Chang-Jun Ji[1], Mi Ae Park[1], Boram Kim[4], Kyu Tae Byun[4], Min Sung Chung[5], Jaemin Jeong[5], Dongho Choi[5], Eun Jung Baek[6], Eung-Ho Cho[7], Sang-Bum Kim[7], A Reum Je[8], Hee-Seok Kweon[8], Hyun Sook Park[9], Dongsun Park[10], June Sung Bae[11], Se Jin Jang[11,12,13], Chae-Ok Yun[3], Ji Hyung Chae[1], Jin-Won Lee[1], Su-Jae Lee[1]‡, Chan Gil Kim[4], Ho Chul Kang[1]‡*, Vladimir N. Uversky[14]*, Chul Geun Kim[1,15]*

Correspondence to: hckang@ajou.ac.kr (H.C.K.), vuversky@usf.edu (V.N.U.), and cgkim@hanyang.ac.kr (C.G.K.)

This PDF file includes:

Figure S1 to S27
Table S1 to S4

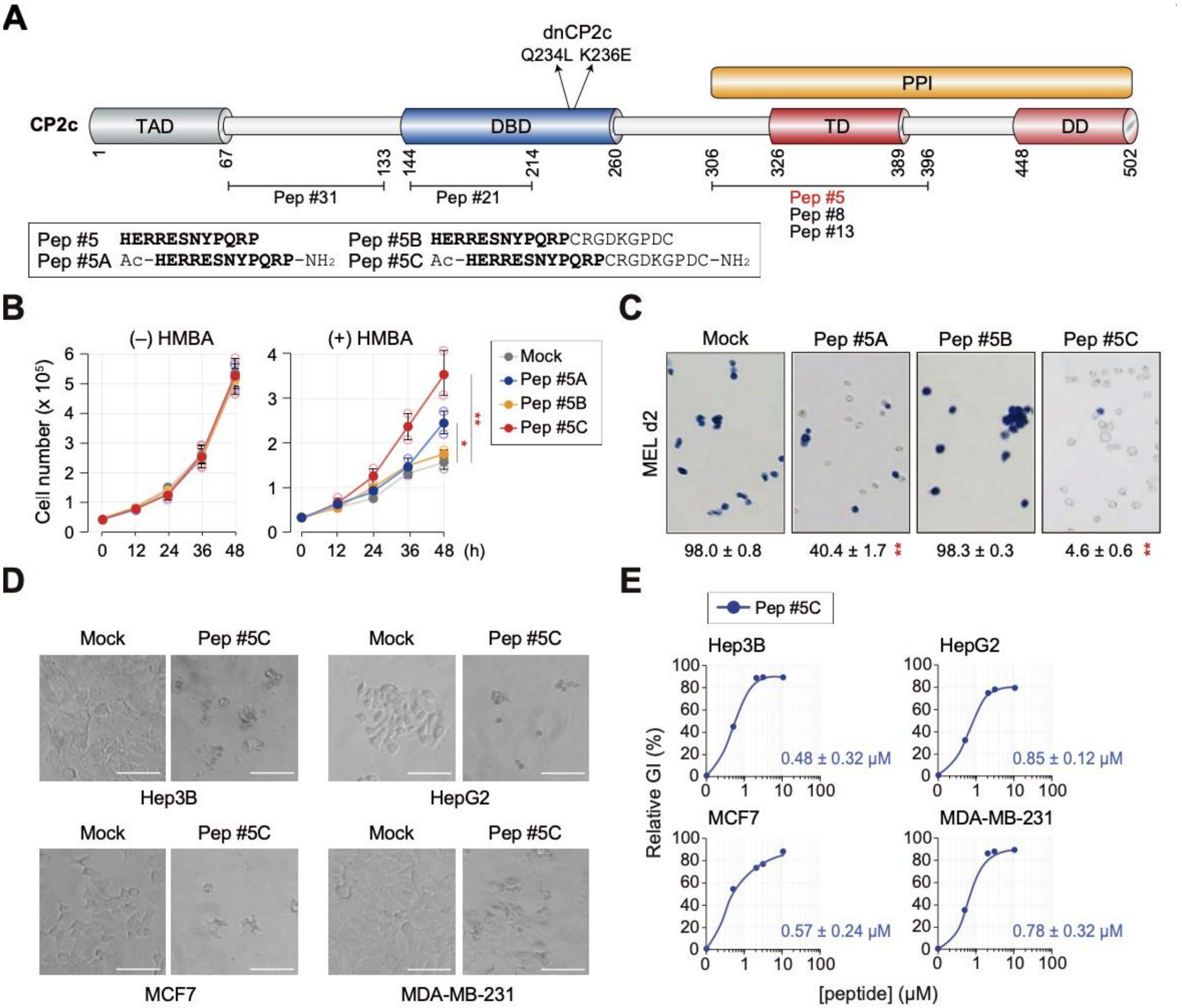

Figure S1. Targeting CP2c with Pep #5 inhibits HMBA-induced MEL cell differentiation and induces cancer cell death

A) Schematic diagram showing the functional domains of the CP2c protein where various peptides bind. Amino acid sequences and N- and C-terminal modifications of Pep #5 and its derivatives are displayed in the lower left box. TAD, transactivation domain; DBD, DNA binding domain; TD, tetramerization domain; DD, dimerization domain; PPI, protein-protein interaction; dnCP2c, dominant negative form of human CP2c (Q234L and K236E). B,C) Pep #5 targets CP2c to inhibit HMBA-induced MEL (iMEL) cell differentiation. B) Cell proliferation assays in uninduced MEL (uMEL) and iMEL cells demonstrates effective inhibition of erythroid differentiation by Pep #5C (10 μM). Data are presented as means ± SD of two independent biological replicates. Two-tailed unpaired Student's *t*-tests; *$P < 0.05$, **$P < 0.01$. C) Functional hemoglobin synthesis analysis using benzidine staining. Functional hemoglobin synthesis analysis was performed using benzidine staining, and the percentages of benzidine-positive stained cells were quantified relative to the total cell count. Representative staining images are provided, and below them, the benzidine-positive percentages are reported as means ± SD from two independent biological replicates. Two-tailed unpaired Student's t-tests, **$P < 0.01$. D,E) Pep #5C induces robust growth inhibition and cell death of cancer cells. Cell photographs captured at 96 h after Pep #5C (10 μM) treatment (D) and growth inhibition profiles with GI_{50} values (E) in the representative cancer cells at 96 h after Pep #5C treatment. GI_{50} values estimated from two independent biological replicates.

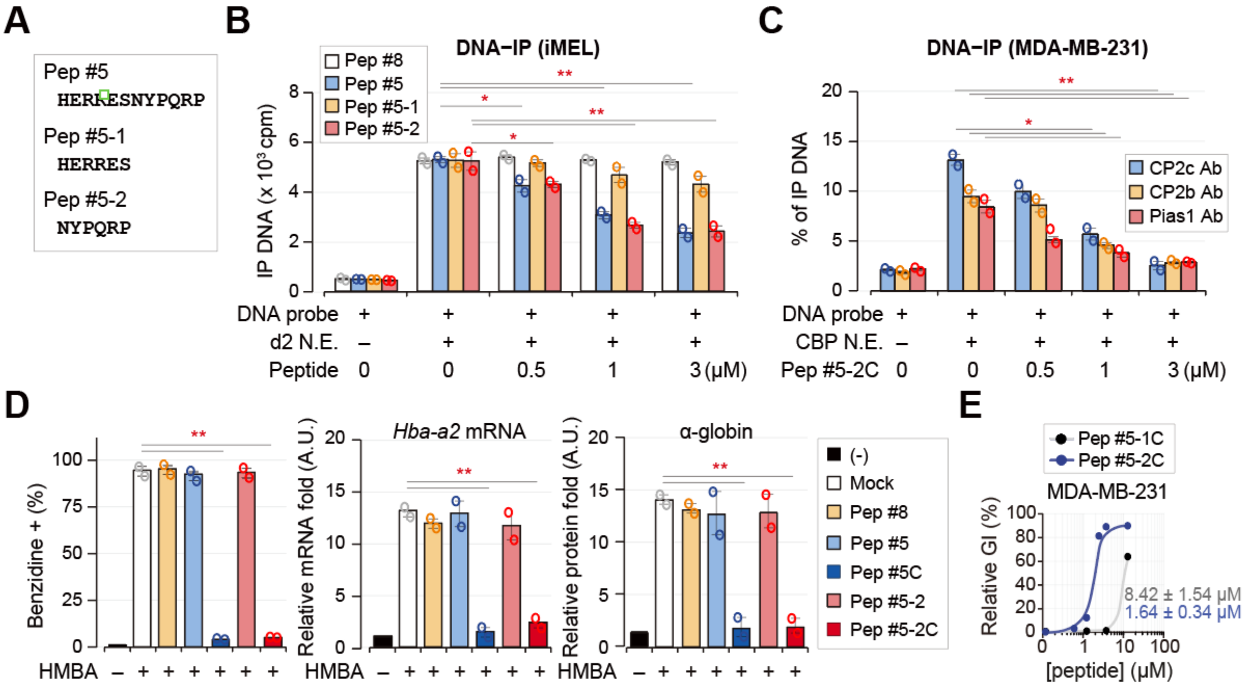

Figure S2. Pep #5-2, a 6-amino acid peptide corresponding to the C-terminal half of Pep #5, exhibits the same activity as Pep #5

A-C) Pep #5-2 inhibits the DNA binding activity of CP2c as does by Pep #5. (A) Amino acid sequences of Pep #5 and its derivatives, Pep #5-1 and Pep #5-2. The isotope labeling (B) or biotin labeling (C) was performed on the wildtype *Hba-a2* promoter which were served as a probe in reactions containing nuclear extracts from iMEL cells (day 2) or MDA-MB-231 cells, respectively. Data are presented as means ± SD of two independent biological replicates. Two-tailed unpaired Student's *t*-tests; $**P < 0.01$; $*P < 0.05$. D) Pep #5-2C (Pep #5-2 linked to a cell penetrating peptide, iRGD) efficiently inhibits functional hemoglobin synthesis (left), and *Hba-a2* globin gene expression at both mRNA (middle) and protein (right) levels in iMEL cells. Fractions of benzidine-positive stained cells were counted in MEL cells (n > 100 cells/group) treated with HMBA for 2 days. *Hba-a2* gene expression at mRNA and protein levels were estimated by RT-qPCR and WB, respectively. Data are presented as means ± SD of two independent biological replicates. Two-tailed unpaired Student's *t*-tests; $**P < 0.01$. E) Growth inhibition profiles of MDA-MB-231 cells at 96 h after treatment of Pep #5-1C or Pep #5-2C. GI_{50} values estimated from two independent biological replicates.

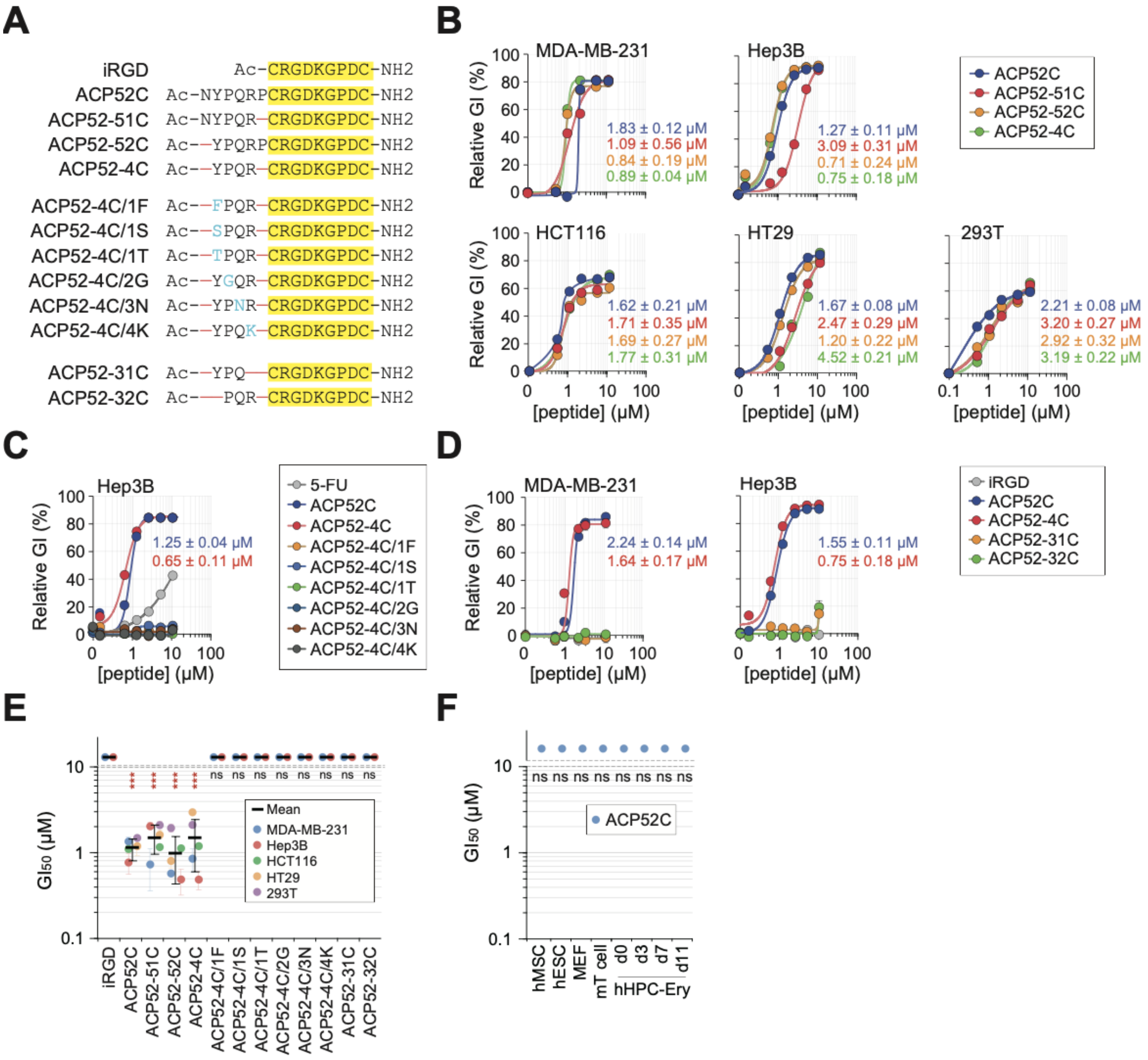

Figure S3. Anticancer effect of Pep #5-2 truncated and substitution mutants

A) Names and peptide sequences of each mutant. Peptides corresponding to each Pep #5-2 truncated or substitution mutant are linked to a cell penetration peptide, iRGD (highlighted with yellow boxes). B-E) Cell growth inhibition profiles and GI_{50} values of each mutant in representative cancer cell lines. GI_{50} values estimated from duplicated data are expressed as means ± SD. Data are presented as means ± SD of two independent biological replicates. Anticancer effect of ACP52C, ACP52-51C, ACP52-52C, and ACP52-4C (B), amino acid substitution mutants of ACP52-4C (C), and ACP52C, ACP52-4C, ACP52-31C, and ACP52-32C (D). Cell growth inhibition was tested in cells using MTT assay over peptide dose at 48 h after peptide treatment, and GI_{50} values estimated from duplicated data are expressed as means ± SD. (E) Summary of GI_{50} values of each mutant in representative cancer cell lines. GI_{50} values estimated from duplicated data are expressed as means ± SD. Two-tailed unpaired Student's *t*-tests, ***$P < 0.001$; ns: non-significant. F) GI_{50} values of ACP52C in various normal human and mouse cells, indicating their resistance to ACP52C. hMSC; human mesenchymal stem cells, hESC; human embryonic stem cells, MEF; mouse embryonic fibroblasts, mT cell; mouse T cells, hHPC-Ery; differentiating erythroid cells in vitro from the human $CD34^+$ hematopoietic progenitor cells. Two-tailed unpaired Student's *t*-tests, ns: non-significant.

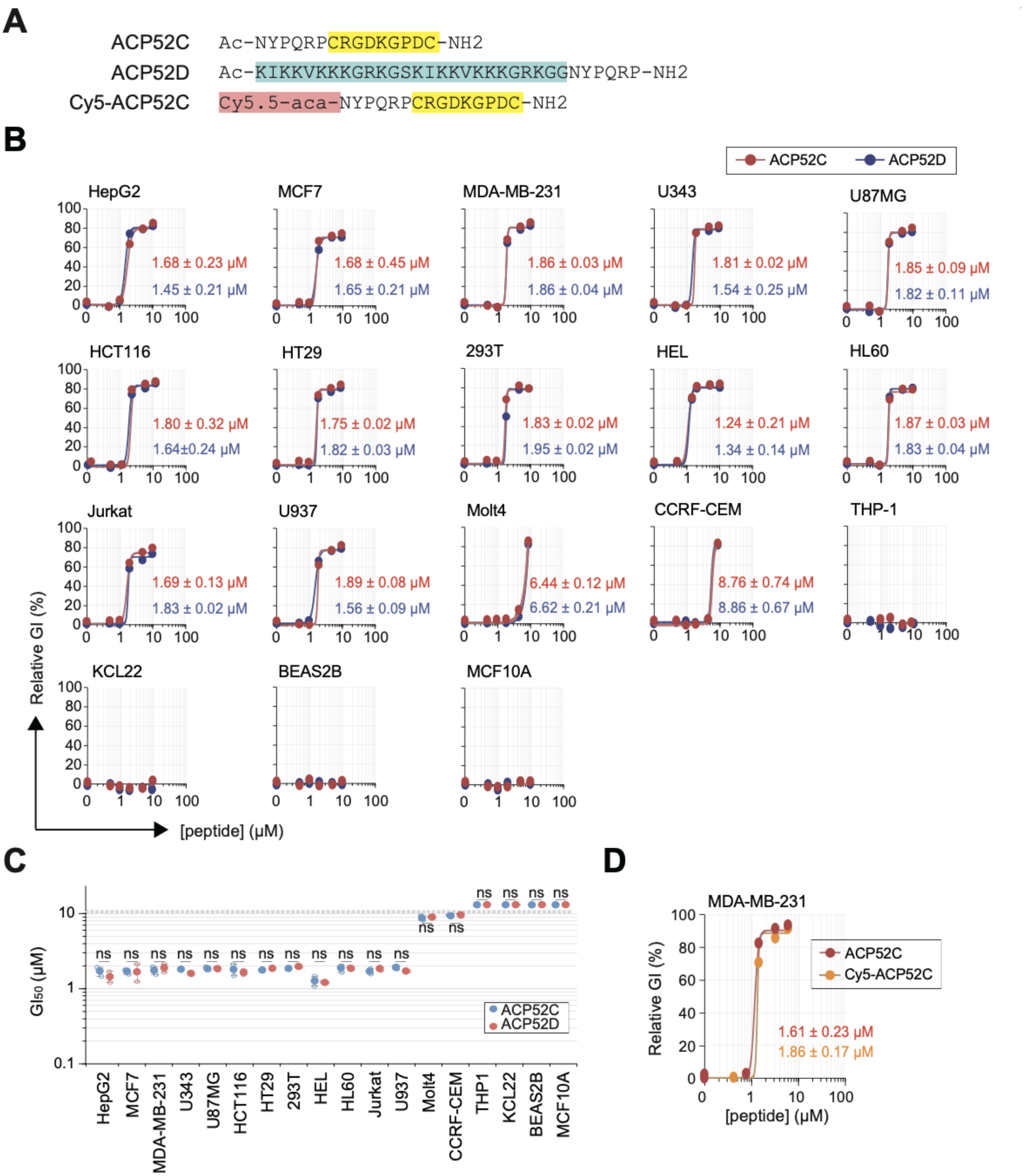

Figure S4. The ACP52 sequence by itself exhibits anticancer activity irrespective of the attached CPPs

A) Peptide sequences of ACP52C, ACP52D, and Cy5-ACP52C, with highlighted boxes of yellow, cyan, and salmon for the iRGD or dNP2 CPP, and Cy5 fluorochrome attached by a linker, respectively. B,C) Cell growth inhibition plots and GI_{50} values of ACP52C and ACP52D in various cancer cell lines over peptide dose (B). The summarized GI_{50} values are presented in (C). GI_{50} values estimated from duplicated data are expressed as means ± SD. Two-tailed unpaired Student's *t*-tests, ns: non-significant. Cell growth was assessed from duplicated data at 96 h after peptide treatment using MTT assay and expressed as means ±

SD. Two-tailed unpaired Student's *t*-tests, ns: non-significant. D) Comparative cell growth inhibition curves and GI_{50} values of ACP52C and Cy5-ACP52C. GI_{50} values estimated from duplicated data are expressed as means ± SD.

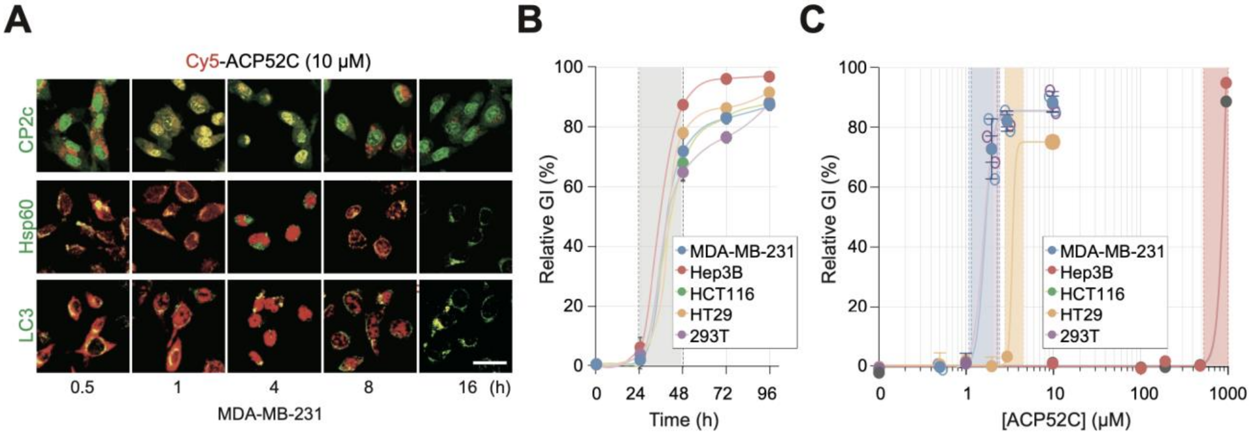

Figure S5. Prognostic characteristics of ACP52C at the cell level

A) Time-dependent subcellular localization of ACP52C. Merged confocal microscopy images demonstrating the colocalization of ACP52C with CP2c, the mitochondrial marker Hsp60, or the lysosomal marker LC3 over time following treatment with Cy5-ACP52C. Cy5-ACP52C (10 μM) was supplemented to the culture medium for 30 min in MDA-MB-231 cells, and IF was performed at different time points after replenishing of fresh medium to observe the subcellular localization of ACP52C. Scale bar represents 10 μm. B,C) ACP52C exerts growth inhibition effects on cells at least 48 h after treatment (B) and within a narrow concentration range (C). Growth inhibition profiles of representative cells from duplicated data are depicted at 24, 48, 72, and 96 h (B) or at 96 h (C) after ACP52C treatment.

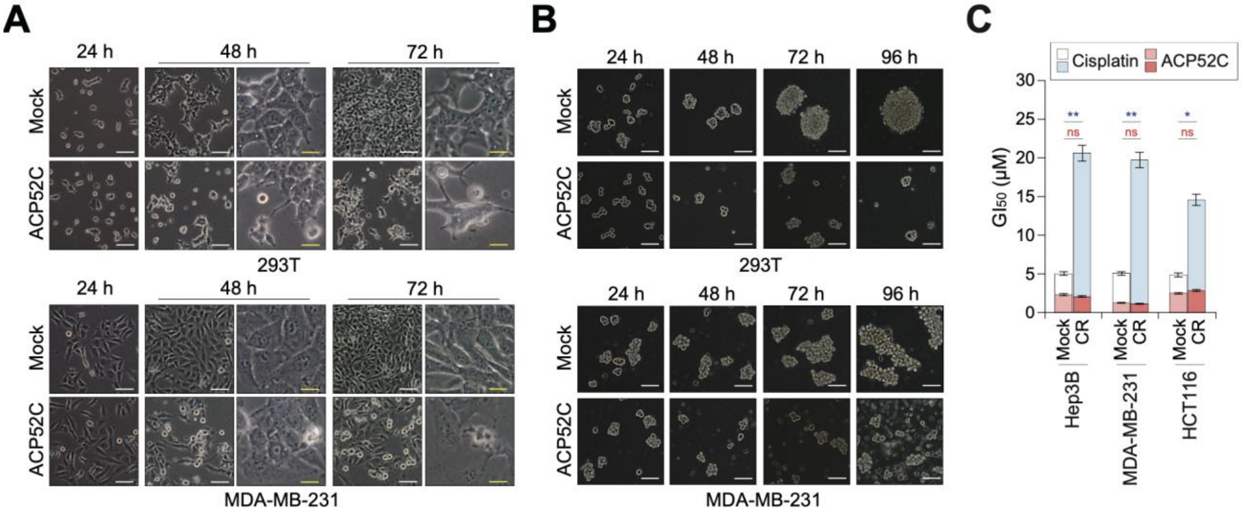

Figure S6. ACP52C drives cancer cell-specific synthetic lethality

A,B) ACP52C exerts its anticancer activity on cancer cells independently of the culture condition, whether it's in monolayer (A) or in mammosphere-forming conditions (B). Photographs depict 293T and MDA-MB-231 cells cultured in either monolayer or mammosphere-forming condition over time after treatment of ACP52C (2 μM). Yellow and white scale bars represent 2.5 and 25 μm, respectively. C) ACP52C also exhibits anticancer activity in cisplatin-resistant (CR) cancer cells. GI_{50} values were estimated in various cisplatin-resistant cells and their parent cells. Duplicated data are expressed as means ± SD. Two-tailed unpaired Student's *t*-tests; *, $P < 0.05$; **, $P < 0.01$; ns, non-significant.

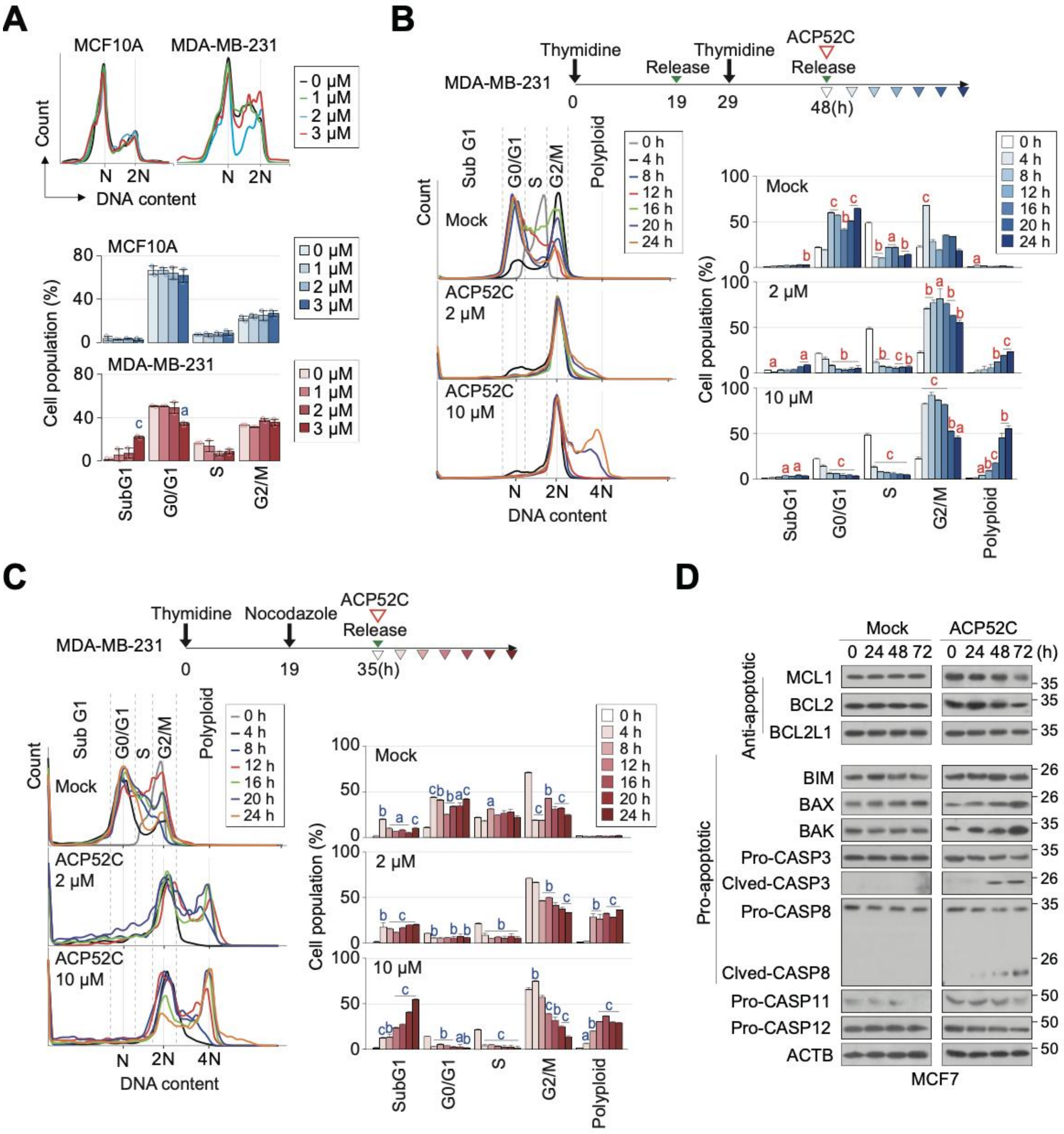

Figure S7. ACP52C induces G2/M arrest and apoptosis

A) Effects of ACP52C on the cell cycle in asynchronous populations. Overlay histograms of the cell cycle analyses (upper panel) and the distribution of cells across the cell cycle (lower panel) 24 h after ACP52C treatment over concentration. Duplicated data are expressed as means ± SD. ANOVA test; a, $P < 0.05$; c, $P < 0.001$. B,C) Cell cycle distribution in the synchronized cell populations following ACP52C treatment over time. Experimental schematics (upper panels), overlay histograms of the cell cycle analyses (lower left panels), and cell cycle distribution (lower right panels) illustrate the effects of ACP52C on cell cycle progression in synchronized cells at the G1/S boundary using thymidine double block (B) or at G2/M boundary following nocodazole treatment (C). Duplicated data are expressed as means ± SD. ANOVA test; a, $P < 0.05$; b, $P < 0.01$; c, $P < 0.001$. D) WBs display changes in the expression of apoptotic marker proteins in mock or ACP52C (2 μM)-treated MCF7 cells.

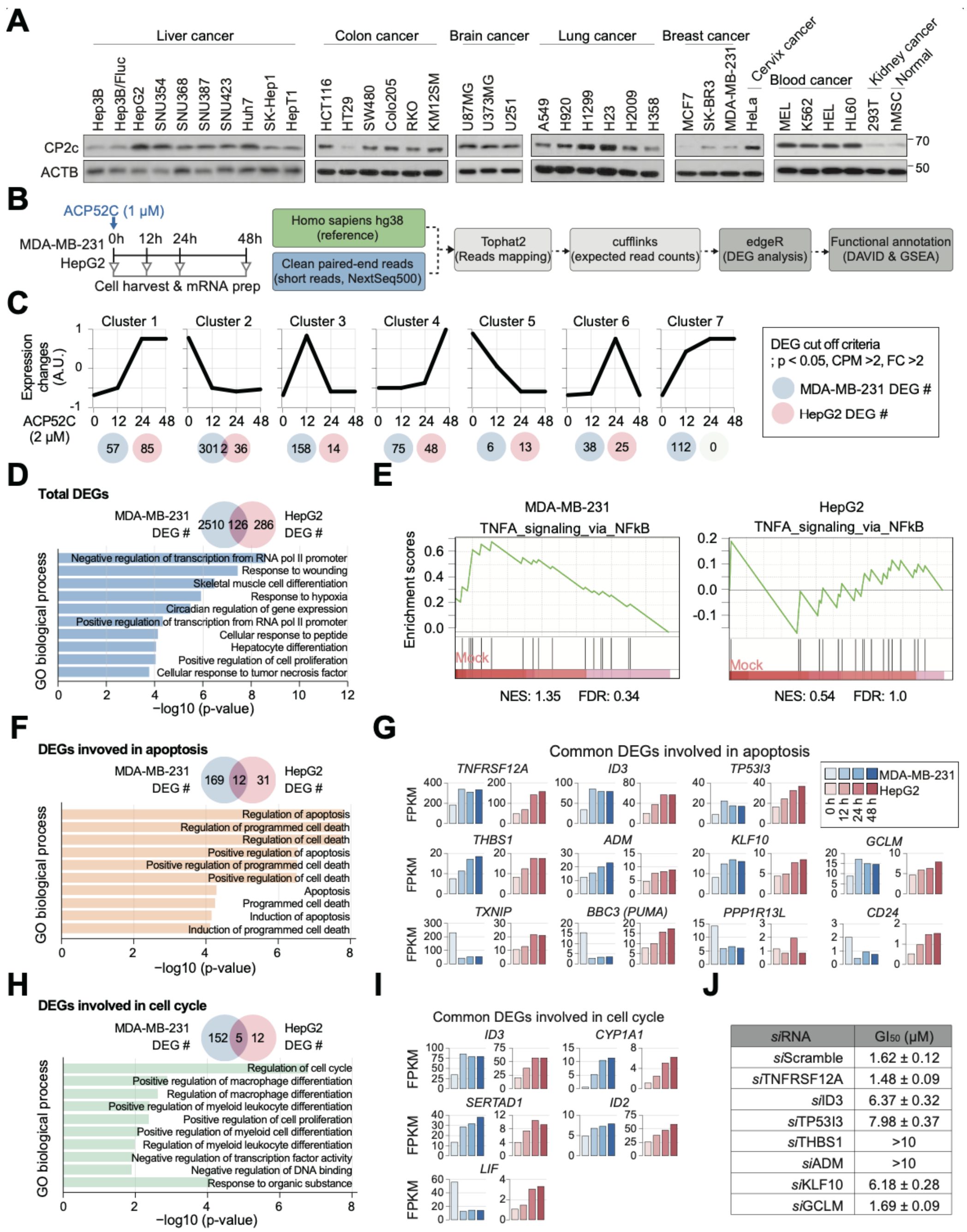

Figure S8. CP2c transcriptional activity is not involved in ACP52C-mediated G2/M arrest and apoptosis induction

A) WBs showing elevated CP2c expression in most of cancer cell lines derived from different origins of tumor. B,C) DEG analyses conducted on MDA-MB-231 and HepG2 cells over time after ACP52C treatment. Experimental scheme (B) and identification of DEGs that exhibit similar expression patterns over time in both cell lines (C). D,E) The GO (D) and GSEA (E) analyses conduced on the common DEGs identified in both cell lines following ACP52C treatment. F-J) GO analyses related to apoptosis- (F) and the cell cycle (H) performed on the common DEGs. Histograms depict changes in mRNA expression for DEGs associated with apoptosis (G) and cell cycle (I). GI_{50} values in MDA-MB-231 cells were estimated from two independent biological replicates, 96 h after each DEG-specific siRNA treatment (J).

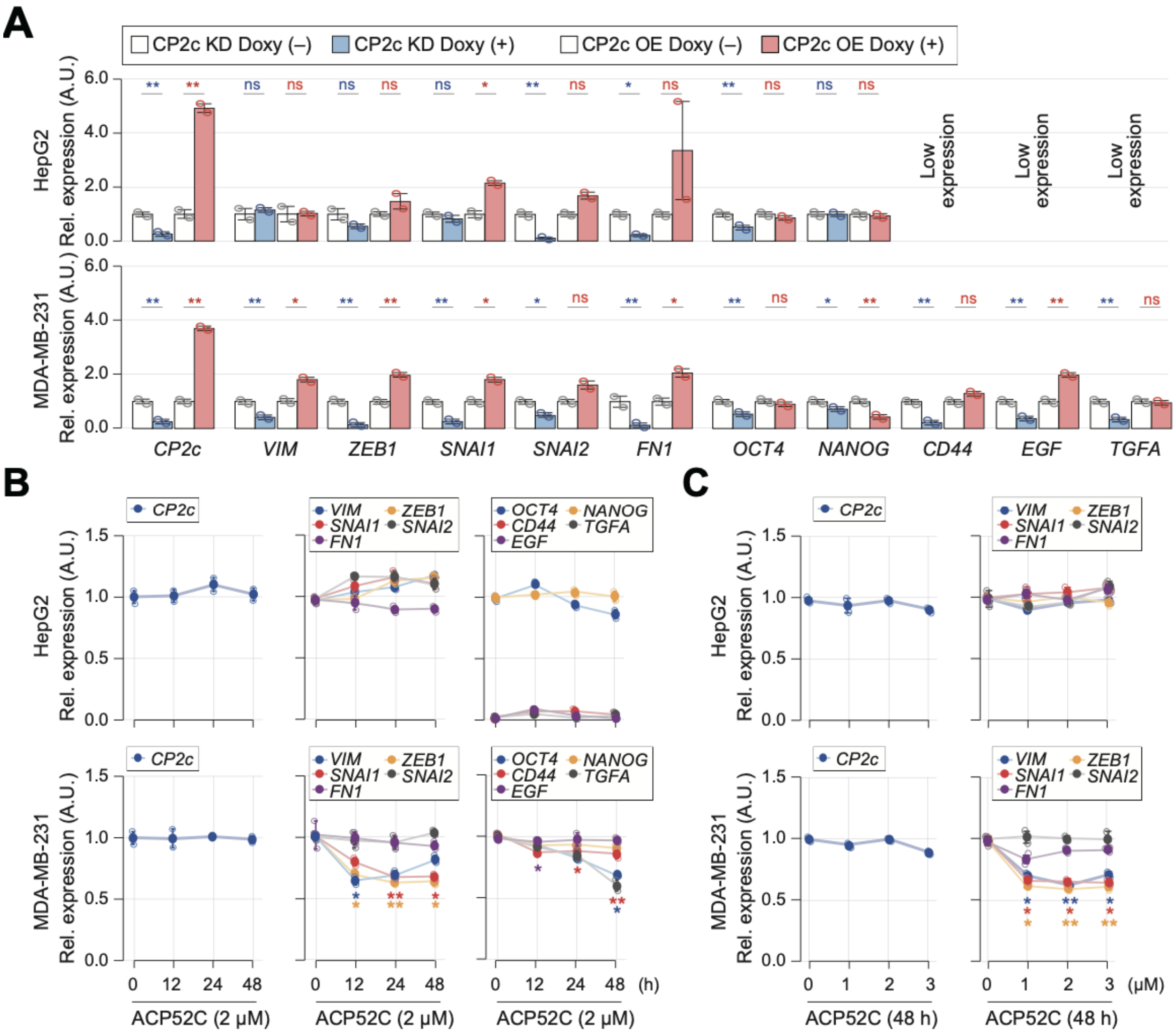

Figure S9. ACP52C does not affect the expression of EMT-related makers, even though they are transcriptional targets of CP2c

A-C) RT-qPCR data showing expression changes of EMT-related genes by CP2c OE or KD (A), or ACP52C treatment over time and dose (B,C). Duplicated data are expressed as means ± SD. Two-tailed unpaired Student's *t*-tests; *, P < 0.05; **, *P* < 0.01; ns, non-significant.

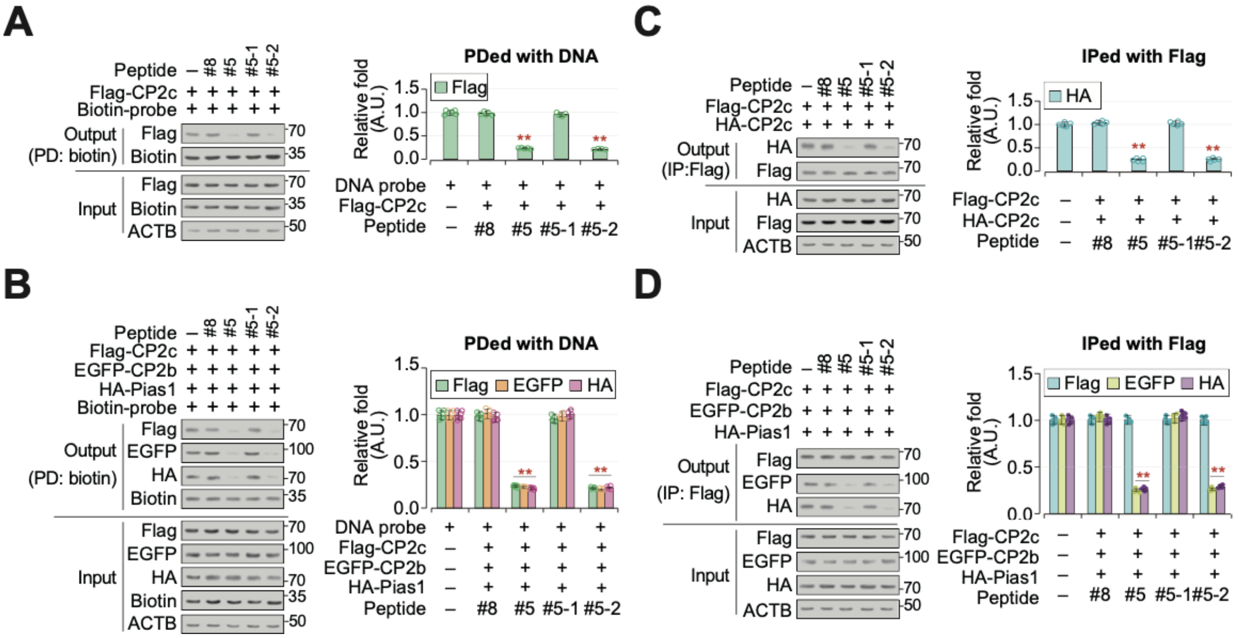

Figure S10. Pep #5-2 inhibits the DNA binding of CP2c complexes by disrupting CP2c transcription factor complexes

A-D) Pep #5-2 efficiently inhibits both the DNA binding ability of CP2c complexes and the CP2c complex formation by itself. DNA-pull down (A,B) and co-IP (C,D) assays to the [C4] complex (A,C) and the CBP complexes (B,D). Data are means ± SD of two independent biological replicates. Two-tailed unpaired Student's *t*-tests; $^{**}P < 0.01$.

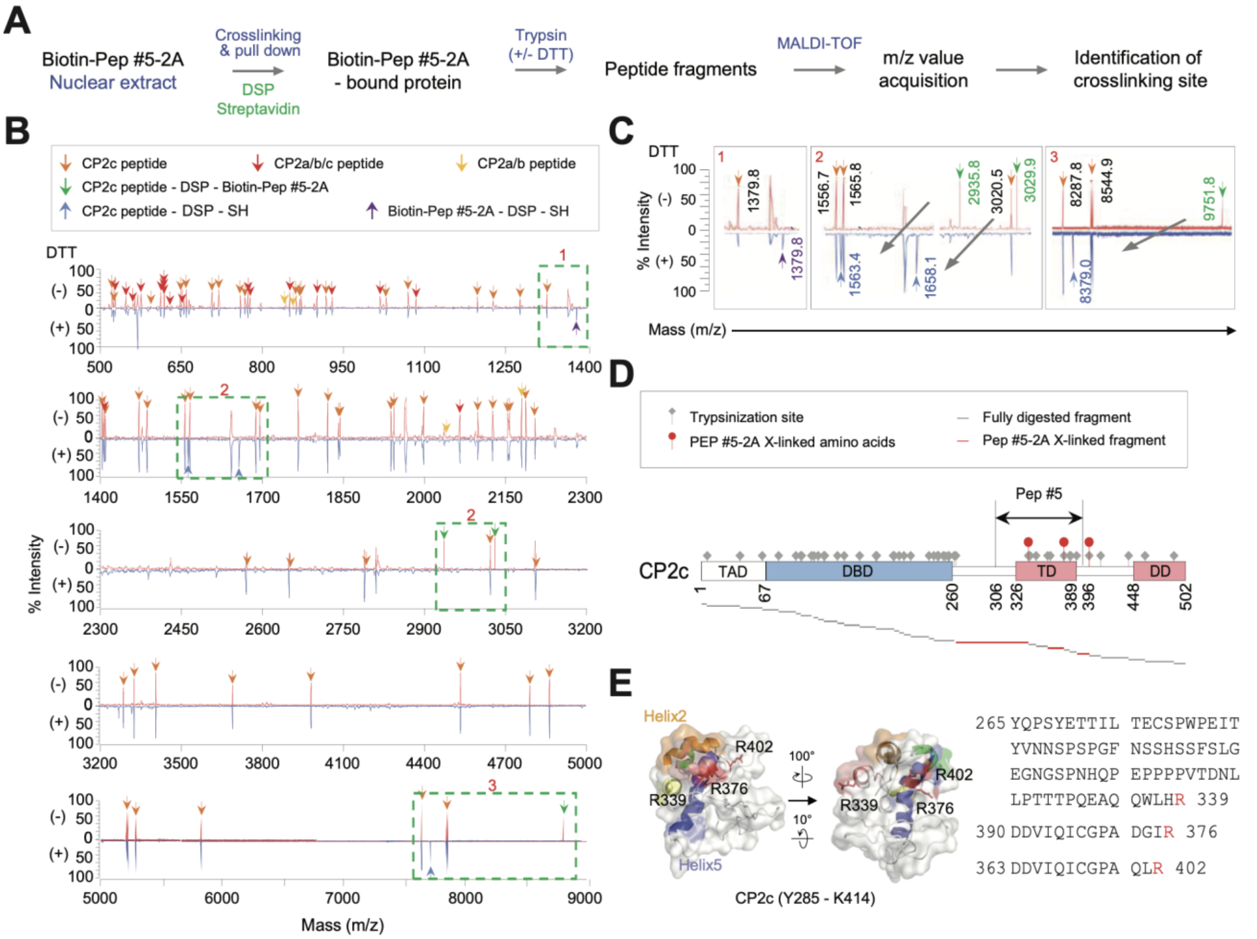

Figure S11. Identification of Pep #5-2 binding nuclear proteins and their specific crosslinked residues.

A) Experimental scheme of DSP crosslinking and mass spectrometry analyses to identify nuclear proteins bound to Pep #5-2 and their specific crosslinked residues. B) MALDI-TOF raw peak data showing nuclear protein profiles analyzed in the presence (+) or absence (−) of DTT after DSP crosslinking and pull down with streptavidin beads. Results from two independent experiments were overlaid. C) Highlights of peptide fragment peaks within dotted green rectangles with numbers in (B), indicating the biotin-Pep #5-2A crosslinked CP2c fragments (long arrows) with reduced masses (m/z) upon DTT treatment. Green colored mass (m/z) scores; peptide fragments that disappeared by DTT treatment, blue colored ones; those appeared by DTT treatment. D,E) Pep #5-2 specifically interacts with amino acids on one surface of the CP2c TD. (D) Schematic diagram illustrating biotin-Pep #5-2A crosslinked sites (red filled circles) in the CP2c protein and biotin-Pep #5-2A crosslinked CP2c fragments after trypsin digestion (red horizontal lines). (E) Three-dimensional structure of the CP2c TD domain, with the biotin-Pep #5-2A crosslinked amino acids (R339, R376, and R402) highlighted. Amino acid sequences of each crosslinked peptide fragment containing cross-linked amino acids (red colored letter) are shown on the right side. The TD domain's helixes 1 through 5 are denoted with ribbons in red, orange, yellow, green, and blue, respectively.

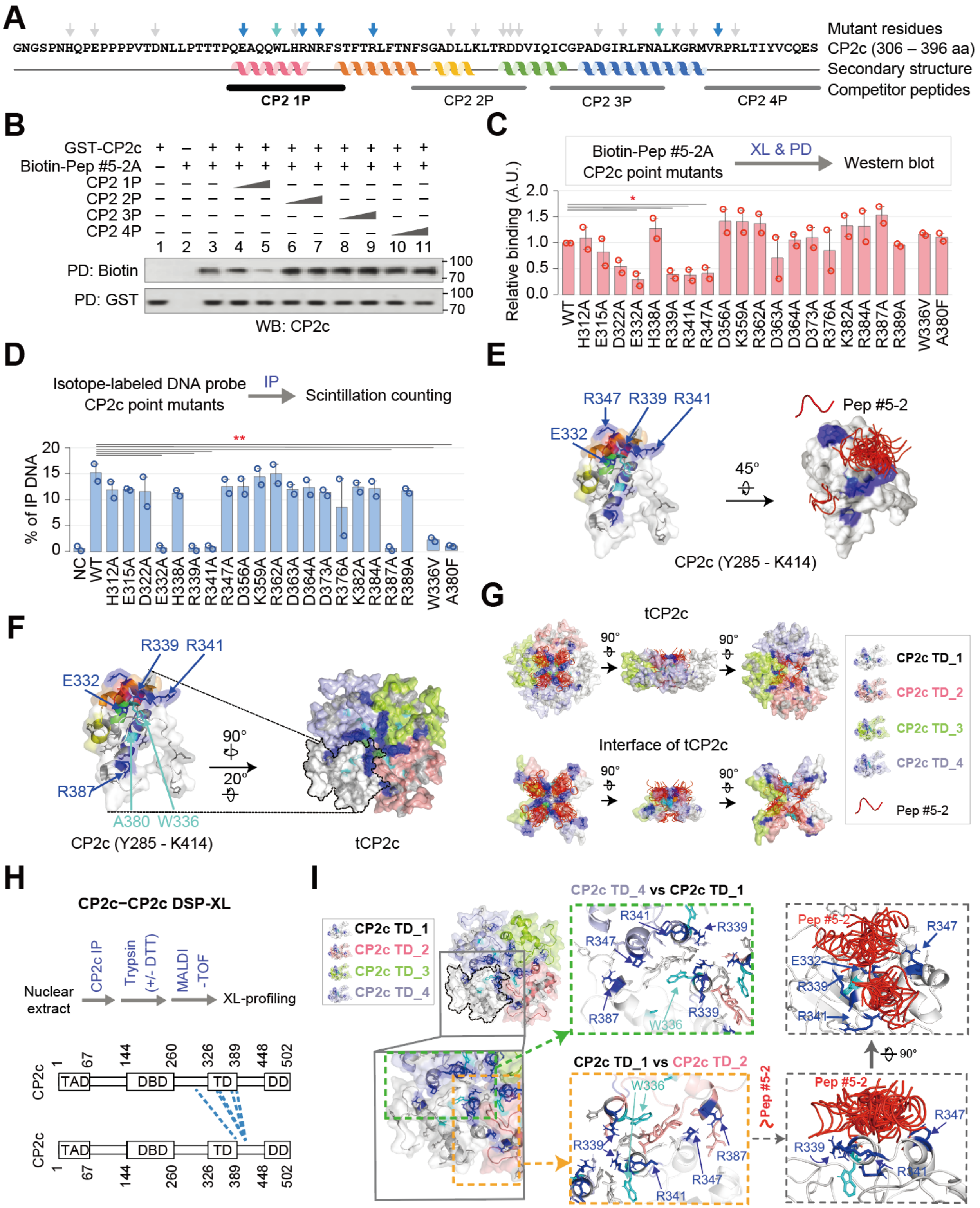

Figure S12. Pep #5-2 binds to interaction junctions within the tCP2c complex disrupting the formation of the complex

A) The CP2c TD domain (306 to 396 amino acids) with secondary structural characters, competitor peptides used in (B), and point mutation residues (vertical arrows) used in (C) and (D). Blue- and cyan-colored

vertical arrows indicate the charged residues or additional Ala mutations in non-charged residues important for Pep #5-2 binding and/or for CP2c complex formation, respectively. B) Competition DNA-pull down analyses identifying CP2 1P as a specific inhibitor for DNA binding of CP2c complexes. C,D) Effects of Ala mutant on Pep #5-2 binding (C) and DNA binding (D). Data are represented as means ± SD of two independent biological replicates. Two-tailed unpaired Student's *t*-tests; *P < 0.05; **P < 0.01. E-G) Structural model of Pep #5-2 binding to the interaction junctions of CP2c TD tetramer. Mapping of the Pep #5-2 binding region and residues important for tCP2c complex formation to the CP2c TD model obtained by I-TASSER and MODELLER (E). A HADDOCK model of the TD tetramer in the tCP2c complex (F), where Pep #5-2 binding occurs in the interaction junctions of CP2c in the complex. Three-dimensional views of the integral CP2c TD tetramer (top) and the interaction junctions among subunits (bottom) in the tCP2c complex, highlighting the Pep #5-2 binding locations (G). H) CP2c interacts each other through residues around TD, as revealed by DSP crosslinking in solution. I) Close-up view of the Pep #5-2-targeting CP2c-CP2c interaction junctions in the [C4] complex. Residues important for [C4] complex formation are in the interface of CP2c TD tetramer, where residues important for Pep #5-2 binding are partially overlapped. Each of CP2c monomers is color-coded in CP2c TD tetramer (top, left). Residues important for Pep #5-2 binding and/or [C4] complex formation are marked by blue (for charged residues) and cyan (for W336 and A380). Orange- and green-colored dotted rectangles are junctions between adjacent CP2c monomer, showing the close-up views of the binding interface between CP2c TD_1 (white) and TD_2 (salmon) and between CP2c TD_1 (white) and TD_4 (light blue) with important residue information. Close-up views of Pep #5-2 binding to CP2c TD_1 is shown (right).

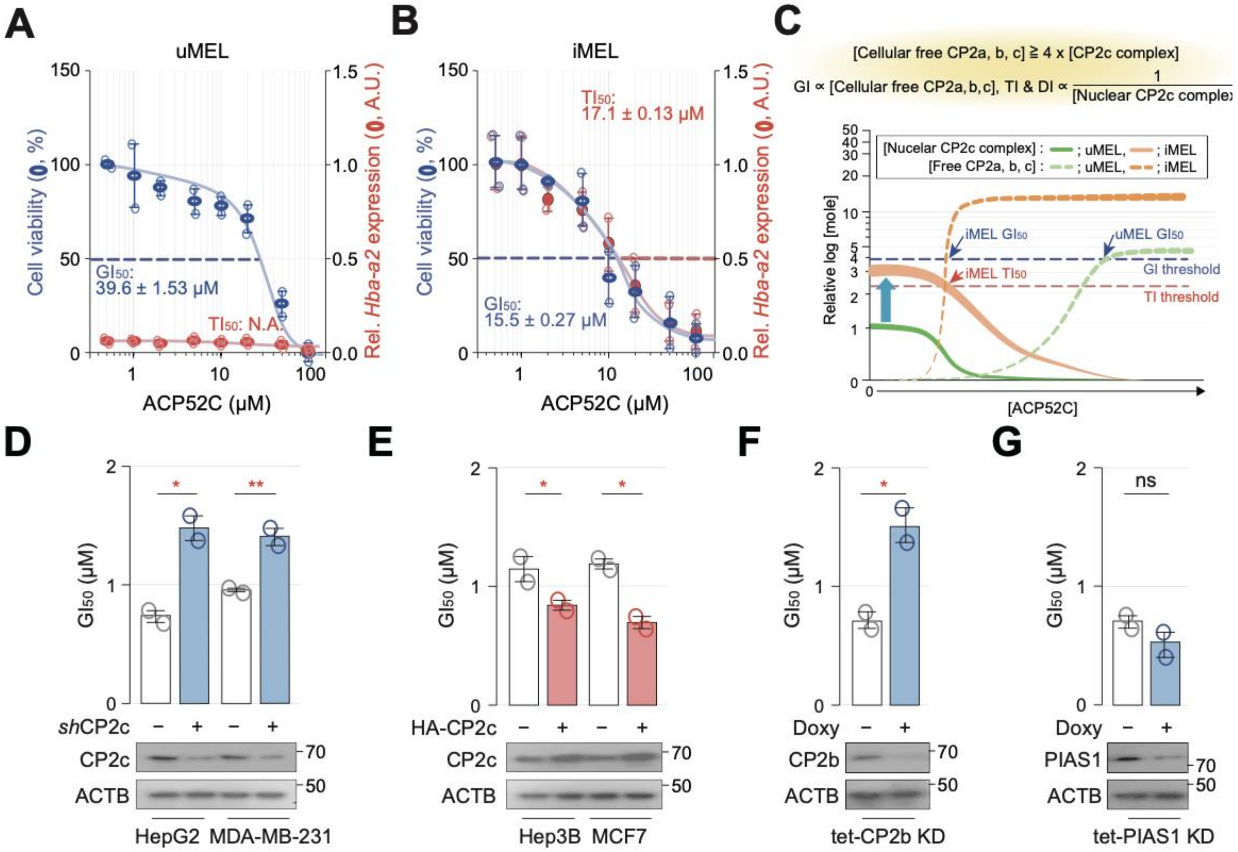

Figure S13. The relationship between ACP52C-mediated growth inhibition and transcriptional inhibition

A,B) ACP52C-mediated growth inhibition and transcriptional inhibition of the α-globin gene during MEL cell differentiation in vitro. Cell viability and α-globin mRNA expression in uMEL (A) and iMEL cells (B) at 36 h after ACP52C treatment of various doses. It is note that uMEL cells exhibit marginal α-globin gene expression due to limited CP2c transcriptional complexes, while iMEL cells actively express the α-globin gene with approximately three-fold induction of CP2c and CP2b at the mRNA level during differentiation. Cell viability and relative *Hba-a2* mRNA expression data are presented as means ± SD of two independent biological replicates. GI_{50} and TI_{50} values estimated from two independent biological replicates. C) Schematic explaining the relationship between GI_{50} values and TI_{50} values of the α-globin mRNA expression in uMEL and iMEL cells. D-G) Anticancer activity of ACP52C correlates with cellular concentration of both CP2c and CP2b, but not with PIAS1. Modulation of GI_{50} values in cancer cells by CP2c KD (D) or CP2c OE (E), and in MDA-MB-231 cells by CP2b KD (F) or PIAS1 KD (G), at 96 h after ACP52C treatment. GI_{50} values were estimated from the cell growth inhibition profiles. Duplicated data are expressed as means ± SD. Two-tailed unpaired Student's *t*-tests; *, $P < 0.05$; **, $P < 0.01$; ns, non-significant.

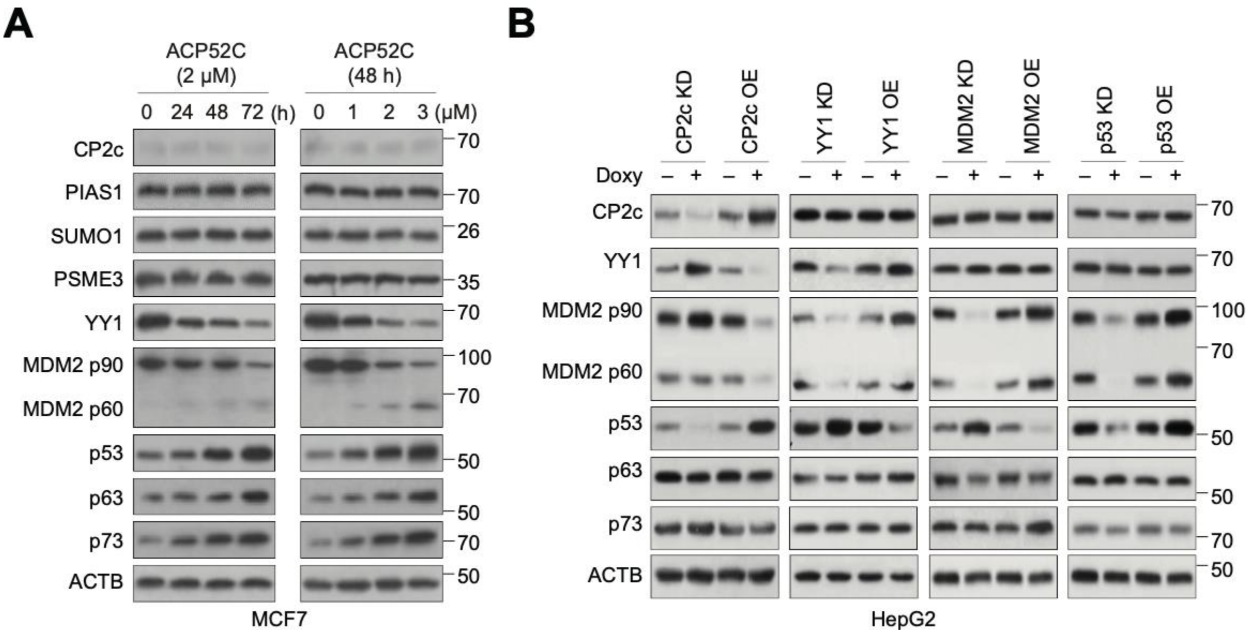

Figure S14. ACP52C modulates signaling in the CP2c/YY1/MDM2 p90/p53 axis

A) WBs demonstrating the modulation of proteins in ACP52C-treated MCF7 cells over time (left) and dose (right). B) WBs illustrating the modulation of proteins in HepG2 cells through Tet-inducible KD or OE of CP2c, YY1, MDM2, or p53.

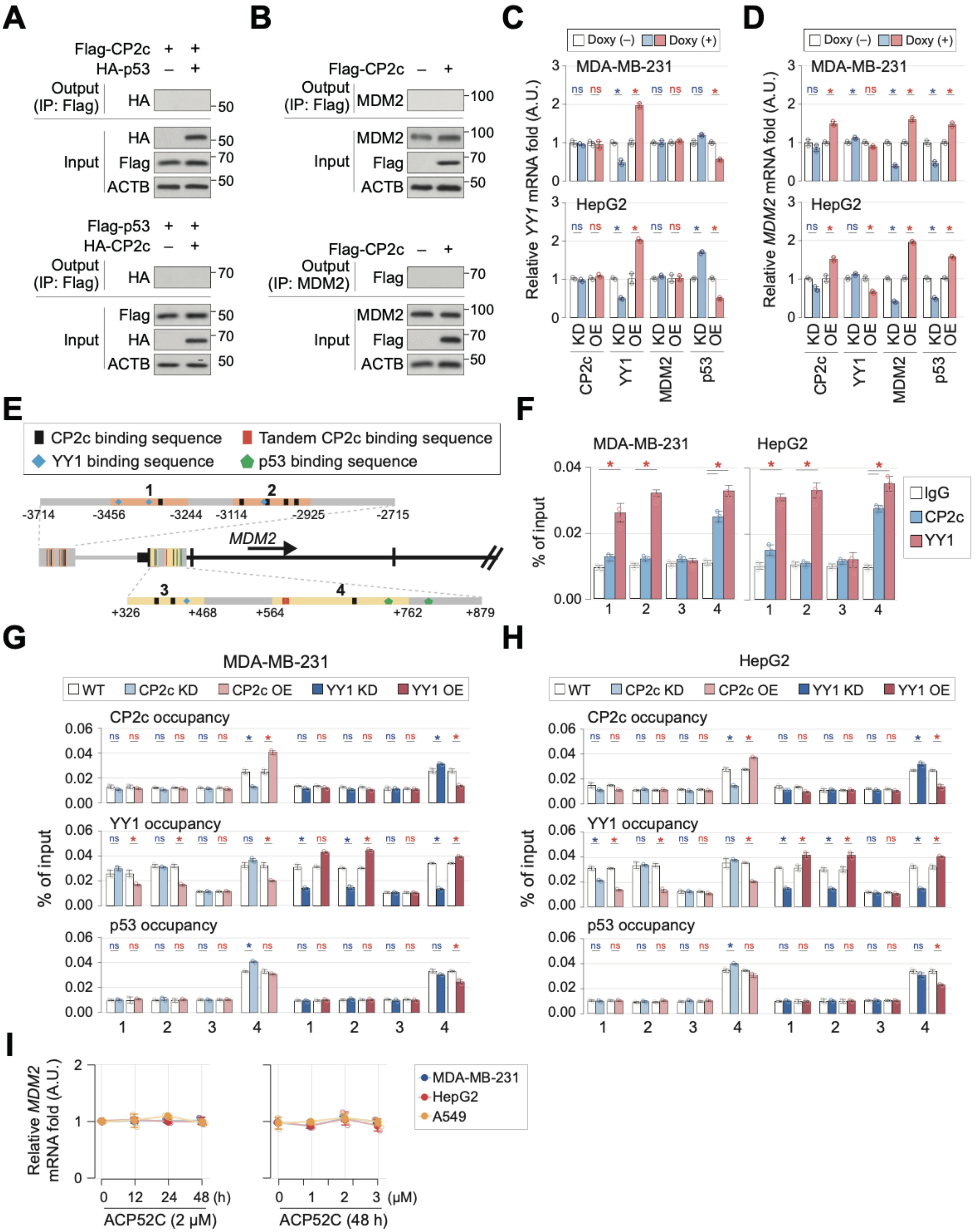

Figure S15. ACP52C modulates the expression of CP2c/YY1/MDM2 p90/p53 axis proteins in a transcription activity-independent manner

A,B) Co-IPs demonstrate that CP2c does not directly bind to either p53 (A) or MDM2 p90 (B) in 293T cells. C,D) Transcriptional changes of YY1 (C) and MDM2 p90 (D) were monitored in MDA-MB-231 cells by conditionally modulating the expression of CP2c, YY1, MDM2 p90, or p53. Duplicated data are expressed as means ± SD. Two-tailed unpaired Student's *t*-tests; *, $P < 0.05$; ns, non-significant. E-I) *MDM2* transcription is induced by CP2c but not by ACP52C. (E) Schematic drawing of the *MDM2* gene and its regulatory regions. Regions 1-4 were tested by ChIP-qPCR. Open vertical arrows represent target regions for PCR analyses. (F-H) Factor occupancy tests in the promoter and enhancers of the *MDM2* gene by ChIP-qPCR. Quantification of each factor binding in wildtype cells (F), or by modulation of CP2c and YY1 expression in MDA-MB-231 (G) and HepG2 (H) cells. Duplicated data are expressed as means ± SD. Two-tailed unpaired Student's *t*-tests; *, $P < 0.05$; **, $P < 0.01$; ns, non-significant. (I) *MDM2* transcriptional levels are presented in MDA-MB-231, HepG2, and A549 cells with ACP52C treatment over time. Duplicated data are expressed as means ± SD.

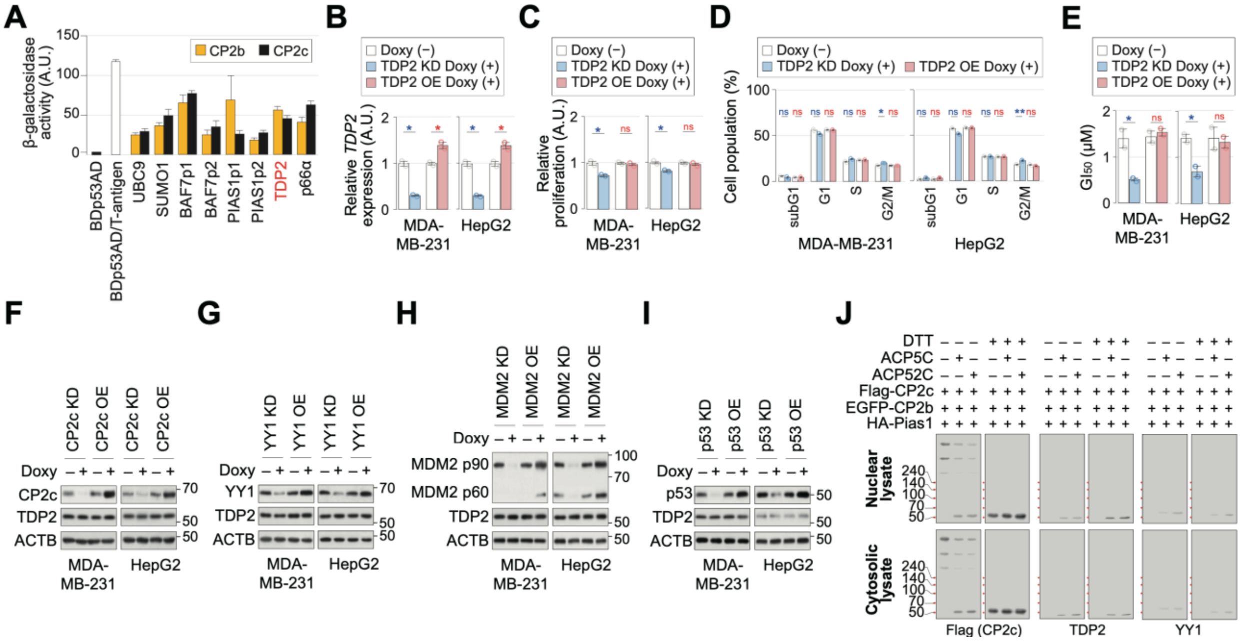

Figure S16. Identification and characterization of TDP2 as one of CP2c interaction proteins

A) Identification of TDP2 as one of CP2c-interacting proteins by yeast 2-hybrid. Duplicated data are expressed as means ± SD. B-E) TDP2 KD, but not OE, inhibits cell proliferation and cell cycle progression. (B) RT-qPCR confirms Tet-inducible TDP2 KD and OE in MDA-MB-231 (LM1 line) and HepG2 cells. Graphical presentations of cell proliferation (C) and cell cycle distribution (D) by TDP2 KD or OE. Duplicated data are expressed as means ± SD. Two-tailed unpaired Student's *t*-tests; *, $P < 0.05$; **, $P < 0.01$; ns, non-significant. (E) Modulation of GI_{50} values of ACP52C in cells by TDP2 KD or OE. Duplicated data are expressed as means ± SD. Two-tailed unpaired Student's *t*-tests; *, $P < 0.05$; ns, non-significant. F-I) WBs showing that TDP2 protein is not modulated by CP2c/YY1/MDM2 p90/p53 axis proteins. It is of noting that TDP2 WBs were created after deprobing/reprobing of blots in Figure 4D; Figure S14B, and control immunoblots corresponding to CP2c, YY1, MDM2 p90, p53, and ACTB were the same to those in Figure 4D; Figure S14B, Supporting Information. J) DSP XL-WB demonstrates that TDP2 and YY1 bind to CP2c monomers liberated by ACP52C treatment, but not to CP2c in the complexes.

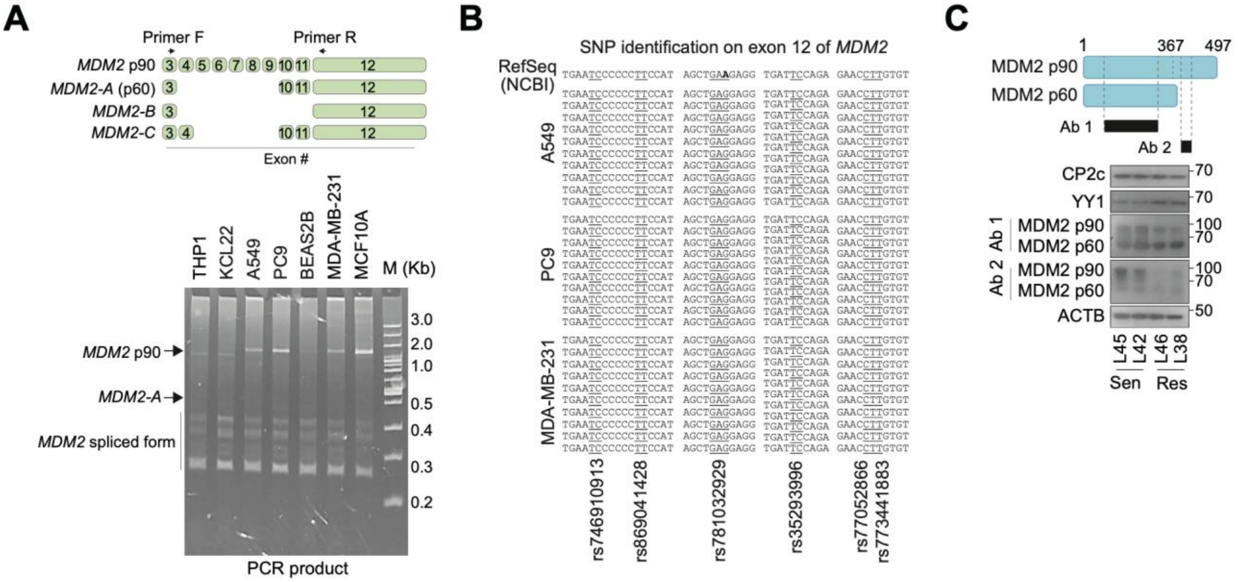

Figure S17. ***MDM2*** **alternative splicing or SNPs is not involved in the ACP52C resistance**

A) The schematic illustrates a PCR strategy for detecting various known *MDM2* splicing variants (top) and displays the PCR products in various ACP52CGK-sensitive and -resistant cells (bottom). B) Comparisons of SNPs in the *MDM2* exon 12 regions in ACP52CGK-sensitive (MDA-MB-231) and -resistant (PC9 and A549) cells. DNA sequences amplified by genomic PCR from each cell were cloned into the plasmid vector, sequenced, and compared to the RefSeq. C) WBs demonstrate that ACP52C resistance in human liver cancer patient samples is correlated with the expression level of C-terminal truncated MDM2 p60.

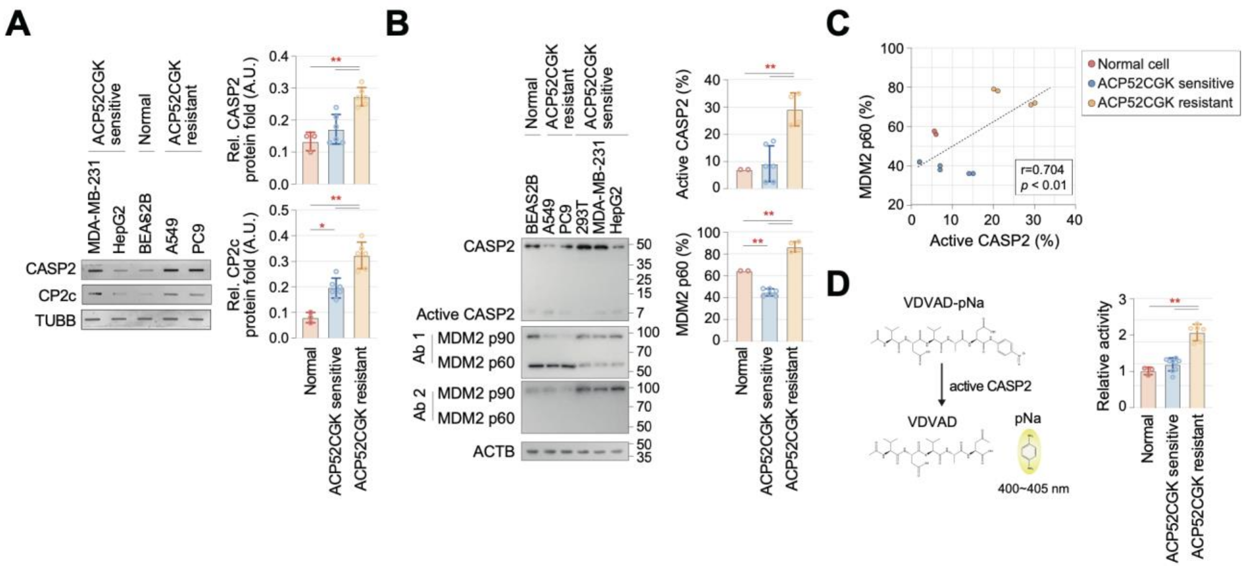

Figure S18. CASP2-mediated MDM2 p90 cleavage is involved in ACP52CGK resistance in cancer cells

A) ACP52CGK-resistant cancer cells exhibit significantly higher expression of CASP2 compared to ACP52CGK-sensitive cells. Expressions of CASP2 and CP2c in ACP52CGK-sensitive and -resistant cancer cell lines are accessed using slot blots. Representative slot blots (left) and their relative expression to β-tubulin (right) are shown. Triplicated data are expressed as means ± SD. Two-tailed unpaired Student's *t*-tests; *, $P < 0.05$; **, $P < 0.01$. B) ACP52CGK-resistant cells demonstrate significantly higher CASP2 activity and MDM p60 production when compared to ACP52CGK-sensitive cells. Expressions of CASP2 and MDM2 p90 and p60 in ACP52CGK-sensitive and -resistant cancer cell lines are accessed by WBs. Representative WBs (left) and the quantified fractions of the active CASP2 and the MDM2 p60 (right) are shown. Duplicated data are expressed as means ± SD. Two-tailed unpaired Student's *t*-tests; **, $P < 0.01$. C) The production of MDM2 p60 shows a positive correlation with CASP2 activity in cancer cells according to Pearson's correlation test. D) Experimental validation demonstrates that ACP52CGK-resistant cells have significantly higher cleavage activity of the CASP2-specific substrate compared to ACP52CGK-sensitive cells. Triplicated data are expressed as means ± SD. Two-tailed unpaired Student's *t*-tests; **, $P < 0.01$.

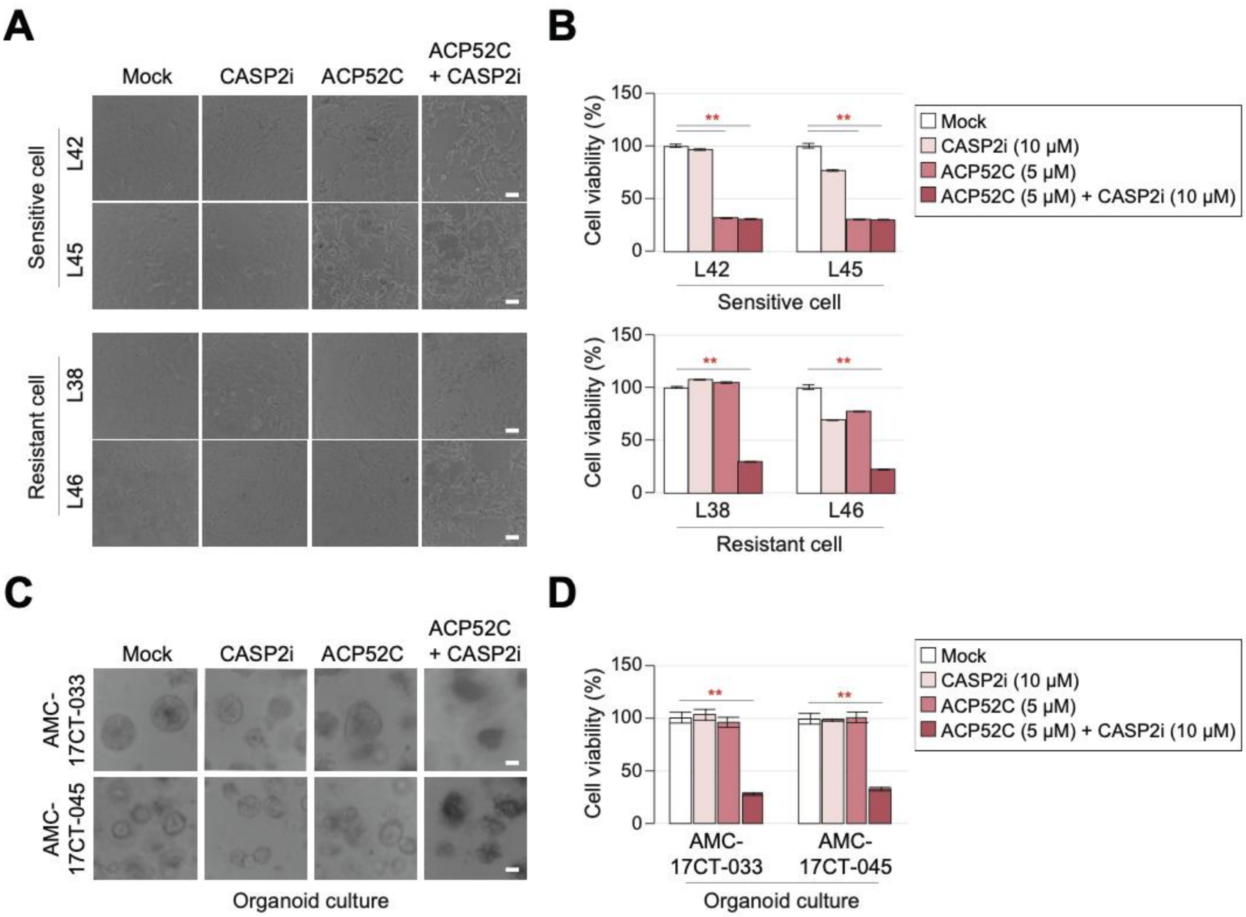

Figure S19. The combined treatment of ACP52C and the CASP2 inhibitor sensitizes ACP5C2-resistance in human cancers

A,B) Tests of cell death induction in primary cultured cells derived from human liver cancer patients by treatments of ACP52C alone or in combination with a CASP2 inhibiting peptide. Representative photographs of cells (A) and relative cell viability (B). Duplicated data are expressed as means ± SD. Two-tailed unpaired Student's *t*-tests; **, $P < 0.01$. C,D) Tests of cell death induction in organoids derived from human colon cancer patients by treatments of ACP52C alone or in combination with a CASP2 inhibiting peptide. Representative photographs of organoids (C) and relative cell viability (D). Duplicated data are expressed as means ± SD. Two-tailed unpaired Student's *t*-tests; **, $P < 0.01$.

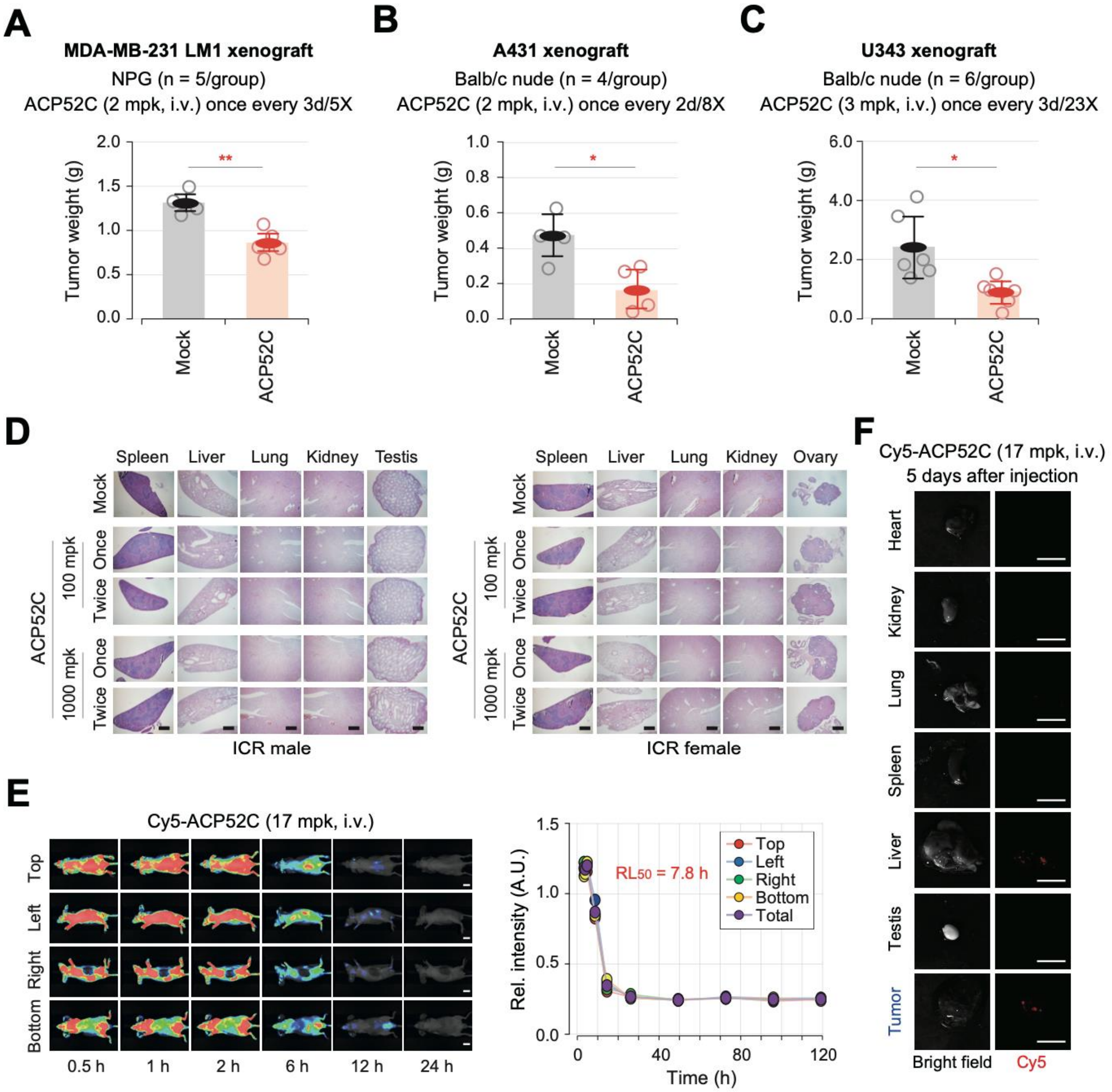

Figure S20. ACP52C efficacies in vivo

A-C) Anticancer effects of ACP52C in xenograft model mice. ACP52C reduces tumor weight in MDA-MB-231 LM1 (A), A431 (B), and U343 (C) CDX mice. Data are expressed as means ± SD, and the number of individuals per experimental group is indicated in the figure. Two-tailed unpaired Student's *t*-tests; *, $P < 0.05$; **, $P < 0.01$. D) ACP52C does not show any adverse side effects in the repeated toxicity test in the major organ of normal male and female mice (n = 2/group). The mock group received saline as the vehicle, while the experimental group received injections of ACP52C at doses of 100 mpk or 1000 mpk. "Once" indicates organs collected from mice sacrificed 4 days after a single injection of the drugs, whereas "Twice" refers to organs collected from mice sacrificed 4 days after receiving drug injections twice every 4 days. Scale bar represents 200 μm. E,F) ACP52C exhibits a short residual presence in the body along with effective tumor targeting. (E) Live imaging of Hep3B cell xenograft mice after intravenous administration of Cy5-ACP52C via the tail vein (left). The estimated residual levels of Cy5-ACP52C in the body over time were determined by quantifying the fluorescence intensity of images (right). RL_{50} represents the required time for a 50% retention of fluorescence level. (F) Bright field and fluorescence images of the

major organs and tumor tissue at 5 days after Cy5-ACP52C injection. Scale bar represents 1 cm. It is worth noting that there were no animal deaths related to drug administration in any of the animal studies, although there was one animal death in some treatment groups.

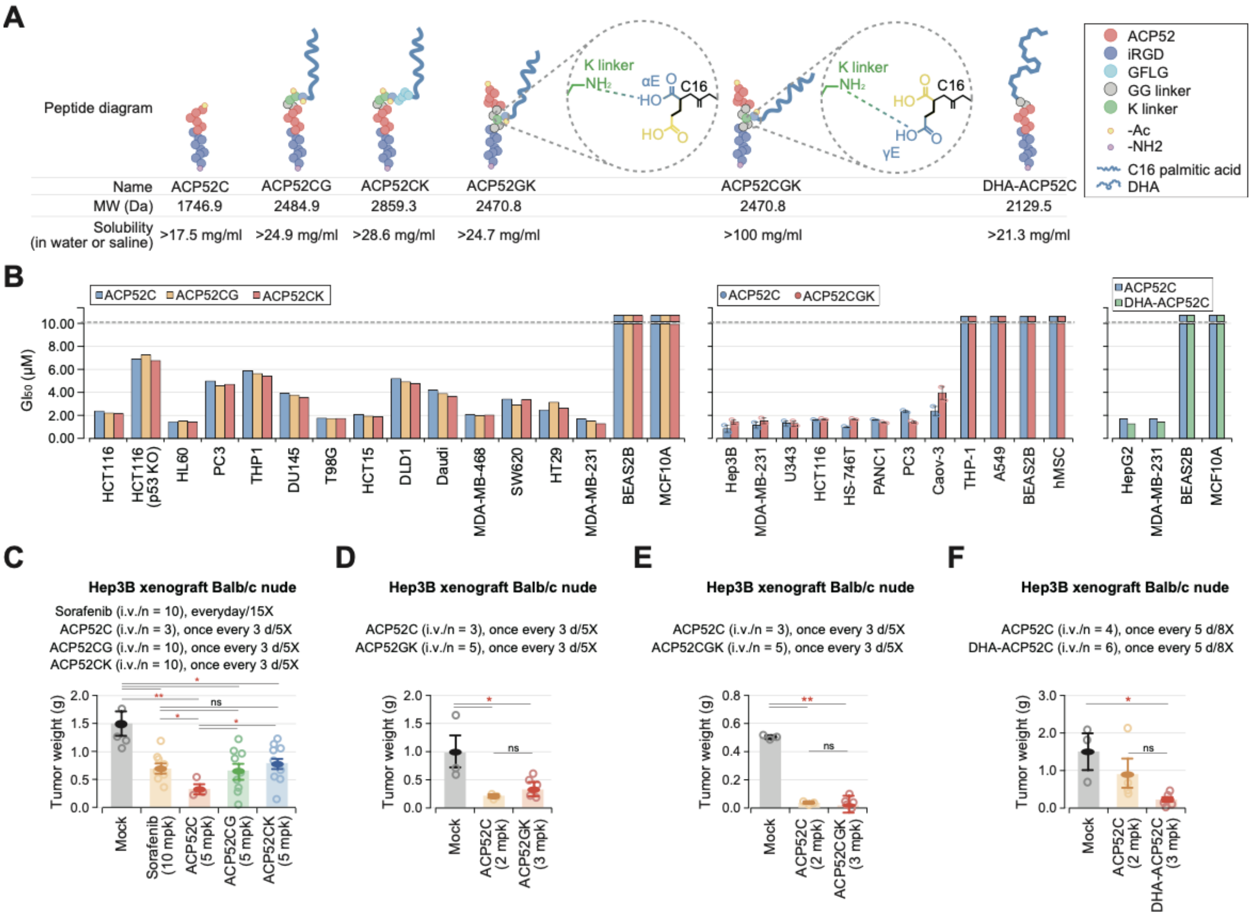

Figure S21. Anticancer activities of the fatty acid-conjugated derivatives of ACP52C

A) A schematic representation depicting the structural diagrams, molecular weights, and minimum solubilities (in water or saline) of ACP52C and its derivatives. B) GI_{50} values of ACP52C derivatives in the selected cell lines. C-F) In vivo efficacies of ACP52C derivatives in the Hep3B CDX mice. Tumor weight in the sacrificed mice after completion of drug administration schedule was expressed as means ± SE. ANOVA tests; *, $P < 0.05$; **, $P < 0.01$; ns, non-significant. It is worth noting that there were no animal deaths related to drug administration in any of the animal studies, although there was one animal death in some treatment groups.

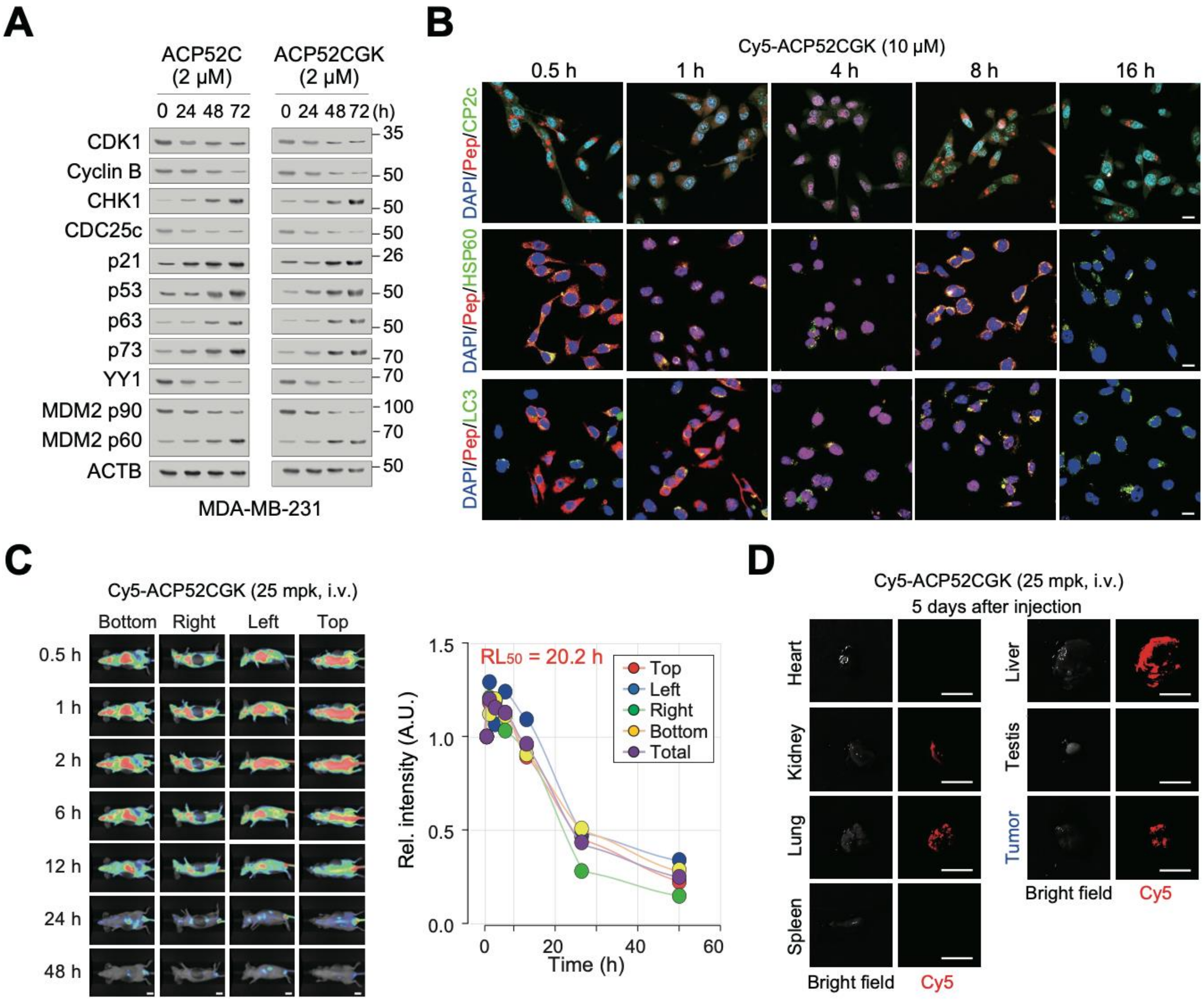

Figure S22. ACP52CGK shows improved pharmacokinetics compared to ACP52C

A) WBs show that ACP52CGK retains the same mode-of-action of ACP52C, exhibiting similar effects on markers expression. B) Time-dependent subcellular localization of ACP52CGK. Cy5-ACP52C (10 μM) was supplemented to the culture medium for 30 min to MDA-MB-231 cells, and IF was performed at different time points after replenishing the fresh medium to observe the subcellular localization of ACP52C. Scale bar represents 10 μm. C,D) ACP52CGK shows an improved residual presence in the body with effective tumor targeting. (C) Live imaging of the Hep3B CDX mouse after intravenous injection of Cy5-ACP52CGK via the tail vein (left). The estimated residual levels of Cy5-ACP52C in the body over time were determined by quantifying the fluorescence intensity of images (right). RL_{50} represents the required time for a 50% retention of fluorescence level. (D) Bright field and fluorescence images of the major organs and tumor tissue at 5 days after Cy5-ACP52CGK injection. Scale bar represents 1 cm.

A

ACP52CGK (5 mpk, i.v., 48 h)

PK Parameters	ACP52CGK
Dose (mg/kg)	5
C_0 (ng/mL)	79175
$T_{1/2}$ (h)	5.26
Vd_{ss} (L/kg)	0.124
Cl(mL/min/kg)	0.275
T_{last} (h)	48
$AUC_{0\text{-}last}$ (ng.h/mL)	302798
$AUC_{0\text{-}24}$ (ng.h/mL)	290286
$AUC_{0\text{-}inf}$ (ng.h/mL)	303344
$MRT_{0\text{-}last}$ (h)	7.44
$MRT_{0\text{-}inf}$ (h)	7.53
AUC_{Extra} (%)	0.180
$AUMC_{Extra}$ (%)	1.33

B

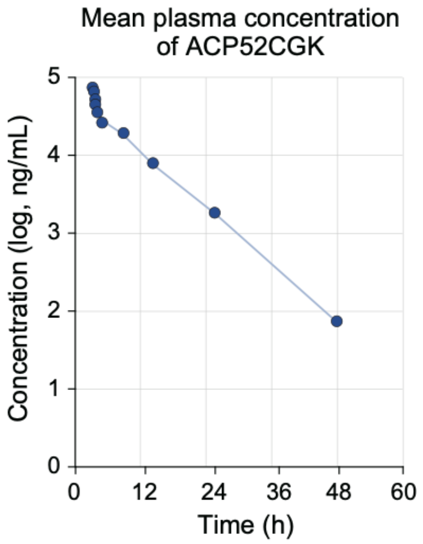

Figure S23. Pharmacokinetics analysis of ACP52CGK

A) PK parameters of ACP52CGK in CD-1(ICR) mice, calculated using the noncompartmental analysis model 201 of Phoenix WinNonlin version 6.3. B) Mean plasma concentration of ACP52CGK at 0.033, 0.083, 0.25, 0.5, 1, 2, 6, 12, 24, and 48 h after dosing.

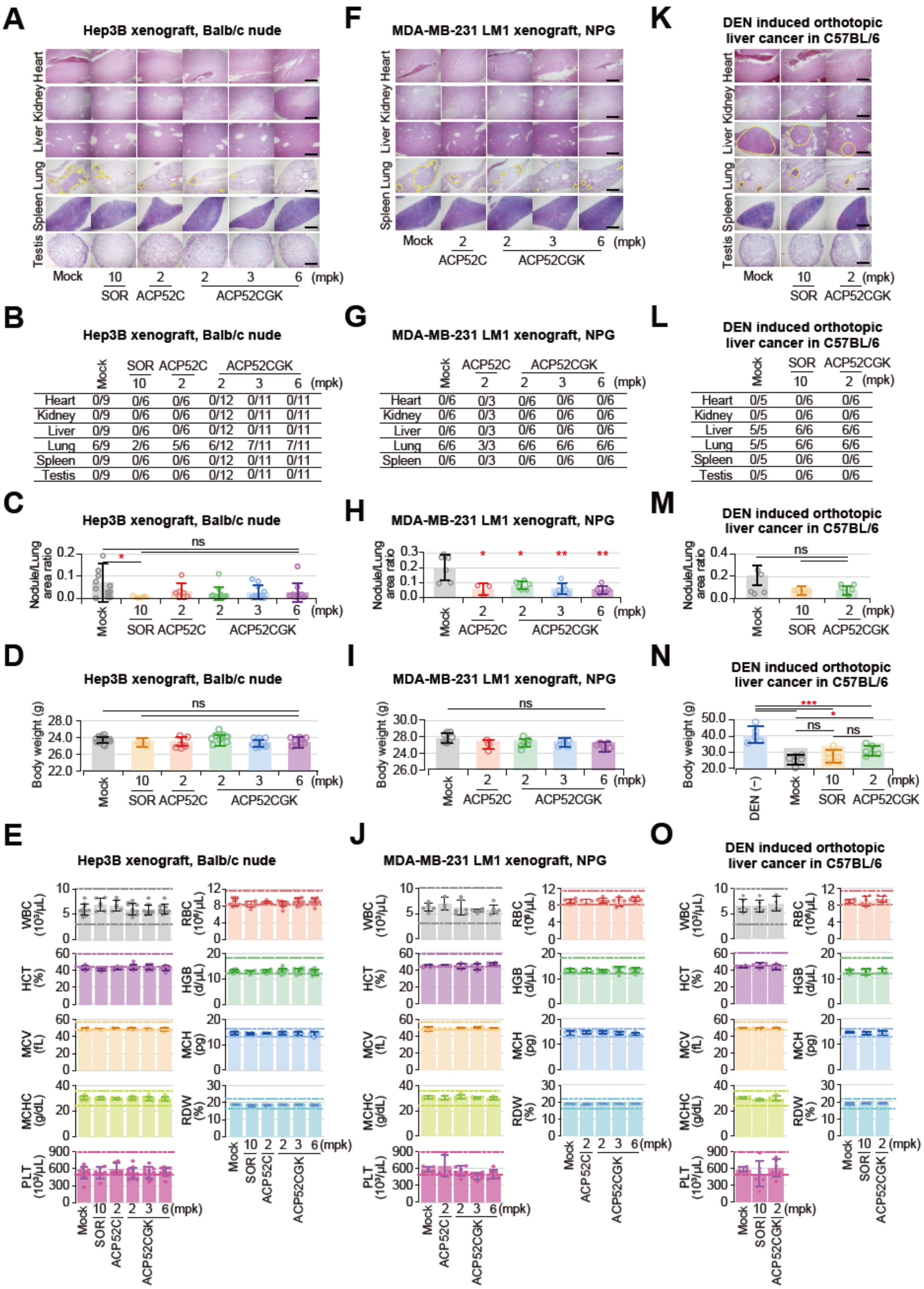
A
Hep3B xenograft, Balb/c nude
Heart
Kidney
Liver
Lung
Spleen
Testis
Mock
10
SOR
2
ACP52C
2
3
6
(mpk)
ACP52CGK
F
MDA-MB-231 LM1 xenograft, NPG
K
DEN induced orthotopic liver cancer in C57BL/6
B
Hep3B xenograft, Balb/c nude
Mock | SOR 10 | ACP52C 2 | ACP52CGK 2 | 3 | 6 (mpk)
Heart 0/9 0/6 0/6 0/12 0/11 0/11
Kidney 0/9 0/6 0/6 0/12 0/11 0/11
Liver 0/9 0/6 0/6 0/12 0/11 0/11
Lung 6/9 2/6 5/6 6/12 7/11 7/11
Spleen 0/9 0/6 0/6 0/12 0/11 0/11
Testis 0/9 0/6 0/6 0/12 0/11 0/11
G
MDA-MB-231 LM1 xenograft, NPG
Mock | ACP52C 2 | ACP52CGK 2 | 3 | 6 (mpk)
Heart 0/6 0/3 0/6 0/6 0/6
Kidney 0/6 0/3 0/6 0/6 0/6
Liver 0/6 0/3 0/6 0/6 0/6
Lung 6/6 3/3 6/6 6/6 6/6
Spleen 0/6 0/3 0/6 0/6 0/6
L
DEN induced orthotopic liver cancer in C57BL/6
Mock | SOR 10 | ACP52CGK 2 (mpk)
Heart 0/5 0/6 0/6
Kidney 0/5 0/6 0/6
Liver 5/5 6/6 6/6
Lung 5/5 6/6 6/6
Spleen 0/5 0/6 0/6
Testis 0/5 0/6 0/6
C
Nodule/Lung area ratio
ns
H
M
D
Body weight (g)
I
N
DEN (−)
E
J
O
WBC (10³/μL)
RBC (10⁶/μL)
HCT (%)
HGB (d/μL)
MCV (fL)
MCH (pg)
MCHC (g/dL)
RDW (%)
PLT (10³/μL)

Figure S24. Evaluation of ACP52CGK on metastasis and side effects in three different mouse cancer models

A-O) Hep3B CDX (A-E) and MDA-MB-231 LM1 CDX (F-J) mouse cancer models, as well as the DEN-induced orthotopic liver cancer model (K-O). The number of individuals per experimental group is consistent in the xenograft model corresponding to Figure 7. Representative images of H&E-stained tissue sections from major organs harvested from xenograft mice upon completion of the drug administration (A, F, and K). Histological scoring of tumor-bearing mice/total mice (B, G, and L) and the nodule area/lung area (C, H, and M) in the H&E-stained major organs of tumor-bearing mice. Body weights of mice (D, I, and N) and CBC analysis of each treated group of mice (E, J, and O) at the experiment's conclusion. Data are expressed as means ± SD. The two dotted lines in each graph represent the high and low limits of values in normal mice. Data are expressed as means ± SD. ANOVA tests;*, $P < 0.05$; **, $P < 0.01$; ***, $P < 0.001$; ns, non-significant. Scale bars in (A, F, and K), 500 μm. WBC, white blood cell count; RBC, red blood cell count; HCT, hematocrit; HGB, hemoglobin; MCV, mean corpuscular volume; MCH, mean corpuscular hemoglobin; MCHC, mean corpuscular hemoglobin concentration; RDW, red cell distribution width; PLT, platelet count. It is worth noting that there were no animal deaths related to drug administration in any of the animal studies.

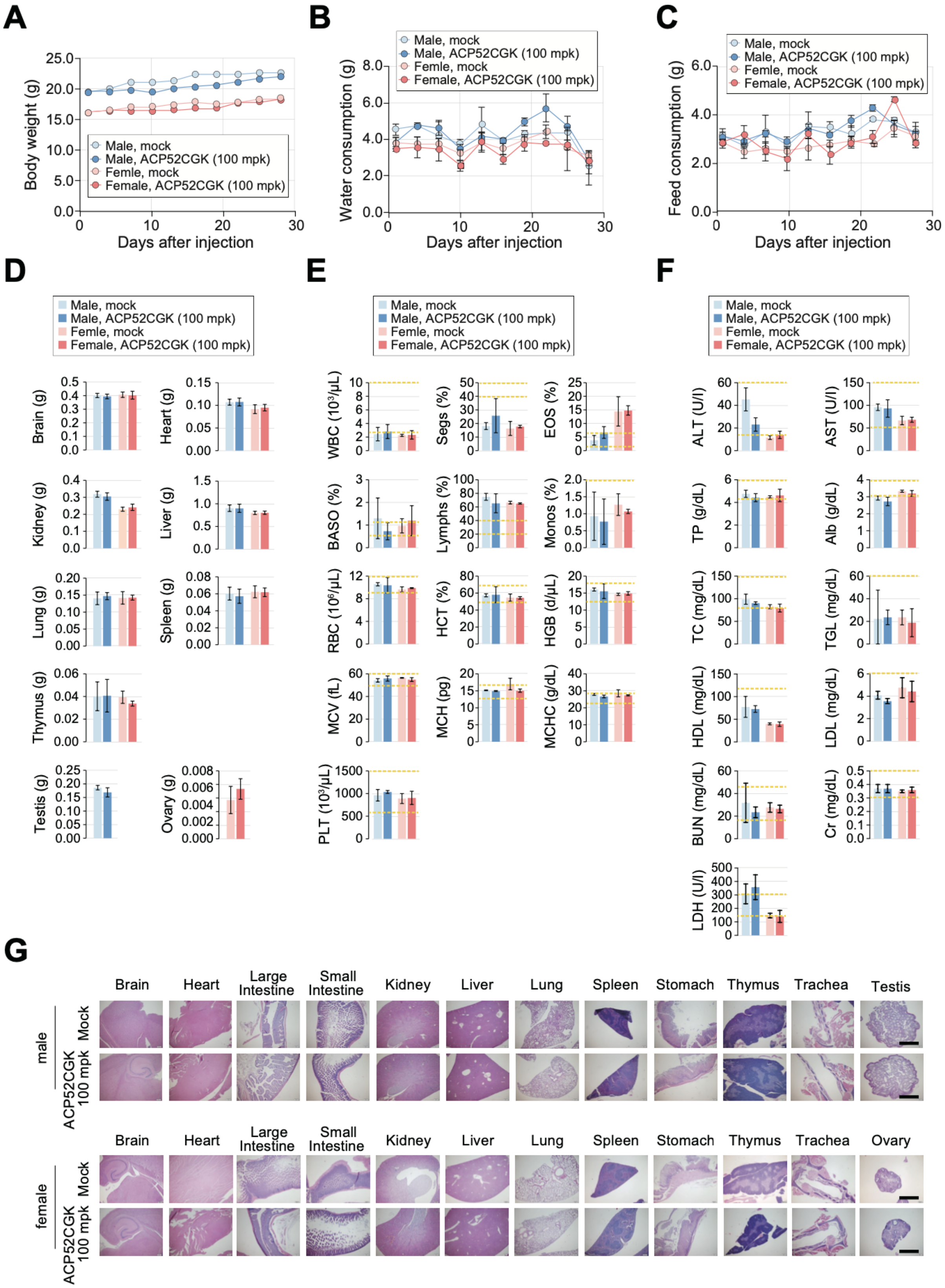

Figure S25. ACP52CGK does not show any adverse side effects in the repeated toxicity test in normal male and female mice

Repeated toxicity test of ACP52CGK were analyzed in ICR mice by intravenous injection of 100 mpk of ACP52CGK daily for 4 weeks (n = 6/group). A-C) Body weight (A), water consumption (B), and food consumption (C) were measured once every 3 days after injection. D-F) Major organ weight (D), CBC (E), and blood chemistry (F) were measured after sacrifice. Data are expressed as means ± SD in (B-F). G) ACP52CGK does not show any adverse side effects in the repeated toxicity test in normal male and female mice. Scale bar represents 200 μm. It is worth noting that there were no animal deaths related to drug administration in any of the animal studies.

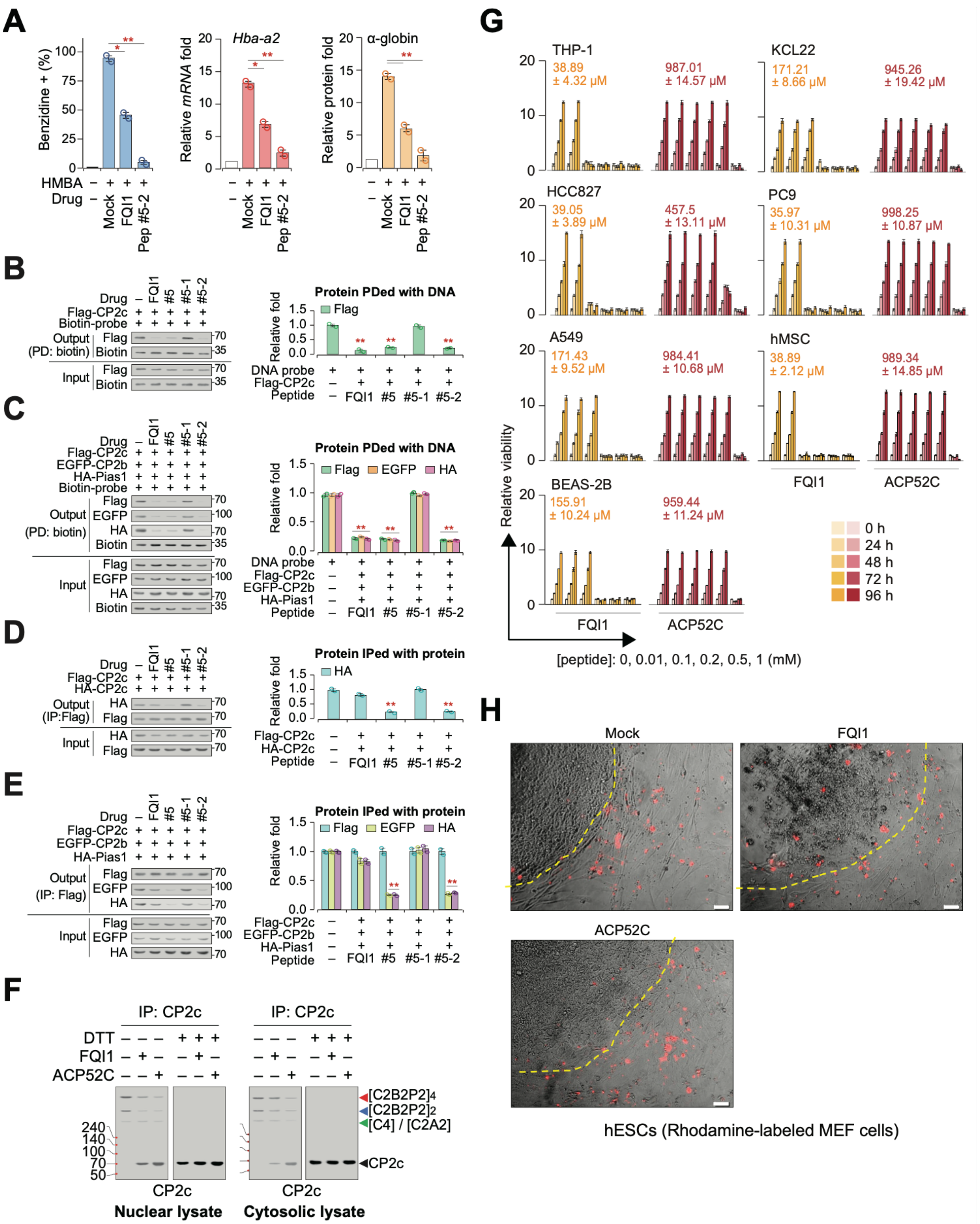

Figure S26. ACP52C is superior to another CP2c-targeting small molecule, FQI1, in terms of efficacy and safety at the level of cultured cells

A) Pep #5-2C (ACP52C) efficiently inhibits functional hemoglobin synthesis (left), and *Hba-a2* globin gene expression at both mRNA (middle) and protein (right) levels in iMEL cells, surpassing the

performance of FQI1. Two micromoles of each Pep #5-2C and FQI1 were added to the cell culture for 2 days. Fractions of benzidine-positive stained cells were counted in MEL cells (n > 100 cells/group) treated with HMBA for 2 days. *Hba-a2* gene expression at mRNA and protein levels were estimated by RT-qPCR and WB, respectively. Data are presented as means ± SD of two independent biological replicates. ANOVA test; * $P < 0.05$, ** $P < 0.01$. B,C) FQI1 efficiently inhibits the DNA binding ability of CP2c complexes similar performance of Pep #5 or Pep #5-2. DNA-pull down assays for the [C4] complex (B) and the CBP complexes (C). Data are presented as means ± SD of two independent biological replicates. Two-tailed unpaired Student's *t*-tests; ** $P < 0.01$. D,E) Pep #5 and Pep #5-2 efficiently inhibits the CP2c complex formation by itself, surpassing the performance of FQI1. Co-IP assays for the [C4] complex (D) and the CBP complexes (E). Data are presented as means ± SD of two independent biological replicates. Student's *t*-tests; ** $P < 0.01$. F) DSP XL-WB demonstrates that ACP52C disrupt CP2c complexes more effectively than FQI1. G) Cell growth inhibition plots and GI_{50} values of FQI1 and ACP52C in various cancer cell lines and normal cells over peptide dose. GI_{50} values estimated from two independent biological replicates. H) Representative images showing that FQI1 induces cell death in hESC cells, whereas ACP52C does not. hESCs were cultured on the Rhodamine-labeled MEF cells. Ten micromoles of each ACP52C and FQI1 were added to the cell culture for 3 days before capturing images of cultures.

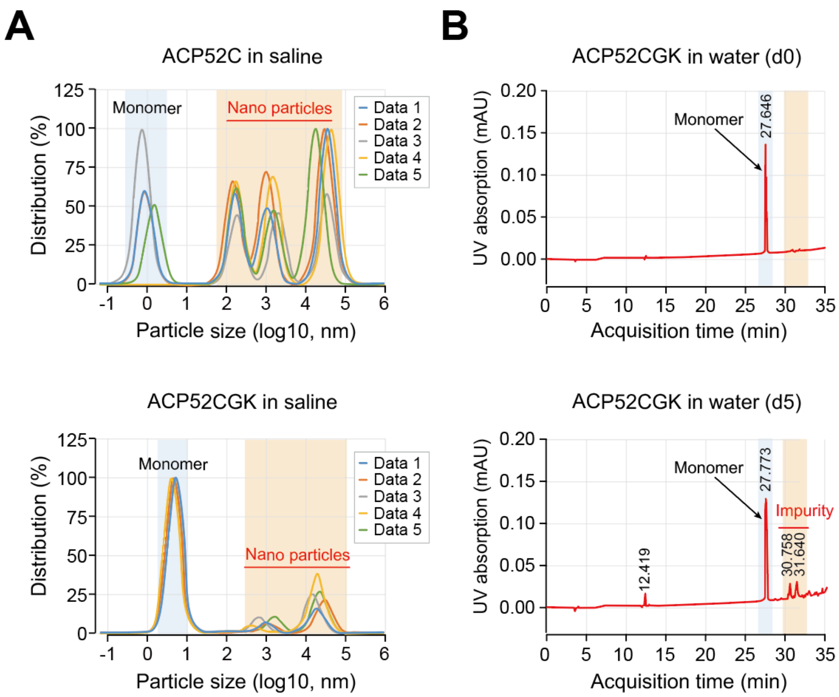

Figure S27. Physicochemical analysis confirms stability and purity issues of ACP52CGK due to inter-strand disulfide bond formation.

A) Dynamic Light Scattering (DLS) analysis was conducted on both ACP52C and ACP52CGK in a saline solution to assess their nanoparticle formation characteristics. The data are presented with five independent measurements to ensure reproducibility. B) The results of HPLC analysis demonstrate the presence of larger particles (i.e., impurities) in ACP52CGK in water over a period of 5 days. C) LC-MS analysis demonstrates the elimination of impurity patterns in ACP52CGK through the use of DTT treatment.

Table S1.
Information of cell lines

Cell line	Source	RRID number	Cat number	Media & supplement
Daudi	blood (B lymphoblast)	RRID:CVCL_0008	ATCC no. CCL-213	RPMI, 10%FBS, P/S
HEL	blood (erythroblast)	RRID:CVCL_0001	ATCC no. TIB-180	RPMI, 10%FBS, P/S
THP1	blood (monocyte)	RRID:CVCL_0006	ATCC no. TIB-202	RPMI, 10%FBS, P/S
HL60	blood (promyeloblast)	RRID:CVCL_0002	ATCC no. CCL-240	RPMI, 10%FBS, P/S
CCRF-CEM	blood (T lymphoblast)	RRID:CVCL_0007	ATCC no. CCL-119	RPMI, 10%FBS, P/S
Molt4	blood (T lymphoblast)	RRID:CVCL_0013	ATCC no. CRL-1582	RPMI, 10%FBS, P/S
Jurkat	blood (T lymphocyte)	RRID:CVCL_0065	ATCC no. TIB-152	RPMI, 10%FBS, P/S
primary T cell	Blood (T lymphocyte)			RPMI, 10%FBS, P/S
K562	bone marrow	RRID:CVCL_0004	ATCC no. CCL-243	RPMI, 10%FBS, P/S
KCL22	bone marrow	RRID:CVCL_2091	ATCC no. CRL-3350	RPMI, 10%FBS, P/S
hCD34+	bone marrow derived progenitor cells			IMDM, 100 ng/ml SCF, 50 ng/ml TPO, supplement
U87MG	brain	RRID:CVCL_0022	ATCC no. HTB-14	DMEM, 10%FBS, P/S
T98G	brain	RRID:CVCL_0556	ATCC no. CRL-1690	RPMI, 10%FBS, P/S
U251	brain	RRID:CVCL_0021	ECACC no. 0861901	RPMI, 10%FBS, P/S
U373MG(= U251)	brain	RRID:CVCL_2818	ATCC no. HTB-17,	RPMI, 10%FBS, P/S
U343	brain	RRID:CVCL_4773		DMEM, 10%FBS, P/S
MCF7	breast	RRID:CVCL_0031	ATCC no. HTB-22	DMEM, 10%FBS, P/S
MDA-MB-231	breast	RRID:CVCL_0062	ATCC no. HTB-26	DMEM, 10%FBS, P/S
MCF10A	breast	RRID:CVCL_0598	ATCC no. CRL-10317	DMEM/F12, 5% HS, 400 ng/ml hydrocortisone, 100 ng/ml cholera toxin, 10 µg/ml insulin, 20 ng/ml EGF, P/S
MDA-MB-468	breast	RRID:CVCL_0419	ATCC no. HTB-132	RPMI, 10%FBS, P/S
SK-BR-3	breast	RRID:CVCL_0033	ATCC no. HTB-30	RPMI, 10%FBS, P/S
HeLa	cervix	RRID:CVCL_0030	ATCC no. CCL-2	DMEM, 10%FBS, P/S
SW620	colon	RRID:CVCL_0547	ATCC no. CCL-227	DMEM, 10%FBS, P/S
SW480	colon	RRID:CVCL_0546	ATCC no. CCL-228	DMEM, 10%FBS, P/S
RKO	colon	RRID:CVCL_0504	ATCC no. CRL-2577	DMEM, 10%FBS, P/S
KM12-SM	colon	RRID:CVCL_9548		DMEM, 10%FBS, P/S
DLD1	colon	RRID:CVCL_0248	ATCC no. CCL-221	RPMI, 10%FBS, P/S
Colo205	colon	RRID:CVCL_0218	ATCC no. CCL-222	RPMI, 10%FBS, P/S
HCT15	colon	RRID:CVCL_0292	ATCC no. CCL-225	RPMI, 10%FBS, P/S
HCT116 (p53 WT)	colon	RRID:CVCL_0291	ATCC no. CCL-247	RPMI, 10%FBS, P/S
HT29	colon	RRID:CVCL_0320	ATCC no. HTB-38	RPMI, 10%FBS, P/S
HCT116 (p53 null)	colon	RRID:CVCL_HD97		RPMI, 10%FBS, P/S
P-MEF	embryonic fibroblast	RRID:CVCL_9115	SCRC-1008	DMEM/F12, 20% serum, 0.1 mM NEAA, 0.1 mM ME, 4 ng/ml bFGF, P/S
hESC	human embryo	RRID:CVCL_9262	US NIH no Miz-hES1	DMEM/F12, 20% serum, 0.1 mM NEAA, 0.1 mM ME, 4 ng/ml bFGF, P/S
293T	kidney	RRID:CVCL_0063	ATCC no. CRL-3216	DMEM, 10%FBS, P/S
Hep3B	liver	RRID:CVCL_0326	ATCC no. HB-8064	DMEM, 10%FBS, P/S
HepG2	liver	RRID:CVCL_0027	ATCC no. HB-8065	DMEM, 10%FBS, P/S
SK-Hep1	liver	RRID:CVCL_0525	ATCC no. HTB-52	DMEM, 10%FBS, P/S
Huh7	liver	RRID:CVCL_0336	JCRB no. 0403	DMEM, 10%FBS, P/S
HepT1	liver	RRID:CVCL_G003		DMEM, 10%FBS, P/S
Hep3B Fluc	liver		ATCC no. HB-8064	DMEM, 10%FBS, P/S
SNU387	liver	RRID:CVCL_0250	ATCC no. CRL-2237	RPMI, 10%FBS, P/S
SNU423	liver	RRID:CVCL_0336	ATCC no. CRL-2238	RPMI, 10%FBS, P/S
SNU354	liver	RRID:CVCL_3947	KCLB no. 00354	RPMI, 10%FBS, P/S
SNU368	liver	RRID:CVCL_3948	KCLB no. 00368	RPMI, 10%FBS, P/S
A549	lung	RRID:CVCL_0023	ATCC no. CCL-185	RPMI, 10%FBS, P/S
HCC827	lung	RRID:CVCL_2063	ATCC no. CRL-2868	RPMI, 10%FBS, P/S
H1975	lung	RRID:CVCL_1511	ATCC no. CRL-5908	RPMI, 10%FBS, P/S
BEAS-2B	lung	RRID:CVCL_0168	ATCC no. CRL-9609	RPMI, 10%FBS, P/S
NCI-H820	lung	RRID:CVCL_1592	ATCC no. HTB-181	RPMI, 10%FBS, P/S
HCC827GR	lung	RRID:CVCL_V620		RPMI, 10%FBS, P/S
HCC827GRKU	lung			RPMI, 10%FBS, P/S
PC9	lung	RRID:CVCL_B260		RPMI, 10%FBS, P/S

PC9GR	lung			RPMI, 10%FBS, P/S
H1299	lung	RRID:CVCL_0060	ATCC no. CRL-5803	RPMI, 10%FBS, P/S
H2009	lung	RRID:CVCL_1514	ATCC no. CRL-5911	RPMI, 10%FBS, P/S
H23	lung	RRID:CVCL_1547	ATCC no. CRL-5800	RPMI, 10%FBS, P/S
H358	lung	RRID:CVCL_1559	ATCC no. CRL-5807	RPMI, 10%FBS, P/S
H920	lung	RRID:CVCL_1599	ATCC no. CRL-5850	RPMI, 10%FBS, P/S
U937	lymphoma	RRID:CVCL_0007	ATCC no. CRL-1593.2	RPMI, 10%FBS, P/S
MEL_DS19	murine blood	RRID:CVCL_2111		DMEM, 10%FBS, P/S
Caov-3	ovary	RRID:CVCL_0201	ATCC no. HTB-75	DMEM, 10%FBS, P/S
SK-OV-3	ovary	RRID:CVCL_0532	ATCC no. HTB-77	RPMI, 10%FBS, P/S
MIA-PACA-2	pancreas	RRID:CVCL_04428	ATCC no. CRL-1420	DMEM, 10%FBS, P/S
PANC1	pancreas	RRID:CVCL_0480	ATCC no. CRL-1469	DMEM, 10%FBS, P/S
DU145	prostate	RRID:CVCL_0105	ATCC no. HTB-81	DMEM, 10%FBS, P/S
PC3	prostate	RRID:CVCL_0035	ATCC no. CRL-1435	RPMI, 10%FBS, P/S
NLCaP	prostate	RRID:CVCL_0395	ATCC no. CRL-1740	RPMI, 10%FBS, P/S
A431	skin	RRID:CVCL_0037	ATCC no. CRL-1555	RPMI, 10%FBS, P/S
HS-746T	stomach	RRID:CVCL_0333	ATCC no. HTB-135	DMEM, 10%FBS, P/S
MKN45	stomach	RRID:CVCL_0434		RPMI, 10%FBS, P/S
hMSC (LT18)	thigh			DMEM-LG, 10%FBS, P/S
Breast cancer patient B3	breast			EpiX media
Colon cancer patient AMC-17CT-033	colon			Organoid media
Colon cancer patient AMC-17CT-045	colon			Organoid media
Liver cancer patient L38	liver			EpiX media
Liver cancer patient L38	liver			EpiX media
Liver cancer patient L42	liver			EpiX media
Liver cancer patient L45	liver			EpiX media
Liver cancer patient L46	liver			EpiX media

RRID, Research Resource Identifiers; ATCC, American type culture collection; ECACC, European collection of authenticated cell cultures; SCRC, stem cell research center; JCRB, Japanese collection of research bioresources cell bank; KCLB, Korea cell line bank. EpiX media, KSFM, 50 µg/mL BPE, 5 ng/mL hEGF, 1 µM A83-01, 5 µM Y27632, 3 µM isoproterenol; Organoid media, DMEM/F12, 10 mM HEPES, 1% GlutaMax-1, 100 µg/mL Primocin, 2% B27 supplement, 1% N2 supplement, 1 mM n-Acetyl-L-cysteine, 10 mM nicotinamide, 500 nM A83-01, 50 ng/mL hEGF, 10 nM gastrin1, 10 µM Y27632, P/S

Table S2.
Oligonucleotide list

name	sequence
for plasmid construction	
hp53_F_MluI	GTT TAA ACA TGG AGG AGC CGC AGT CAG A
hp53_R_NdeI	CAC ATA TGT CAG TCT GAG TCA GGC CCT
hMDM2_F_PmeI	CGT TTA AAC ATG GTG AGG AGC AGG CA
hMDM2_R_SpeI	CCA CTA GTC TAG GGG AAA TAA GTT AG
hYY1_F_PmeI	GTT TAA ACA TGG CCT CGG GCG ACA CC
hYY1_R_SpeI	GCA CTA GTT CAC TGG TTG TTT TTG GC
mCP2c_F_NotI	GCG GCC GCA TGG CCT GGG CTC TGA AG
mCP2c_R_NotI	GCG GCC GCC TTG AGA ATG ACA TGA TA
*sh*hp53_F	GAT CCC CAC CAT CCA CTA CAA CTA CAT TCA AGA GAT GTA GTT GTA GTG GAT GGT TTT TTA
*sh*hp53_R	AGC TTA AAA AAC CAT CCA CTA CAA CTA CAT CTC TTG AAT GTA GTT GTA GTG GAT GGT GGG
*sh*hMDM2_F	GAT CCC CAG GAA TTT AGA CAA CCT GAT TCA AGA GAT CAG GTT GTC TAA ATT CCT TTT TTA
*sh*hMDM2_R	AGC TTA AAA AAG GAA TTT AGA CAA CCT GAT CTC TTG AAT CAG GTT GTC TAA ATT CCT GGG
*sh*hYY1_F	GAT CCC CCG AAT GCT AGA CAA TAG GAT TCA AGA GAT CCT ATT GTC TAG CAT TCG TTT TTA
*sh*hYY1_R	AGC TTA AAA ACG AAT GCT AGA CAA TAG GAT CTC TTG AAT CCT ATT GTC TAG CAT TCG GGG
*sh*hCP2c_F	GAT CCC CCT AGA ATG AAG CCA AGA AAT TCA AGA GAT TTC TTG GCT TCA TTC TAG TTT TTG GAA A
*sh*hCP2c_R	AGC TTT TCC AAA AAC TAG AAT GAA GCC AAG AAA TCT CTT GAA TTT CTT GGC TTC ATT CTA GGG G
*sh*hPIAS1_F	GAT CCC CCA TTC CAC AAC TCA CTT ACT TCA AGA GAG TAA GTG AGT TGT GGA ATG TTT TTA
*sh*hPIAS1_R	AGC TTA AAA ACA TTC CAC AAC TCA CTT ACT CTC TTG AAG TAA GTG AGT TGT GGA ATG GGG
for site directed mutagenesis	
mCP2c (H312A)_F	GAA ACG GCT CGC CCA ACG CCC AGC CAG AAC CAC C
mCP2c (H312A)_R	GGT GGT TCT GGC TGG GCG TTG GGC GAG CCG TTT C
mCP2c (E315A)_F	CCA ACC ACC AGC CAG CAC CAC CCC CTC CAG TC
mCP2c (E315A)_R	GAC TGG AGG GGG TGG TGC TGG CTG GTG GTT GG
mCP2c (D322A)_F	CCC CTC CAG TCA CGG CTA ACC TCT TGC CGA C
mCP2c (D322A)_R	GTC GGC AAG AGG TTA GCC GTG ACT GGA GGG G
mCP2c (E332A)_F	CAA CCA CGC CTC AGG CAG CCC AGC AAT GGC TG
mCP2c (E332A)_R	CAG CCA TTG CTG GGC TGC CTG AGG CGT GGT TG
mCP2c (W336V)_F	CCT CAG GAA GCC CAG CAA GTG CTG CAT CGA AAC CGG
mCP2c (W336V)_R	CCG GTT TCG ATG CAG CAC TTG CTG GGC TTC CTG AGG
mCP2c (H338A)_F	GCC CAG CAA TGG CTG GCT CGA AAC CGG TTC TC
mCP2c (H338A)_R	GAG AAC CGG TTT CGA GCC AGC CAT TGC TGG GC
mCP2c (R339A)_F	CAG CAA TGG CTG CAT GCA AAC CGG TTC TCC AC
mCP2c (R339A)_R	GTG GAG AAC CGG TTT GCA TGC AGC CAT TGC TG
mCP2c (R341A)_F	CAA TGG CTG CAT CGA AAC GCG TTC TCC ACA TTC AC
mCP2c (R341A)_R	GTG AAT GTG GAG AAC GCG TTT CGA TGC AGC CAT TG
mCP2c (R347A)_F	GTT CTC CAC ATT CAC GGC GCT TTT CAC CAA CTT C
mCP2c (R347A)_R	GAA GTT GGT GAA AAG CGC CGT GAA TGT GGA GAA C
mCP2c (D356A)_F	CAA CTT CTC AGG GGC AGC TTT ACT GAA ACT AAC
mCP2c (D356A)_R	GTT AGT TTC AGT AAA GCT GCC CCT GAG AAG TTG
mCP2c (K359A)_F	GGG GCA GAT TTA CTG GCA CTA ACT AGA GAC GAC
mCP2c (K359A)_R	GTC GTC TCT AGT TAG TGC CAG TAA ATC TGC CCC
mCP2c (R362A)_F	GAT TTA CTG AAA CTA ACT GCA GAC GAC GTG ATC CAA ATC
mCP2c (R362A)_R	GAT TTG GAT CAC GTC GTC TGC AGT TAG TTT CAG TAA ATC
mCP2c (D363A)_F	CTG AAA CTA ACT AGA GCC GAC GTG ATC CAA ATC
mCP2c (D363A)_R	GAT TTG GAT CAC GTC GGC TCT AGT TAG TTT CAG
mCP2c (D364A)_F	GAA ACT AAC TAG AGA CGC CGT GAT CCA AAT CTG
mCP2c (D364A)_R	CAG ATT TGG ATC ACG GCG TCT CTA GTT AGT TTC
mCP2c (D373A)_F	CAA ATC TGC GGC CCT GCA GCT GGA ATC AGA CTC TTT AAT G
mCP2c (D373A)_R	CAT TAA AGA GTC TGA TTC CAG CTG CAG GGC CGC AGA TTT G
mCP2c (D376A)_F	CCT GCA GAT GGA ATC GCA CTC TTT AAT GCA TTA AAA G
mCP2c (D376A)_R	CTT TTA ATG CAT TAA AGA GTG CGA TTC CAT CTG CAG G
mCP2c (A380F)_F	GGA ATC AGA CTC TTT AAT TTC TTA AAA GGC CGG ATG G
mCP2c (A380F)_R	CCA TCC GGC CTT TTA AGA AAT TAA AGA GTC TGA TTC C
mCP2c (K382A)_F	GAC TCT TTA ATG CAT TAG CAG GCC GGA TGG TGC G
mCP2c (K382A)_R	CGC ACC ATC CGG CCT GCT AAT GCA TTA AAG AGT C
mCP2c (R384A)_F	CTT TAA TGC ATT AAA AGG CGC GAT GGT GCG GCC AAG
mCP2c (R384A)_R	CTT GGC CGC ACC ATC GCG CCT TTT AAT GCA TTA AAG
mCP2c (R387A)_F	CAT TAA AAG GCC GGA TGG TGG CGC CAA GGC TAA CCA TTT ATG
mCP2c (R387A)_R	CAT AAA TGG TTA GCC TTG GCG CCA CCA TCC GGC CTT TTA ATG
mCP2c (R389A)_F	CGG ATG GTG CGG CCA GCG CTA ACC ATT TAT GTC
mCP2c (R389A)_R	GAC ATA AAT GGT TAG CGC TGG CCG CAC CAT CCG
for reverse transcription quantitative PCR (RT-qPCR)	
mα-globin_qRT_F	GAT CCC AAG TTT TAC TCG GTA GAG CAA GCA CAA ACC AGG
mα-globin_qRT_R	GAT CCC TGG TTT GTG CTT GCT CTA CCG AGT AAA ACT TGG
hCP2c qRT_F	GGT TGG TGC AGG ACT TTG AT
hCP2c qRT_R	CAT GGA GTT TCA CTG CTG GA
hVIM qRT_F	GAA CCA ATG AGT CCC TGG AA
hVIM qRT_R	TCC AGC AGC TTC CTG TAG GT

hZEB1 qRT_F	GCA CAA CCA AGT GCA GAA GA
hZEB1 qRT_R	CAT TTG CAG ATT GAG GCT GA
hSNAI1 qRT_F	CCC CAA TCG GAA GCC TAA CT
hSNAI1 qRT_R	GAC AGA GTC CCA GAT GAG CA
hSNAI2 qRT_F	GTG TCC TTG AAG CAA CCA GG
hSNAI2 qRT_R	TGC GAT GCC CAG TCT AGA AA
hFN1 qRT_F	ACC AAC CTA CGG ATG ACT CG
hFN1 qRT_R	GCT GAT CAT CTG GCC ATT TT
hCD44 qRT_F	CAT CTA CCC CAG CAA CCC TA
hCD44 qRT_R	CCC AGA TGG AGA AAG CTC TG
hEGF qRT_F	CAG GGA AGA TGA CCA CCA CT
hEGF qRT_R	CAG TTC CCA CCA CTT CAG GT
hTGFA qRT_F	CTT TGG GGG TAT TGT GTT GG
hTGFA qRT_R	ACG TAC CCA GAA TGG CAG AC
hOCT4 qRT_F	GGT CCG AGT GTG GTT CTG TA
hOCT4 qRT_R	GGA AAG GGA CCG AGG AGT AC
hNANOG qRT_F	ACC CAG CTG TGT GTA CTC AA
hNANOG qRT_R	GGA AGA GTA AAG GCT GGG GT
hSOX2 qRT_F	AAC CCC AAG ATG CAC AAC TC
hSOX2 qRT_R	CGG GGC CGG TAT TTA TAA TC
hYY1 qRT_F	GAA TTT GCC AGA ATG AAG CC
hYY1 qRT_R	TCA TAG CAG AGT TAT CCC TG
hMDM2 qRT_F	CCT TCC ATC ACA TTG CAA CAG
hMDM2 qRT_R	CAG CTT GTG TTG AGT TTT CCA G
hp53 qRT_F	CGC TTC GAG ATG TTC CGA GA
hp53 qRT_R	CTT CAG GTG GCT GGA GTG AG
hp21 qRT_F	AGC CTG ACA GAT TIC TAT CAC
hp21 qRT_R	CTT TAA GTT TGG AGA CTG GGA
<u>for SNP detection</u>	
hMDM2_SNP_F	TAG CAT TCC TGT GAC TGA GCA
hMDM2_SNP_R	GTC TAC ATA CTG GGC AGG GC
<u>for splicing detection</u>	
hMDM2_Exon3_R	GGT TAC AGC ACC ATC AGT AGG
hMDM2_Exon12_F	GGA CAT CTT ATG GCC TGC TT
<u>for Chromatin immunoprecipitation quantitative PCR (ChIP-qPCR)</u>	
hMDM2 enhancer 1_F	CAA CCT TCT CCT AAC CCT CAC
hMDM2 enhancer 1_R	CAA GGG TTG CAC TAC CGT G
hMDM2 enhancer 2_F	CTG TCT TCG GGT TAG GAG ATC
hMDM2 enhancer 2_R	CCC AAG TCC GCT AGC AG
hMDM2 promoter 3_F	CTT TTG GGT CTG GGC TCT G
hMDM2 promoter 3_R	AAC AAA ACC TCC GCA AAG C
hMDM2 promoter 4_F	GAG TTC AGG GTA AAG GTC ACG
hMDM2 promoter 4_R	CTG CAG TTT CGG AAC GTG
hFN 1-1 promoter_F	GGA CTG TGG GTT CGC AG
hFN 1-1 promoter_R	CCG CTT CCA TCC CTT C
hFN 1-2 promoter_F	TGG GAA GCC GAG TGT TTC
hFN 1-2 promoter_R	CCC AGA ATC AAT GAA TTT TAA GAA AGC
hFN 2 gene body_F	GGC CTA AAG AGG TCT GAA C
hFN 2 gene body_R	TGG TCC TTA CTG CTA CTT ATT TCC
hFN 3 CTCF_F	GAG CTG TTT GGT CTC GAC TC
hFN 3 CTCF_R	AAA GGT TAA TTC AGT ACA AGG TGC
hFN 4 intergenic_F	GCC TTG AAG AGA GTA GTG GTT C
hFN 4 intergenic_R	GTG TGA GGT GTC AGT CTC GC
hVIM 1 promoter_F	AAG ATG TAA CTC AGA GCA TAG GC
hVIM 1 promoter_R	CTC TTT CAC TGG GAG GCT G
hVIM 2 gene body_F	CCG CGT TCC AAT CTC AGG
hVIM 2 gene body_R	GGA AAG AGT AAG GAA TCC CTC AG
hVIM 3 CTCF_F	GGG TTC GGA AGT CAC AGG
hVIM 3 CTCF_R	GAA TGC CTA ATT GTA TCC ACT GG
hVIM 4 intergenic_F	GAG GAC ATG AGA TTT GGG AGG
hVIM 4 intergenic_R	CCA GGT TTT GCT CTT GAC ATA TG
<u>siRNA</u>	
hTHBS siRNA	Santa Cruz, sc-36665
hADM siRNA	Santa Cruz, sc-39273
hTP53I3 siRNA	Santa Cruz, sc-36223
hID3 siRNA	Santa Cruz, sc-38002
hKLF10 siRNA	Santa Cruz, sc-45463
hTNFRSF12A siRNA	Santa Cruz, sc-43764
hGCLM siRNA	Santa Cruz, sc-40603

Table S3.

Drug & peptide list

name	Cat # or sequence
Sorafenib	Sigma-Aldrich, SML2653
CASP2 inhibitor	Ac-VDVAD-CHO
iRGD	CRGDKGPDC
CP2 1P	QEAQQWLHRNRFS
CP2 2P	FSGADLLKLTRDD
CP2 3P	ICGPADGIRLFNA
CP2 4P	VRPRLTIYVCQES
PEP #5	HERRESNYPQRP
Pep #8	HNMHKHSASRIH
Pep #21	HKFHQHRLPHLA
Pep #5-1	HERRES
Pep #5-2	NYPQRP
Pep #5-A	Ac-HERRESNYPQRP-NH2
Pep #5-B	HERRESNYPQRPCRGDKGPDC
Pep #5-C	Ac-HERRESNYPQRPCRGDKGPDC-NH2
Pep #5-2A	Ac-NYPQRP-NH2
Pep #5C (ACP5C)	Ac-HERRESNYPQRPCRGDKGPDC-NH2
Pep #5-2C (ACP52C)	Ac-NYPQRPCRGDKGPDCK-NH2
Biotin-Pep #5-2A	Ac-NYPQRPGGK(biotin)-NH2
Biotin-Pep #5-2C (ACP52CB)	Ac-NYPQRPCRGDKGPDCK(biotin)-NH2
Cy5-ACP52C	cy5.5-aca-NYPQRPCRGDKGPDC-NH2
ACP52-51C	Ac-NYPQRCRGDKGPDC-NH2
ACP52-52C	Ac-YPQRPCRGDKGPDC-NH2
ACP52-4C	Ac-YPQRCRGDKGPDC-NH2
ACP52-4C/1F	Ac-FPQRCRGDKGPDC-NH2
ACP52-4C/1S	Ac-SPQRCRGDKGPDC-NH2
ACP52-4C/1T	Ac-TPQRCRGDKGPDC-NH2
ACP52-4C/2G	Ac-YGQRCRGDKGPDC-NH2
ACP52-4C/3N	Ac-YPNRCRGDKGPDC-NH2
ACP52-4C/4K	Ac-YPQKCRGDKGPDC-NH2
ACP52-31C	Ac-YPQCRGDKGPDC-NH2
ACP52-32C	Ac-PQPCRGDKGPDC-NH2
ACP52D	Ac-KIKKVKKKGRKGSKIKKVKKKGRKGGNYPQRP-NH2
ACP52CG	Ac-K(E-pal)K-GGNYPQRPCRGDKGPDC-NH2
ACP52CK	Ac-K(EGFLG-pal)K-GGNYPQRPCRGDKGPDC-NH2
DHA-ACP52C	DHA-GGNYPQRPCRGDKGPDC-NH2
ACP52GK	Ac-NYPQRPGG-K(E-pal)-GGCRGDKGPDC-NH2
ACP52CGK	Ac-NYPQRPGG-K(γE-pal)-GGCRGDKGPDC-NH2
ACP52CGK-α1	Ac-NYPQRPGGVDVADGGNYPQRPGG-K(γE-pal)-GGCRGDKGPDC-NH2

ACP52CGK-α2	Ac-NYPQRPGGGFLGVDVADGGNYPQRPGG-K(γE-pal)-GGCRGDKGPDC-NH2
ACP52CGK-α3	Ac-NYPQRPGGVDVADGFLGGGNYPQRPGG-K(γE-pal)-GGCRGDKGPDC-NH2
ACP52CGK-α4	Ac-NYPQRPGGNYPQRPGG-K(γE-pal)-GGCRGDKGPDC-NH2
Cy5-ACP52CGK	cy5.5-aca-NYPQRPGG-K(γE-pal)-GGCRGDKGPDC-NH2

Table S4.

Antibody list

immunogen (species)	company	cat #
CP2c (human)	Abcam	ab155238
CP2c (human)	BD Biosciences	610818
CP2b (human)	Santa Cruz	sc-81310
pCDK1 (human)	Cell Signaling	9114
CDK1 (human)	Upstate	06-923
CDK2 (human)	Santa Cruz	sc-163
CDK4 (human)	Upstate	06-139
cyclin A (human)	Abcam	ab38-100
cyclin B1 (human)	Abcam	ab72-100
cyclin D (human)	Santa Cruz	sc-8396
cyclin E (human)	Santa Cruz	sc-198
CHK1 (human)	Santa Cruz	sc-7898
CDC25c (human)	Santa Cruz	sc-13138
p21 (mouse)	Santa Cruz	sc-6246
p53 (human)	Santa Cruz	sc-126
ACTB (human)	Santa Cruz	sc-1616
MCL1 (human)	Santa Cruz	sc-819
BCL2 (human)	BD Biosciences	551109
BCL-XL (human)	Abcam	ab38629
BIM (human)	Santa Cruz	sc-11425
BAX (human)	BD Biosciences	556467
BAK (human)	EMD	AM03
ProCASP3 (human)	Cell Signaling	9665
Cleaved CASP3 (human)	Cell Signaling	9661
ProCASP8 (human)	Santa Cruz	sc-7890
ProCASP9 (human)	Santa Cruz	sc-7885
ProCASP11 (mouse)	Santa Cruz	sc-15881
ProCASP12 (mouse)	Santa Cruz	sc-5627
p73	Abcam	ab189896
p63 (human)	Abcam	ab110038
YY1 (human)	Santa Cruz	sc-7341
MDM2 (human)	Santa Cruz	sc-5304
MDM2 (human)	Genetex	GTX100531
MDM2 (human)	Abcam	ab226939
PSME3 (human)	ZYMED	383800
SUMO1 (human)	Santa Cruz	sc-5308
PIAS1 (human)	Abcam	ab32219
H2AX	Abcam	ab11175
pATM (human)	Abcam	ab81292
CHK2 (human)	Ab Frontier	LF-PA20083
pCHK2 (human)	Ab Frontier	LF-PA20084
ATM (human)	Santa Cruz	sc-135663
TDP2 (human)	Santa Cruz	sc-377280
ATR (human)	Ab Frontier	LF-PA20681
pATR (human)	Abcam	ab227851
pCHK1 (human)	Ab Frontier	LF-PA20082
FBXO31 (human)	Abcam	ab68067
HA	Abcam	ab49969
Flag	Abcam	ab2493
EGFP	Abcam	ab5449
BrdU	Santa Cruz	sc-32323
Hsp60	Ab Frontier	YF-PA12436
LC3	Ab Frontier	LF-PA22271
FLAG-M2	Abcam	ab49763
anti-rabbit IgG-HRP	Ab Frontier	LF-SA8002
anti-mouse IgG-HRP	Thermo Fisher	31430
anti-goat IgG-HRP	Invitrogen	811260
anti-mouse IgG-Cy3	Vectorlabs	CY-2300

anti-rabbit IgG-FITC	Vectorlabs	FI-1000
anti-goat IgG-FITC	Vectorlabs	CY-2500-1
